# Development of prediction models to identify hotspots of schistosomiasis in endemic regions to guide mass drug administration

**DOI:** 10.1073/pnas.2315463120

**Published:** 2024-01-05

**Authors:** Benjamin J. Singer, Jean T. Coulibaly, Hailey J. Park, Jason R. Andrews, Isaac I. Bogoch, Nathan C. Lo

**Affiliations:** ^a^Division of Infectious Diseases and Geographic Medicine, Department of Medicine, Stanford University, Stanford, CA 94304; ^b^Unité de Formation et de Recherche Biosciences, Université Félix Houphouët-Boigny, Abidjan, Côte d’Ivoire; ^c^Centre Suisse de Recherches Scientifiques en Côte d’Ivoire, Abidjan, Côte d’Ivoire; ^d^Swiss Tropical and Public Health Institute, Basel, Allschwil 4123 Switzerland; ^e^University of Basel, Basel 4001, Switzerland; ^f^Department of Medicine, University of Toronto, Toronto, ON M5S 1A8, Canada

**Keywords:** schistosomiasis, hotspots, neglected tropical diseases, machine learning, public health

## Abstract

Schistosomiasis is a neglected tropical disease affecting over 150 million people. Hotspots of *Schistosoma* transmission—communities where infection prevalence does not decline adequately with mass drug administration—present a key challenge in eliminating schistosomiasis. Current approaches to identify hotspots require evaluation 2–5 y after a baseline survey and subsequent mass drug administration. Here, we develop statistical models to predict hotspots at baseline prior to treatment comparing three common hotspot definitions, using epidemiologic, survey-based, and remote sensing data. In a reanalysis of randomized trials in 589 communities in five endemic countries, a regression model predicts whether *Schistosoma mansoni* infection prevalence will exceed the WHO threshold of 10% in year 5 (“prevalence hotspot”) with 86% sensitivity, 74% specificity, and 93% negative predictive value (NPV; assuming 30% hotspot prevalence), and a regression model for *Schistosoma haematobium* achieves 90% sensitivity, 90% specificity, and 96% NPV. A random forest model predicts whether *S. mansoni* moderate and heavy infection prevalence will exceed a public health goal of 1% in year 5 (“intensity hotspot”) with 92% sensitivity, 79% specificity, and 96% NPV, and a boosted trees model for *S. haematobium* achieves 77% sensitivity, 95% specificity, and 91% NPV. Baseline prevalence is a top predictor in all models. Prediction is less accurate in countries not represented in training data and for a third hotspot definition based on relative prevalence reduction over time (“persistent hotspot”). These models may be a tool to prioritize high-risk communities for more frequent surveillance or intervention against schistosomiasis, but prediction of hotspots remains a challenge.

## Significance

Schistosomiasis is a parasitic disease infecting over 150 million people worldwide, with hotspots of high transmission posing a key challenge to elimination efforts. This study develops statistical models to predict three common definitions of hotspots of *Schistosoma* spp. transmission prior to mass treatment campaigns, using epidemiologic, environmental, demographic, and health data. The models predict hotspots with moderate accuracy using a hotspot definition based on prevalence or infection intensity 4 y after treatment initiation but perform less accurately on a hotspot definition based on relative change in prevalence over time. These models, after further validation, may have a future role to prioritize high-risk communities for more frequent surveillance, treatment, and snail control.

Schistosomiasis is a neglected tropical disease caused by the parasite *Schistosoma* spp. that infects over 150 million people globally, mostly in low- and middle-income countries (1, 2). This disease is present across Africa, South America, and Asia leading to inequities in health. Schistosomiasis causes a range of symptoms and pathologies that are specific to the *Schistosoma* species. Chronic infection with *Schistosoma mansoni* can lead to abdominal pain and gastrointestinal symptoms, periportal fibrosis with portal hypertension, and pulmonary hypertension; *Schistosoma haematobium* can lead to hematuria, urogenital pain, infertility, and bladder cancer; both can lead to anemia, malnutrition, and death (3–6).

The World Health Organization (WHO) recommends a public health strategy of annual preventive chemotherapy via mass drug administration for control and elimination of schistosomiasis (7). This strategy applies mass empiric treatment with praziquantel to populations at risk for schistosomiasis. The decision to apply preventive chemotherapy in a geographic region depends on the estimated local prevalence of *Schistosoma* spp. infection, with WHO recommending treatment in communities where district-level prevalence exceeds 10% (7–9). In February 2022, WHO published new public health guidelines on the control and elimination of schistosomiasis that expand treatment from only school-aged children to entire communities, lower the prevalence threshold for treatment, and recommend more frequent treatment in hotspots (7, 10).

Hotspots of *Schistosoma* transmission present a key challenge in the elimination of schistosomiasis. The 2022 WHO guidelines highlight these hotspots and recommend more frequent preventive chemotherapy in these settings and consideration for complementary interventions such as environmental measures to reduce the snail intermediate host of *Schistosoma* spp. (7). Hotspots are defined as high transmission environments where preventive chemotherapy programs (via mass drug administration) fail to reach their goals in terms of reducing infection prevalence and/or intensity. A common definition of “hotspot” is a community that experiences less than an approximate 35% relative reduction in infection prevalence over a 5-y period with over 75% coverage of preventive chemotherapy (7, 11–13). However, alternative definitions of hotspots include settings that have higher than 10% prevalence (the WHO threshold for recommending mass drug administration) or 1% moderate and heavy infection prevalence at the end of a 5 y period with preventive chemotherapy (7, 9, 14). These latter definitions broadly correspond to public health goals set by WHO.

The mechanisms leading to hotspots, under any of these definitions, are not well understood and likely include multiple epidemiologic, behavioral, environmental, and biological factors (12, 13, 15). The *Schistosoma* life cycle relies on a snail intermediate host in fresh water, so environmental factors that affect the ecology of these snails and water sources likely play a role in creating hotspots (4, 16). Some habitats likely sustain the *Schistosoma* life cycle better than others. Infection and transmission occur via human contact with fresh water, and this contact can vary between locations and over time, with some occupations and socioeconomic groups being more at risk for intensive exposure (4, 17). Challenges in participation and equitable distribution of preventive chemotherapy, including treatment fatigue and disruption to local supply chains, can reduce the effectiveness of treatment programs in some locales (18–20). Drug resistance is not yet thought to be a contributing factor in hotspots.

Prior work to identify hotspots often requires waiting 2–5 y after a baseline survey and subsequent preventive chemotherapy for the information necessary to establish a community as a hotspot (11–13). The key challenge with current hotspot detection under this definition is that these settings cannot be identified at baseline and require multiple surveys over time, which delays initiation of more frequent preventive chemotherapy and consideration of alternative control strategies such as snail control. Prior work has studied the prediction of a persistent hotspot based on a definition of relative reduction in prevalence over time (11–13). As prediction of hotspots is a key scientific tool needed for implementation of the new WHO guidelines on mass drug administration, we aim to develop predictive models to identify hotspots after a baseline prevalence survey prior to treatment, with consideration of alternative formulations of the hotspot definition, model selection, and input data.

## Results

### Overview and Descriptive Data.

We investigated hotspots of *S. mansoni* and *S. haematobium* transmission using data from the Schistosomiasis Consortium for Operational Research and Evaluation (SCORE) randomized trials for preventive chemotherapy against schistosomiasis (21, 22). This dataset includes longitudinal epidemiological data on *Schistosoma* infection over 5 y in high-risk communities in five countries: Niger and Mozambique, where data on *S. haematobium* were collected; and Côte d’Ivoire, Kenya, and Tanzania, where data on *S. mansoni* were collected. We included communities that received at least two rounds of preventive chemotherapy with over 75% coverage in the target group(s) and have baseline data in two age-specified subgroups (5–8 and 9–12 y) to ensure representativeness, leading to a final count of 589 communities (*SI Appendix*, Fig. S1). The number of individuals sampled from a given community in year 1 of the trials ranges from 32 to 293, with a median of 195 (*SI Appendix*, Fig. S2). In year 5 of the trials, the number ranges from 60 to 262, with a median of 201 (*SI Appendix*, Fig. S3). Overall year 1 prevalence of infection in included communities was 18.8% for *S. haematobium* and 40.6% for *S. mansoni*. Year 1 prevalence varied between communities from 0% to 93%, with a positively skewed distribution. The average intensity of detected *S. haematobium* infections was 27 eggs per 10 mL of urine, and the average intensity of detected *S. mansoni* infections was 193 eggs per gram of feces. Communities were randomized to different age targeting (school-aged children alone, entire community) and number of rounds of preventive chemotherapy, summarized in Table 1 and *SI Appendix*. More information on demographics and baseline data from this study population is shown in Table 1. We obtained secondary data on environmental, demographic, and health variables from public repositories and datasets (*SI Appendix*, Table S2).

**Table 1.** Description of the study population, baseline *Schistosoma* epidemiology, and prevalence of *Schistosoma* hotspots from randomized trials in five endemic countries

		*S. haematobium* Mozambique	Niger	Total	*S. mansoni* Côte d'Ivoire	Kenya	Tanzania	Total
Study population and trial data								
No. persons		11,161	218,508	229,669	19,381	93,449	77,554	190,384
No. communities		20	210	230	39	197	123	359
Age (%)	5–8	27.1	45.6	44.7	24.2	14.9	27.4	20.9
	9–12	55.2	46.2	46.6	75.4	69.9	65.7	68.8
	13+	17.7	8.2	8.6	0.4	15.1	6.9	10.3
Female (%)		40.4	46.0	45.7	43.1	53.5	52.8	52.2
Mean rounds of treatment		3.1	4.8	4.7	2.8	3.0	3.1	3.0
Baseline epidemiology								
Baseline prevalence (%)		59.1	16.1	18.8	14.3	43.2	44.2	40.6
Baseline mean intensity (eggs per 10 mL urine or 1 g feces)*		105	22	27	86	154	238	193
Baseline moderate and heavy^†^ infection prevalence (%)		13.0	1.0	1.6	1.9	9.9	15.2	11.2
Prevalence of *Schistosoma* hotspots								
Prevalence hotspot (%)		75.0	24.3	28.7	41.0	51.3	84.6	61.6
Intensity hotspot (%)		75.0	13.8	19.1	33.3	48.2	81.3	57.9
Persistent hotspot (%)		40.0	15.2	17.4	28.2	20.8	64.2	36.5

^*^Intensity in units of eggs per 10 mL urine for *S. haematobium* infection, eggs per 1 g feces for *S. mansoni* infection. Mean intensity calculated among infected persons only.

^†^Moderate and heavy infections are defined as >50 eggs per 10 mL urine for *S. haematobium* and >100 eggs per 1 g feces for *S. mansoni*. Prevalence calculated among infected and uninfected persons.

Hotspot definitions: Prevalence hotspot, year 5 prevalence of infection >10%; Intensity hotspot, year 5 prevalence of moderate and heavy infections >1% and prevalence of infection >10%; Persistent hotspot, relative prevalence reduction from year 1 to year 5 <35% and year 5 prevalence of infection >10%. See *Methods* for full description of the hotspot definitions.

**Fig. 1.**
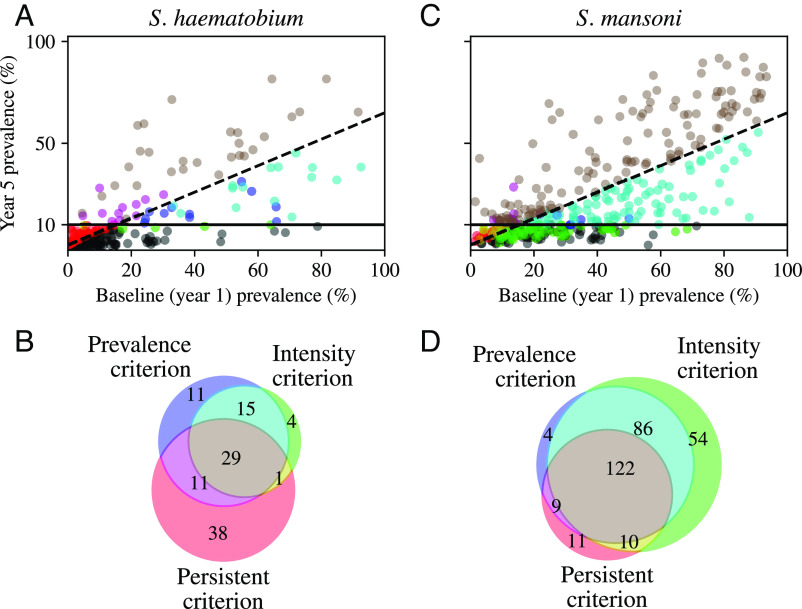
Description of study data and comparison of the three hotspot definitions by *Schistosoma* species. In the scatter plots (panel *A* for *S. mansoni*; panel *C* for *S. haematobium*), each point represents a community, with plotting of baseline prevalence (year 1) and follow-up prevalence (year 5). The two lines indicate thresholds for the prevalence hotspot (solid line) and the persistent hotspot (dashed line). Points are colored according to hotspot classification, as shown in the Venn diagrams in panels *B* and *D*. Prevalence hotspots, where year 5 prevalence >10% (above the solid line), are colored blue, magenta, cyan, or gray. Intensity hotspots, where year 5 prevalence of moderate and heavy infections is >1% and year 5 prevalence is >10%, are colored cyan or gray. Nonhotspot communities where year 5 prevalence of moderate and heavy infections >1% (but overall prevalence <10%) are colored green or yellow. Persistent hotspots, where relative prevalence reduction from year 1 to year 5 <35% and year 5 prevalence >10%, are colored magenta or gray. Nonhotspot communities where relative prevalence reduction from year 1 to year 5 <35% (but overall prevalence <10%) are colored yellow or red. Communities meeting none of these criteria are colored in black in the scatter plots. In the Venn diagrams (panel *B* for *S. mansoni*, panel *D* for *S. haematobium*), the number of communities for each species meeting the hotspot criteria (prevalence, intensity, persistent) is shown. In the final analysis, all hotspot definitions were required to meet the prevalence criterion of year 5 prevalence >10%. Communities meeting no hotspot criterion are not included in the Venn diagrams.

We used three distinct definitions of hotspot evaluated after 4 y of preventive chemotherapy. First, “prevalence hotspot”, based on the WHO goal for schistosomiasis control, includes any community with >10% prevalence of *Schistosoma* spp. infection after 4 y of a preventive chemotherapy program (measured in year 5) (7, 14). Overall frequency of prevalence hotspots is 28.7% in *S. haematobium* endemic countries and 61.6% in *S. mansoni* endemic countries. Second, “intensity hotspot” includes any community with >1% prevalence of moderate- and heavy-intensity *Schistosoma* spp. infections and >10% overall infection prevalence after 4 y of a preventive chemotherapy program (measured in year 5). Moderate- and heavy-intensity infections include all *S. haematobium* infections producing more than 50 eggs per 10 mL of urine, and *S. mansoni* infections producing more than 100 eggs per gram of feces. Overall frequency of intensity hotspots is 19.1% in *S. haematobium* endemic countries and 57.9% in *S. mansoni* endemic countries. Third, “persistent hotspot”, based on previous work to characterize and predict hotspots, includes any community with less than a 35% relative reduction in prevalence of *Schistosoma* spp. infection and >10% overall infection prevalence after 4 y of a preventive chemotherapy program (measured in year 5) (11–13). Overall frequency of persistent hotspots is 17.4% in *S. haematobium* endemic countries and 36.5% in *S. mansoni* endemic countries. For each definition, we required that for a community to be classified as a hotspot, its prevalence of *Schistosoma* infection in year 5 exceeded 10% to fulfill the WHO recommendation on threshold for continued mass drug administration, which means that all intensity and persistent hotspots also match the definition of a prevalence hotspot (7, 14). Further information on the prevalence of hotspots under these definitions is given in Table 1, and the overlap between the definitions is illustrated in Fig. 1.

### Overall Model Performance.

We tested seven classification models to predict intensity and persistent hotspots and fourteen models (classification and regression) to predict prevalence hotspots, using epidemiological data from SCORE combined with remote sensing and survey-based secondary datasets. We followed three different approaches for model training and validation, for each species of *Schistosoma*—generating the “combined-countries,” “within-country,” and “between-countries” test sets (see *Methods, Model development and validation*). Each approach provides different splits of the data for model development (“training set,” e.g., 70% of data, for cross-validation) and 30% for model performance evaluation of the final model (“test set,” e.g., 30% of data). Full model performance statistics are given in *SI Appendix*, Tables S5–11 and Fig. S24 with an abbreviated version of these data shown in Table 2.

**Table 2.** Statistical model performance to predict *Schistosoma* hotspots in the combined-country test set

		*S. haematobium* Accuracy	Sensitivity	Specificity	*S. mansoni* Accuracy	Sensitivity	Specificity
Prevalence hotspot	Best model	90 (81–95)	90 (71–97)	90 (78–95)	81 (73–88)	86 (75–92)	74 (60–85)
	Ensemble	86 (75–92)	67 (45–83)	94 (83–98)	81 (73–88)	83 (72–90)	79 (65–89)
Intensity hotspot	Best model	91 (82–96)	77 (50–92)	95 (85–98)	86 (78–91)	92 (82–96)	79 (66–88)
	Ensemble	90 (81–95)	62 (36–82)	96 (88–99)	82 (74–88)	83 (72–91)	81 (68–90)
Persistent hotspot	Best model	87 (77–93)	55 (28–79)	93 (84–97)	74 (65–81)	56 (41–70)	85 (74–92)
	Ensemble	83 (72–90)	18 (5–48)	95 (86–98)	72 (63–80)	37 (24–52)	94 (85–98)

Model performance measures calculated in the combined-countries test set; results are reported with 95% CI in parentheses.

Best model defined as model with the highest accuracy.

Ensemble model defined as the ensemble of all six classifier models with hard voting.

Sensitivity is synonymous with recall and true positive rate (TPR). Specificity is equal to the complement of the false positive rate. ROC curves are included in *SI Appendix*.

For the prevalence hotspot definition, models for *S. haematobium* have an accuracy of 83–90% in the combined-countries test set, with the most accurate model (linear regression) having 90% sensitivity and 90% specificity. In the within-country set, accuracy is 75–92% with the most accurate model (random forest classification) having 88% sensitivity and 94% specificity. In the between-countries set, accuracy is 25–85% with the most accurate model (logistic regression) having 87% sensitivity and 80% specificity. The analogous figures for *S. mansoni* are 70–81% accuracy in the combined-countries set with the most accurate model (elastic net logistic regression) having 86% sensitivity and 74% specificity and 68–86% accuracy in the within-countries sets with the most accurate model (boosted classification trees) having 88% sensitivity and 85% specificity; model performance in the between-countries sets is very poor (*SI Appendix*, Tables S5–S11).

Model performance when predicting the intensity hotspot definition is similar. For the intensity hotspot definition, models for *S. haematobium* have an accuracy of 84–91% in the combined-countries test set, with the most accurate model (boosted classification trees) having 77% sensitivity and 95% specificity. In the within-country set, accuracy is 81–86% with the most accurate model (support vector classification) having 45% sensitivity and 94% specificity. In the between-countries set, accuracy is 25–80% with the most accurate model (boosted classification trees) having 87% sensitivity and 60% specificity. The analogous figures for *S. mansoni* are 64–86% accuracy in the combined-countries set with the most accurate model (random forest classification) having 92% sensitivity and 79% specificity, 72–83% accuracy in the within-countries sets with the most accurate model (elastic net logistic regression) having 78% sensitivity and 89% specificity, and 53–84% accuracy in the between-countries sets with the most accurate model (boosted classification trees) having 84% sensitivity and 83% specificity.

Model performance when predicting the persistent hotspot definition is poor overall. For the persistent hotspot definition, models for *S. haematobium* have an accuracy of 81–87% in the combined-countries test set, with the most accurate model (boosted classification trees) having 55% sensitivity and 93% specificity. In the within-country set, accuracy is 78–87% with the most accurate model (support vector classification) having 45% sensitivity and 96% specificity. The analogous figures for *S. mansoni* are 64–74% accuracy in the combined-countries set with the most accurate model (boosted classification trees) having 56% sensitivity and 85% specificity, 42–81% accuracy in the within-countries sets with the most accurate model (support vector classification) having 47% sensitivity and 93% specificity. For both species, model performance in the between-countries sets is very poor (*SI Appendix*, Tables S5–S11).

**Fig. 2.**
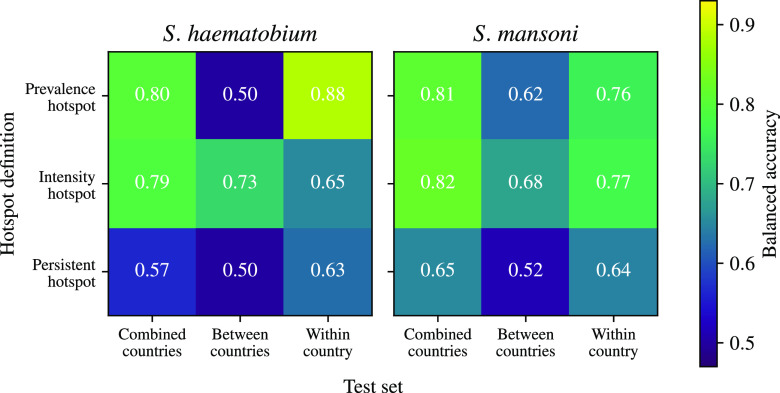
Comparison of balanced accuracy of the ensemble classifier model to predict hotspots of *Schistosoma* transmission by species, hotspot definition, and test set. The ensemble classifier models combined predictions from all six classification models with hard voting. The analysis is designed to provide an overall summary of model performance comparing between hotspot definitions and test sets. The balanced accuracy is the mean of sensitivity and specificity of each ensemble model, providing a reliable indicator of model performance. For the between-countries and within-country test sets in the *S. mansoni* panel, which include multiple countries in the test set, we show the mean balanced accuracy (weighted by test set size).

Table 2 includes results for the ensemble classification model for each hotspot definition, which generally does not outperform the best model. Fig. 2 compares the balanced accuracy (mean of sensitivity and specificity) of an ensemble classification model for each prediction task, finding that this model typically performs best in the combined-countries set.

### Highlighted Models.

**Fig. 3.**
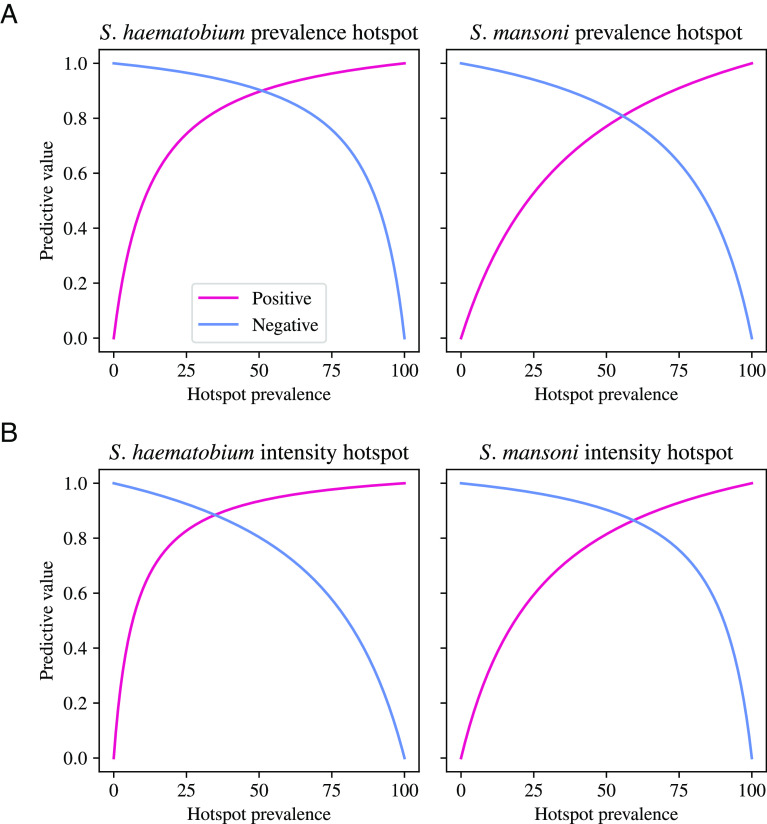
Positive and negative predictive values of models to predict *Schistosoma* hotspots under the prevalence and intensity hotspot definitions using the combined-countries test set. The positive and negative predictive values are calculated for a full range of hotspot prevalence values for the prevalence hotspot definition (*A*) and intensity hotspot definition (*B*). The model for *S. haematobium* prevalence hotspots is a linear regression model with forward variable selection, and the model for *S. mansoni* prevalence hotspots is a logistic regression model with elastic net regularization. The model for intensity hotspots for *S. haematobium* is a boosted trees classifier, and the model for intensity hotspots for *S. mansoni* is a random forest classifier

Here, we highlight models with a good balance of accuracy, simplicity, and interpretability. For predicting prevalence hotspots of *S. haematobium*, we find that the linear regression model with forward variable selection, using the prevalence hotspot definition in the combined-countries test set, is parsimonious and has high performance. In this prediction task, a single predictor was chosen in the variable selection process—the all-ages baseline prevalence of infection. The resulting linear regression has 90% accuracy (95% CI: 81–95%), 90% (71–97%) sensitivity, and 90% (78–95%) specificity. In a setting with 30% hotspot prevalence, this model would have a 96% negative predictive value (NPV; Fig. 3 and *SI Appendix*). This model predicts that a community will be a hotspot if its baseline prevalence is above 16.4%.

For predicting prevalence hotspots of *S. mansoni*, we find that the logistic regression model with elastic net regularization, using the prevalence hotspot definition in the combined-countries test set, has moderate performance. This model has 81% (73–88%) accuracy, 86% (75–92%) sensitivity, and 74% (60–85%) specificity. In a setting with 30% hotspot prevalence, this model would have 93% NPV (Fig. 3). The three variables with the highest magnitude coefficients were: 1) prevalence of infection in children aged 9–12, 2) coverage of the third dose of the diphtheria, pertussis, and tetanus (DPT) vaccine (a proxy for healthcare access), and 3) average years of education among males. The full coefficients are given in *SI Appendix*, Table S2 and Fig. S19.

### Sensitivity Analyses and Role of Secondary Data.

We estimated the contribution of secondary (remote sensing and survey-based) data on the model performance by rerunning the analysis with these data excluded. The accuracy of the best model (as evaluated in the analysis with all variables) in the combined-countries test set is improved by 0 to 13 percentage points by the availability of secondary data (Table 3). The benefit of secondary data is larger when predicting *S. mansoni* hotspots. Not all models are improved by the availability of secondary data, as seen in the case of the ensemble classifier model, whose accuracy changes −6 to 7 percentage points with the availability of secondary data. Full tables of model performance when secondary data are excluded are given in *SI Appendix*, Tables S15–S18. Summaries of variable importance in some models are given in *SI Appendix*, Figs. S16–S23.

**Table 3.** Difference in performance of statistical models to predict *Schistosoma* hotspots from the main analysis compared to models excluding secondary data sources

		*S. haematobium* Accuracy	Sensitivity	Specificity	*S. mansoni* Accuracy	Sensitivity	Specificity
Prevalence hotspot	Best model	0.00	0.00	0.00	13.08	4.69	25.58
	Ensemble	−5.80	−14.29	−2.08	6.54	1.56	13.95
Intensity hotspot	Best model	5.80	38.46	−1.79	4.67	13.56	−6.25
	Ensemble	2.90	15.38	0.00	1.87	1.69	2.08
Persistent hotspot	Best model	1.45	36.36	−5.17	4.67	34.15	−13.64
	Ensemble	−1.45	18.18	−5.17	2.80	9.76	−1.52

Model performance calculated in the combined-countries test set. Best model defined as model with the highest accuracy. Ensemble model defined as the ensemble of all six classifier models with hard voting.

A positive value indicates that the statistical model from main analysis (including secondary data sources) outperforms the corresponding model excluding secondary data (i.e., only using epidemiologic data). A negative value indicates the opposite.

Sensitivity is synonymous with recall and TPR. Specificity is equal to the complement of the false positive rate. ROC curves are included in *SI Appendix*.

We repeated the analysis using alternative split of dataset for training set (60%) and hold-out set (20%) for model development, with model evaluation on a test set (20%) (*SI Appendix*, Tables S19–S22). The performance is slightly worse overall, with wider CIs. We repeated the analysis adjusting for imperfect diagnostic sensitivity (*SI Appendix*, Tables S23–S26), with worse performance overall than using uncorrected prevalence.

## Discussion

In this study, we developed statistical models to predict hotspots of *Schistosoma* transmission to inform precision targeting in high transmission settings. The new 2022 WHO guidelines for schistosomiasis recommend expanded preventive chemotherapy and aspire to a goal of elimination, so it will become increasingly important to recognize and target hotspots with additional interventions to reduce transmission. We found that statistical models predict hotspots of *Schistosoma* transmission with moderate accuracy at baseline prior to subsequent preventive chemotherapy, using a hotspot definition based on year 5 prevalence (prevalence hotspot) or year 5 infection intensity (intensity hotspot)—though prediction remains challenging. High performance requires local epidemiologic data, with additional data from multiple regions improving performance. Model performance for predicting relative prevalence reduction over time (persistent hotspot) is weaker. Our best performing models have a high NPV in some settings to rule out hotspots and may have a future role, after further validation, to prioritize high-risk communities for more frequent surveillance or evaluation for therapy with (semiannual) chemotherapy and snail control.

Our study builds on prior work investigating prediction of persistent hotspots in SCORE data (11, 12). Prior work has demonstrated that identifying robust relationships between baseline prevalence and year 5 prevalence is challenging (12), and has often relied on additional prevalence surveys for data collection (at year 3) to improve this prediction (11). The present study provides three important contributions beyond prior work. First, we investigated hotspot prediction for both *S. haematobium* and *S. mansoni* with a wide range of statistical models. Second, we systematically studied the use of alternative hotspot definitions (prevalence hotspot, intensity hotspot). Third, we used publicly available geospatial secondary datasets. These contributions yield some improvement to prediction at baseline. A primary goal of this study was to compare the predictive performance and strengths and limitations of three common definitions for hotspots. Ultimately, the final choice of hotspot definition and its implementation should be made by WHO and national control programs for their public health goals, and this study aims to provide data to inform their choice.

In general, the prevalence hotspot, i.e., a community in which *Schistosoma* infections persist at >10% prevalence 5 y after preventive chemotherapy begins, is more predictable than the persistent hotspot. This prevalence hotspot definition based on absolute *Schistosoma* infection prevalence is parsimonious and has clear public health importance (14). 10% is the WHO-recommended prevalence threshold for preventive chemotherapy, and achieving reduction below this threshold would potentially change the recommended control strategy. Previous studies of schistosomiasis hotspots have used a relative prevalence change definition (persistent hotspot), in which a hotspot is defined as a community which experiences <35% relative reduction in prevalence 4 y after chemotherapy begins (11–13). In this study, consistent with prior work, we found that this persistent hotspot definition was overall more challenging to predict than the prevalence hotspot (Fig. 2). The persistent hotspot definition includes an implicit assumption about the dynamics of schistosomiasis—that, irrespective of measurement error and stochasticity, the prevalence 4 y after initiation of chemotherapy is proportional to the baseline prevalence, at least in nonhotspot communities. Given the complexity of schistosomiasis transmission dynamics, this assumption may not hold, which may explain why machine learning models often perform poorly when predicting the persistent hotspot definition (11–13, 23–25). The intensity hotspot is also easier to predict, but somewhat less parsimonious than the prevalence hotspot, requiring more data to define.

Different data are required for different models and hotspot definitions, and ultimately quality of data is more important than the choice of statistical model. In general, most variation in accuracy between different models for a single prediction task was within the 95% CIs, so can be explained by random binomial variation. This suggests that the choice of the statistical model is less important than the available data and choice of hotspot definition. As previously noted by Shen et al. (11), prediction of persistent hotspots in countries not represented in the training data often performs poorly overall, therefore our findings support the need for robust data for model development for each country in which prediction takes place. The simplest model we developed was a linear regression model for *S. haematobium* to predict prevalence hotspots, with a single predictor variable of prevalence. This model identifies a straightforward cutoff of 16.4% baseline prevalence to predict prevalence hotspot status, although future validation will be important. Prevalence is the most likely variable to be recorded in available epidemiologic data, and while it is an obvious feature to use in determining treatment strategies, previous studies have failed to establish a well-defined relationship between baseline prevalence and (persistent) hotspot status (11, 12). Our model for *S. mansoni* prevalence hotspots is more complex than for *S. haematobium*, using both prevalence and intensity data. Age-structured variables and the survey-based variables also have large coefficients in this model. Prediction of *S. haematobium* hotspots was more tractable than prediction of *S. mansoni* hotspots irrespective of model choice. This may be due to some feature of *S. haematobium* transmission dynamics or related to the available data and its distribution of values or country factors specific to the dataset.

We have developed predictive machine learning models for detecting hotspots with high NPV in settings with low to moderate prevalence of hotspots (~30%). A model with high NPV could improve resource allocation by deprioritizing certain communities which are predicted to reach prevalence targets without special interventions (i.e., nonhotspots). If preventive chemotherapy is lower cost than surveillance, as is often the case for schistosomiasis, predicted hotspots could be considered for more intensive intervention (26, 27). In all scenarios, some level of surveillance will still be necessary to ensure that this increased intervention is successful in reducing prevalence to target levels. These models could also have a role in guiding surveillance.

While WHO recommends more frequent preventive chemotherapy in hotspots, the optimal intervention for control in these settings remains an area of ongoing research. The strategy of preventive chemotherapy rarely interrupts transmission and reinfection rates often remain high. A key limitation in our study is lack of data on the snail intermediate host populations, which are critical in transmission. In addition to chemotherapy, public health responses to hotspots can include snail control measures (which have a historic role in controlling schistosomiasis in China) (27) and water, sanitation, and hygiene (WASH) interventions (7, 13, 28–30). Recent innovative studies have identified ecological interventions to reduce the snail population, including removal of vegetation that facilitates snail habitats or introduction of predatory prawns, which have both led to reductions in schistosomiasis (31, 32).

There are several limitations affecting the generalizability of our models to new settings. The SCORE study was specifically targeted to communities and regions which were believed to have high prevalence of schistosomiasis, so our models were trained and tested on datasets likely to have an unusually high proportion of hotspots (21). The number of communities included from each country varied from 20 to 210, potentially distorting comparisons between different species and test sets. Some test sets had a small sample size, which may preclude robust model evaluation. As preventive chemotherapy is applied in more regions, most endemic communities will have a long history of receiving treatment, which may change infection dynamics. Our models require local epidemiologic data that is resource intensive to collect, and the models are not reliable in places which lack schistosomiasis data. In SCORE, different preventive chemotherapy strategies were applied in different communities; while we adjusted for this in our models, we may not capture all differences between the treatment arms. The dynamics underlying the persistence of hotspots, under any definition, are likely to be highly heterogeneous and may vary widely between settings (15–20, 23, 31–33). Beyond the variables included in this study, there are many factors at the individual level (e.g., contact with water, occupation, acceptance of treatment, travel history) and environmental level (e.g., snail density, infection prevalence in snails) that may be predictive of hotspots. Data on these variables were not available for this study, and future work could consider further data collection to study these factors. Diagnostic sensitivity for *Schistosoma* spp. is imperfect, but we limit the impact of this by following WHO guidelines that define prevalence (and therefore hotspots) based on use of these imperfect diagnosis (7). Further limitations include the large number of models evaluated (producing issues with multiple comparisons), potential overfitting, and the generally large CIs on model performance. The goal of this study was prediction and not causal inference. Overall, the predictive models we present will require external validation before widespread use—this should involve prospective model validation with longitudinal data collection.

Our study finds that statistical models may have a role in predicting hotspots of *Schistosoma* transmission with high NPV in some settings, during the baseline survey prior to preventive chemotherapy. These models may therefore have a future role to prioritize high-risk settings.

## Methods

### Data.

We used data from the SCORE cluster randomized controlled trials for preventive chemotherapy against schistosomiasis with corresponding study dates from 2011 (year 1) to 2015 (year 5), accessed via public repository (21, 22). We used available data on *S. haematobium* and *S. mansoni*. For *S. haematobium*, the SCORE project includes two distinct 5-y randomized trials in Niger and Mozambique which enrolled a total of approximately 315,000 participants (34–39). For *S. mansoni*, the SCORE project includes a 5-y randomized trial which enrolled approximately 233,000 participants from three countries (Côte d’Ivoire, Kenya, and Tanzania) (37, 40–44). Each trial evaluated six distinct preventive chemotherapy strategies for schistosomiasis, with parasitological outcomes measured at several points in time (*SI Appendix*). Inclusion criteria of source data for our prediction model were applied at the unit of a community. These criteria are 1) baseline prevalence data available in 5- to 8- and 9- to 12-y-olds and 2) treatment via praziquantel distributed at least twice to school-aged children with high coverage (>75%), following WHO’s definition of adequate therapy (7) (*SI Appendix*, Fig. S1).

We also used environmental, demographic, and health data from survey-based and remote sensing datasets not included in the SCORE study. A summary of these data is given in *SI Appendix*, Table S2 and Figs. S6–S10. These data sources included population density data from CIESIN (45), surface water data from CGLS (46), precipitation and temperature data from WorldClim (47), vegetation data from MODIS (48), and health and socioeconomic survey data from the Demographic and Health Surveys (49) (https://dhsprogram.com/).

### Study Outcomes.

The study outcomes are defined as follows (Fig. 1).

1) A prevalence hotspot is any community with >10% prevalence of *Schistosoma* spp. infection after 4 y of a preventive chemotherapy program (measured in year 5).

2) An intensity hotspot is any community with >1% prevalence of moderate- and heavy-intensity *Schistosoma* spp. infections and >10% overall infection prevalence after 4 y of a preventive chemotherapy program (measured in year 5).

3) A persistent hotspot is any community with less than a 35% relative reduction in prevalence of *Schistosoma* spp. infection from year 1 to year 5 and >10% overall infection prevalence after 4 y of a preventive chemotherapy program.

### Model Development and Validation.

We used three approaches for model development and validation; each approach provided: 1) a training dataset for model development using fivefold cross-validation (e.g., 70% of dataset, with model evaluation on a hold-out set during cross-validation) and 2) a test dataset for model performance evaluation of the final model (e.g., 30% of dataset; unseen during model development, sometimes referred to as the “validation set”). The reported study outcomes were calculated on the test dataset.

First, the combined-countries approach included data from all the countries for a given species, with data from 70% of communities in the training set for model development (using fivefold cross-validation, with model evaluation on a hold-out set during cross-validation), and the remaining 30% in the test set for model performance evaluation of the final model. The *S. haematobium* analysis combined data from Niger and Mozambique, and the *S. mansoni* analysis combined data from Côte d’Ivoire, Kenya, and Tanzania.

Second, the within-country approach included data from a single country at a time, with data from 70% of communities in the training set for model development (using fivefold cross-validation), and the remaining 30% used for the test set for model performance evaluation of the final model. We required that the training dataset contain at least 100 communities. For this approach, only Niger, Kenya, and Tanzania datasets included enough communities to perform the analysis. See *SI Appendix*, Table S1 for a more detailed summary of the dataset split for model training and validation.

Third, the between-countries approach included data from all except one country for the training set for model development (using cross-validation), and the test set for model performance evaluation used data from the remaining country. We required that the training dataset contain at least 100 communities. For this approach, the training set for *S. haematobium* used data from Niger (model development used fivefold cross-validation), with the test set for evaluation of the final model using data from Mozambique. For *S. mansoni* under this approach, we performed three alternative data splits: 1) data from Côte d’Ivoire and Kenya in the training set (model development used groupwise cross-validation by country), with data from Tanzania as the test set; 2) data from Kenya and Tanzania in the training set (with groupwise cross-validation), with data from Côte d’Ivoire as the test set; 3) data from Tanzania and Côte d’Ivoire in the training set (with groupwise cross-validation), with data from Kenya as the test set.

As an alternative analysis, we split data into a training set (60% of data) and hold-out set (20% of data) for model development, with evaluation of model performance (test set) on the remaining 20% of data. For the combined-countries version of this analysis, the test set was of a fixed size, but the model development set was likewise split 60:20 (3:1) into training and hold-out sets. This was not chosen for the main analysis due to the small size of the test sets.

### Statistical Analysis.

We developed up to 14 statistical learning models to predict hotspots of *Schistosoma* spp. under three different hotspot definitions at a community level. For all hotspot definitions, we used classification models to predict the binary outcome of hotspot status: logistic regression with forward variable selection, elastic net logistic regression, boosted classification trees, random forest classification, support vector classification, multilayer perceptron classification, and an ensemble of all six models using hard voting. For the prevalence hotspot definition, hotspot status is defined by a single continuous variable (prevalence in year 5), so in addition to the seven classification models listed above, we used seven regression models: linear regression with forward variable selection, elastic net linear regression, boosted regression trees, random forest regression, support vector regression, multilayer perceptron regression, and an ensemble of all six models. We dichotomized the outputs of the regression models into hotspot and nonhotspot categories using a threshold of 10% prevalence.

We used eleven primary variables from the SCORE dataset as predictors that were available at year 1 (prior to preventive chemotherapy). Variables 1–3 are related to infection prevalence, defined as the proportion of tested individuals with detectable *Schistosoma* eggs: 1) prevalence in the 5- to 8-y-old age group, 2) prevalence in the 9- to 12-y-old age group, and 3) prevalence in the total tested population. Variables 4–6 describe the mean infection intensity, in persons with detectable eggs (i.e., infected persons), defined as the arithmetic mean number of *S. haematobium* eggs per 10 mL of urine, or the mean number of *S. mansoni* eggs per gram of stool. These variables are likewise split into 4) the 5- to 8-y-old age group, 5) the 9- to 12-y-old age group, and 6) the total tested population. Variable 7 describes the variability in infection intensity, using the dispersion parameter of the maximum likelihood negative binomial distribution fit to intensity (eggs per 10 mL of urine or gram of stool) in persons with detectable eggs. To make the fit more stable, we do not stratify this variable by age. Variables 8 and 9 are interaction variables between infection prevalence and infection intensity, using alternately 8) the arithmetic and 9) the geometric mean of infection intensity. Finally, variables 10 and 11 describe the praziquantel treatment to be administered over the course of the study as determined by the study arm: 10) the number of rounds of treatment including distribution to school-aged children, and 11) the number of rounds of treatment including distribution to the entire community. Country was not included as a predictor.

For each community, we included fourteen additional predictors defined using secondary (non-SCORE) data from remote sensing and survey-based datasets. The choice of these variables was broadly informed by prior literature and expert opinion (1, 50). Remote sensing variables include density of vegetation (measured using the normalized difference vegetation index), the proximity of the community to a body of fresh water, the annual precipitation, the average temperature of the coldest month, the population density, and the infection density (the population density multiplied by the prevalence of *Schistosoma* infection) (45–48). Survey-based variables (derived from geospatial models of Demographic and Health Surveys data) include proportion with access to improved water, proportion with access to improved sanitation, mean level of education, mean wealth index (standardized within a country), proportion with receipt of a third dose of DPT vaccine (a common proxy for healthcare access), proportion of children under 5 y old who are underweight, proportion of children under 5 y old who are stunted, and mortality in children under 5 y (49, 51–55). More information on these variables is available in *SI Appendix*, Table S1 and Figs. S6–S10. We selected values from the year 2011 when available, which includes all variables except for distance to a body of fresh water, where we used the value for 2020.

For skewed variables, we took the natural logarithm of the raw values. These variables include all intensity variables, the dispersion of intensity, the interaction variables between intensity and prevalence, the distance to fresh water, the population density, and the interaction variable between population density and prevalence. In model development, all variables were scaled to have zero mean and a SD of one. We evaluated bivariate relationships in the combined-countries training set between each variable and hotspot definition (*SI Appendix*, Table S14). We implemented all models using the Scikit-learn Python package (version 1.1.1) and Python (version 3.9.7). We used the XGBoost package (version 1.7.6) to implement boosted trees models (56). We follow reporting per the TRIPOD checklist (*SI Appendix*) (57).

The steps for model development were specific to the statistical model, including selection of model hyperparameters and model variables. To set the hyperparameters of the models and to inform variable selection when applicable, we used fivefold cross-validation in the training set (except for the between-countries sets for *S. mansoni*, where we perform subgroup cross-validation by country). We chose the hyperparameters which gave the highest mean accuracy in the cross-validation. For variable selection in the linear regression and logistic classification models, we included a 1% parsimony threshold, i.e., we chose, from the set of models identified by forward variable selection, the model with the smallest number of variables whose accuracy was no more than one percentage point worse than the optimal model. We included this parsimony threshold to reduce variance in these models and improve their potential generalizability.

We estimated model performance on the test dataset by computing classification accuracy, sensitivity (synonymous with recall and TPR), specificity (equivalent to 1 minus the false positive rate), balanced accuracy (mean of sensitivity and specificity), and the F1 score (harmonic mean of sensitivity and PPV). We found 95% CIs for these quantities using the Wilson score technique, which gives asymmetric intervals around the mean. We used the calculated values of sensitivity and specificity to calculate negative and positive predictive value for a range of hotspot prevalence. We computed the ROC curves for the classification models and the area under these curves as an additional model performance measure. In our development process decisions on model inclusion and featuring were largely based on the balanced accuracy, which provides robust and comparable measure of performance in multiple unbalanced test sets.

We performed multiple sensitivity analyses and additional analyses. To evaluate the role of secondary variables in model performance, we developed predictive models excluding secondary variables from the training data. To investigate the effect of different random samples of the training and test datasets on model performance, we resampled the dataset split 500 times and repeated the model development and evaluation of model performance. More information on resampling is included in *SI Appendix*, Figs. S25–S28. We repeated the main analysis correcting for imperfect sensitivity for single and double stool Kato–Katz examination (stool microscopy) for *S. mansoni* and urine microscopy for *S. haematobium* (*SI Appendix*).

### Ethical Approval.

This study was approved by the institutional review board at Stanford University. This study used a secondary dataset without direct identifiers.

## Supplementary Material

Appendix 01 (PDF)

## Data Availability

Analytic code is available online (58), and study data are available through public repositories as detailed in *SI Appendix*, Tables S1 and S2 (22, 34, 35, 40, 45, 48, 59–62).
