# Supplementary material for: Development of prediction models to identify hotspots of schistosomiasis in endemic regions to guide mass drug administration
Source: Proc Natl Acad Sci U S A. 2024 Jan 5;121(2):e2315463120. doi: 10.1073/pnas.2315463120 (PMC10786280; doi:10.1073/pnas.2315463120)
Supplement: Supplementary file 1 — Appendix 01 (PDF) [file pnas.2315463120.sapp.pdf]

Supporting Information

Development of prediction models to identify hotspots of schistosomiasis in endemic regions to guide mass drug administration

Benjamin J. Singer, Jean T. Coulibaly, Hailey J. Park, Jason R. Andrews, Isaac I. Bogoch, and Nathan C. Lo

Table of Contents

Technical Appendix
- Background on SCORE randomized trials
  - Table S1: SCORE variables
- Data processing
  - Table S2: Secondary variables
- Statistical analysis
- Sensitivity analysis

Supplemental Tables and Figures
- Figure S1: Flow charts of data exclusions.
- Figure S2: Histograms of community sample sizes (year 1).
- Figure S3: Histograms of community sample sizes (year 5).
- Figures S4–S5: Histograms of distance from SCORE school to geospatial pixel.
- Figures S6–S10: Histograms of predictors, by country.
- Figures S11–S15: Histograms of predictors, by model development set.
- Figures S16–S19: Bar charts of linear and logistic coefficient sizes.
- Figures S20–S23: Bar charts of variable importance in decision tree models.
- Figure S24: ROC curves.
- Figures S25–S28: Performance of prediction models in resampling.
- Table S3: Number of communities included in each training and test set.
- Table S4: Proportion of hotspots in each training and test set.
- Tables S5–S11: Performance metrics for models in the main analysis.
- Tables S12–S13: Coefficients for highlighted models to predict hotspots.
- Table S14: Odds ratios of scaled variables in bivariate logistic regressions.
- Tables S15–S18: Performance metrics for models excluding secondary data.
- Tables S19–S22: Performance metrics for models using 6:2:2 data split.
- Tables S23–S26: Performance metrics for models using corrected prevalence.

SI References

TRIPOD Checklist

Technical Appendix

This appendix provides additional details on the study data, methods, and results.

Background on SCORE randomized trials

The Schistosomiasis Consortium for Operational Research and Evaluation (SCORE) cluster randomized trials compared different strategies for preventive chemotherapy against schistosomiasis (1–4). These studies were performed in five countries: Niger and Mozambique, where SCORE investigated preventive chemotherapy against *S. haematobium*; and Côte d'Ivoire, Kenya, and Tanzania, where SCORE investigated preventive chemotherapy against *S. mansoni*. The study occurred over the period 2011 to 2015. There were six main study arms, with different approaches to preventive chemotherapy specific to each study, the study arms included:

1. Community-wide mass drug administration each year.
2. Community-wide mass drug administration in Year 1 and Year 2, and school-based preventive chemotherapy in Year 3 and Year 4.
3. Community-wide mass drug administration in Year 1 and Year 2, and drug holiday in Year 3 and Year 4.
4. School-based preventive chemotherapy each year.
5. School-based preventive chemotherapy in Year 1 and Year 2, and drug holiday in Year 3 and Year 4.
6. School-based preventive chemotherapy in Year 1 and Year 3, and drug holiday in Year 2 and Year 4.

All communities were surveyed with testing in Year 1 and Year 5, and some were also surveyed in intervening years. A summary of this model variables from the SCORE studies are provided in Table S1. All variables are defined in the first year of the studies, i.e. 2011. Of note, Niger included a semiannual community-wide mass drug administration program.

Table S1: List of model variables from SCORE study to predict whether a community is a *Schistosoma* hotspot.

Variable name	Description	Unit
SAC rounds	Number of rounds of preventive chemotherapy in school-aged children over study period (also includes community rounds in which SAC were also treated)	Rounds of chemotherapy
Community rounds	Number of rounds of preventive chemotherapy in community members over study period	Rounds of chemotherapy
Prevalence	Proportion of tested population with detectable infection	Percentage
Prevalence 5–8	Proportion of tested population 5–8 years old with detectable infection	Percentage
Prevalence 9–12	Proportion of tested population 9–12 years old with detectable infection	Percentage
Mean intensity	Arithmetic mean infection intensity in individuals testing positive, measured in eggs per 10mL urine for *S. haematobium* or eggs per 1g faeces for *S. mansoni*.	mL^{-1} or g^{-1}
Mean intensity 5–8	Arithmetic mean infection intensity in individuals 5–8 years old testing positive	mL^{-1} or g^{-1}
Mean intensity 9–12	Arithmetic mean infection intensity in individuals 9–12 years old testing positive	mL^{-1} or g^{-1}
Dispersion	Dispersion of infection intensity in the population, as measured by the dispersion parameter of a negative binomial fit to intensity	—

SAC: School-aged children

Data Processing

Geospatial survey data

We used health and demographic survey data from the data source, the Demographic and Health Surveys (DHS) (5). We included DHS variables on improved water, improved sanitation, coverage of 3rd dose of DPT vaccine, male education, female education, stunting in under-fives, underweight in under-fives, and under-five mortality. These variables were broadly informed by prior literature, expert opinion, and data availability. We used DHS data in the form of geospatial data sets generated through spatial modelling by the Institute for Health Metrics and Evaluation (IHME), which used Bayesian statistical methods including a range of covariates and spatial structure to predict the value of each variable on a 5x5km pixel grid (6–10). We processed downloaded data with the Python package *Rasterio*, and the pixel grid was assigned longitude-latitude coordinates using the built-in affine transformations in the data. To assign data to study sites based on the latitude and longitude of the local school in each community, we measured the distance between the community coordinates and the centroids of the surrounding pixels and assigned study site to the data from the closest map pixel. All geospatial data is available in online repositories, with associated publications (6–10). Of note, in comparison to the IHME VizHub online visualizer of this data, there were differences in a minority of communities, and our analysis relied on the downloadable data from the publications.

Additional geospatial data

A summary of geospatial variables and their data sources is given in Table S2. All variables are defined in the first year of the SCORE studies, i.e. 2011, with the exception of proximity to fresh water.

Table S2: List of model variables from secondary data sets to predict whether a community is a *Schistosoma* hotspot.

Variable name	Data set	Variable	Resolution	Unit	Description
Vegetation	MODIS (11)	NDVI	250m	ratio	
Proximity to fresh water	CGLS (12)	Water Bodies	60m	m	2020 data
Annual precipitation	WorldClim (13)	Precipitation	2.5 arc-minutes (~5km)	mm	
Minimum temperature	WorldClim (13)	Temperature	30 arc-seconds (~1km)	°C	Minimum of the monthly averages of daily minimum temperature
Population density	GPW (14)	UN-Adjusted Population Density	30 arc-seconds (~1km)	m^{-2}	
Improved sanitation	IHME GHDx (15)	S_IMP	5km	%	Improved sanitation defined as sewer or septic tank, improved latrines (ventilated or unventilated), or compost toilets (8)
Improved water	IHME GHDx (15)	W_IMP	5km	%	Improved water defined as piped on or off premises, from a protected spring or well, rainwater, bottled water, or tanker truck (8)
3rd dose of DPT vaccine	IHME GHDx (16)	DPT3 Coverage	5km	%	DPT: diphtheria, pertussis, tetanus. Used as proxy for healthcare access
Education	IHME GHDx (17)	EDU 15–49 Male/Female Mean	5km	years	Two separate variables for male and female years of education
Underweight	IHME GHDx (18)	Underweight Prevalence Under 5	5km	%	Underweight defined as weight-for-age z-score more than two standard deviations below mean in WHO Child Growth Standards
Stunting	IHME GHDx (18)	Stunting Prevalence Under 5	5km	%	Stunting is defined as height-for-age z-score more than two standard

					deviations below mean in WHO Child Growth Standards
Under-5 mortality	IHME GHDx (18)	Under-5 Mortality Probability of Death	5km	—	
Relative wealth	HDX (19)	Relative Wealth Index	2.4km	—	Defined on a within country scale

Note: IHME GHDx related upon DHS data.

Missing data

For SCORE study data, we excluded any community with missing baseline prevalence data available in the 5–8 and 9–12 year old age groups (see Figure S1).

For geospatial data sets, data were defined as missing when no estimate was available for the corresponding pixel to the study community. This often occurred when a study site was in a sparsely populated area or near a body of water. In these scenarios, we measured the distance between the study site coordinates and the centroids of the surrounding pixels and assigned to the community to the closest map pixel. Histograms for the distance between each study site and the closest pixel centroid for each data set are given in Figures S4–S5.

Statistical Analysis

Hyperparameter selection

During model development, the model hyperparameters were selected through cross-validation using the training data set. For linear and logistic regression models with elastic net we selected the hyperparameters of regularization strength and l1:l2 ratio. For random forest models we selected the hyperparameters of bootstrap size and variable subset size (number of variables available to each tree in the random forest). For boosted trees models we selected the hyperparameters of tree depth and learning rate. For support vector machines we selected the hyperparameters of regularization strength and length scale of the gaussian radial basis function kernel. For multilayer perceptrons we selected the hyperparameters of regularization strength and hidden layer size. We used a single hidden layer. All other model development parameters were set to their defaults in Scikit-learn version 1.1.1 (20) or XGBoost Python package version 1.7.6 (21).

Calculating predictive value

We computed negative predictive value (NPV) and positive predictive value (PPV) for this study, which is dependent on a baseline prevalence for hotspot. If the sensitivity of a model is *s* and the specificity is s*p*, then for hotspot prevalence *x*, then:

$$\text{PPV} = sx/(sx + (1 - sp)(1 - x))$$

$$\text{NPV} = sp(1 - x)/((1 - s)x + sp(1 - x))$$

Generating Figure 2

The two rightmost columns in figure 2 give the mean balanced accuracy for validation approaches which include multiple test sets for *S. mansoni*. In this case, we took the mean balanced accuracy in the between countries Côte d'Ivoire, Kenya, and Tanzania test sets, and the mean balanced accuracy in the within country Kenya and Tanzania test sets.

Additional details

All analyses were automated and data were randomly assigned to the training and test sets. We did not perform any blinding during this analysis given this data split was automated. The study design was specified prior to generation of results, with adjustments made after peer review.

Sensitivity analysis

Correcting for imperfect diagnostics

To evaluate the role of imperfect diagnostic tools for *Schistosoma* infection, we performed an alternative analysis where we adjusted prevalence estimates for imperfect diagnostic sensitivity using literature values. For *S. mansoni* we used an analysis from Bärenbold et al. 2021 (22), which provides a formula for estimating true prevalence from prevalence measured using Kato-Katz fecal microscopy, taking into account the number of fecal samples taken, and the number of slides examined from each sample. For *S. haematobium*, we used an analysis from Midzi et al. 2020 (23), which uses a latent class analysis to estimate the true prevalence corresponding to several values of prevalence as calculated using urine filtration. The relationship between these quantities was roughly linear, and we performed a linear regression to relate the measured prevalence to the true prevalence, with intercept 0.075 and slope 1.54. For urine filtration prevalences greater than 1/1.54 = 0.65, we assumed a true prevalence of 100%.

Supplemental Tables and Figures

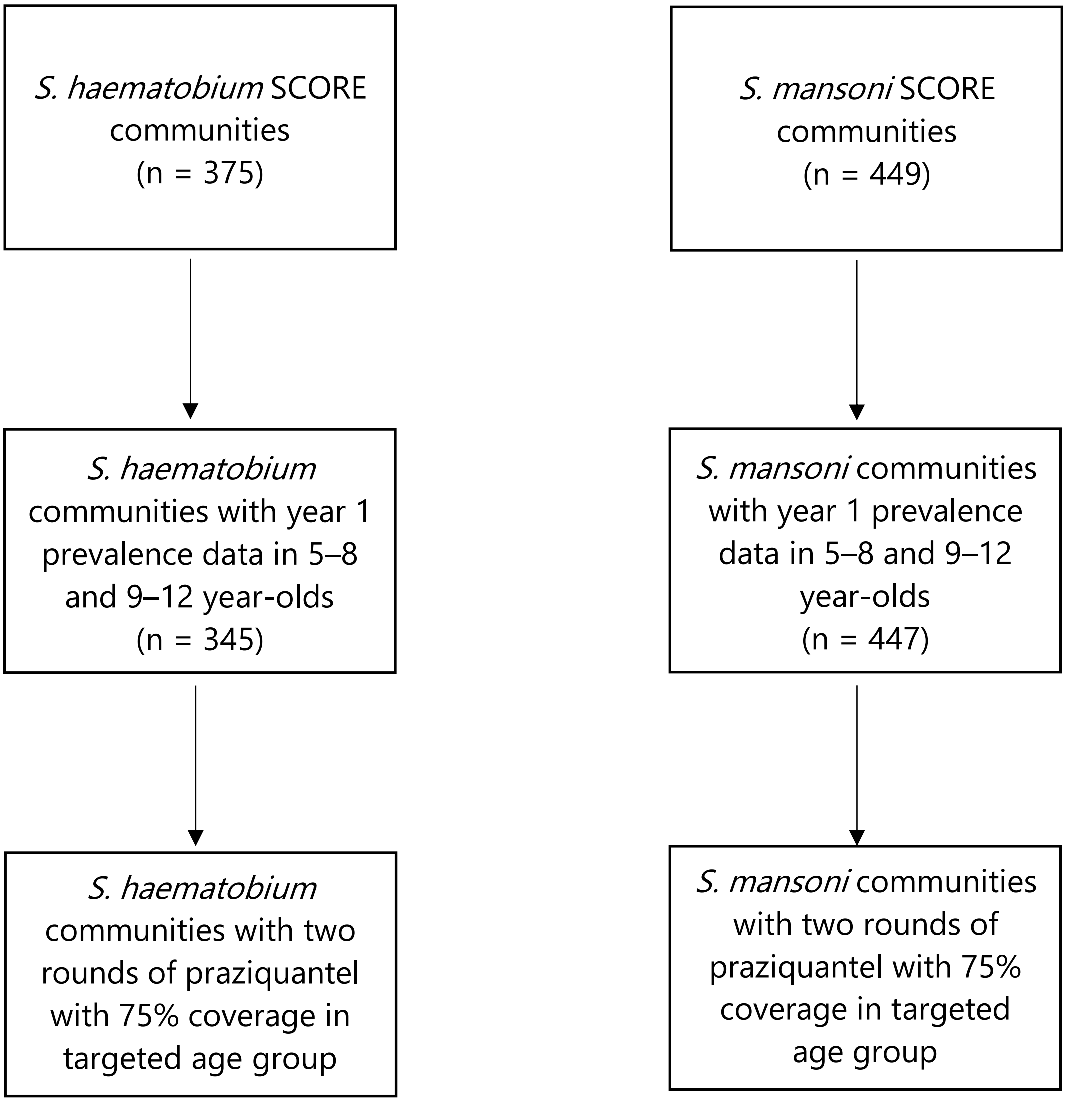

Figure S1: Flow charts of data exclusions.

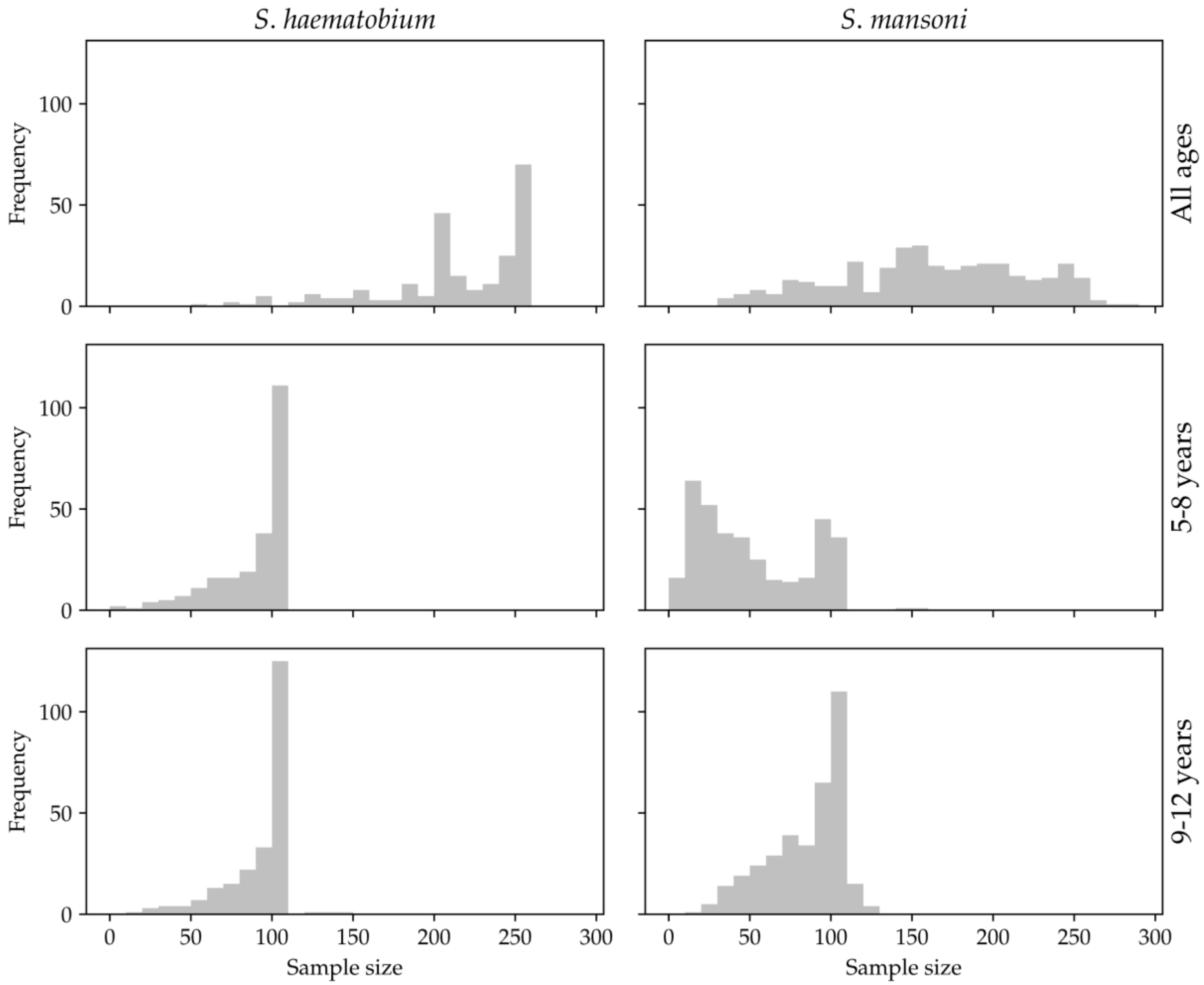

Figure S2: Histograms of number of individuals tested per community by age group and species in year 1 of the SCORE study.

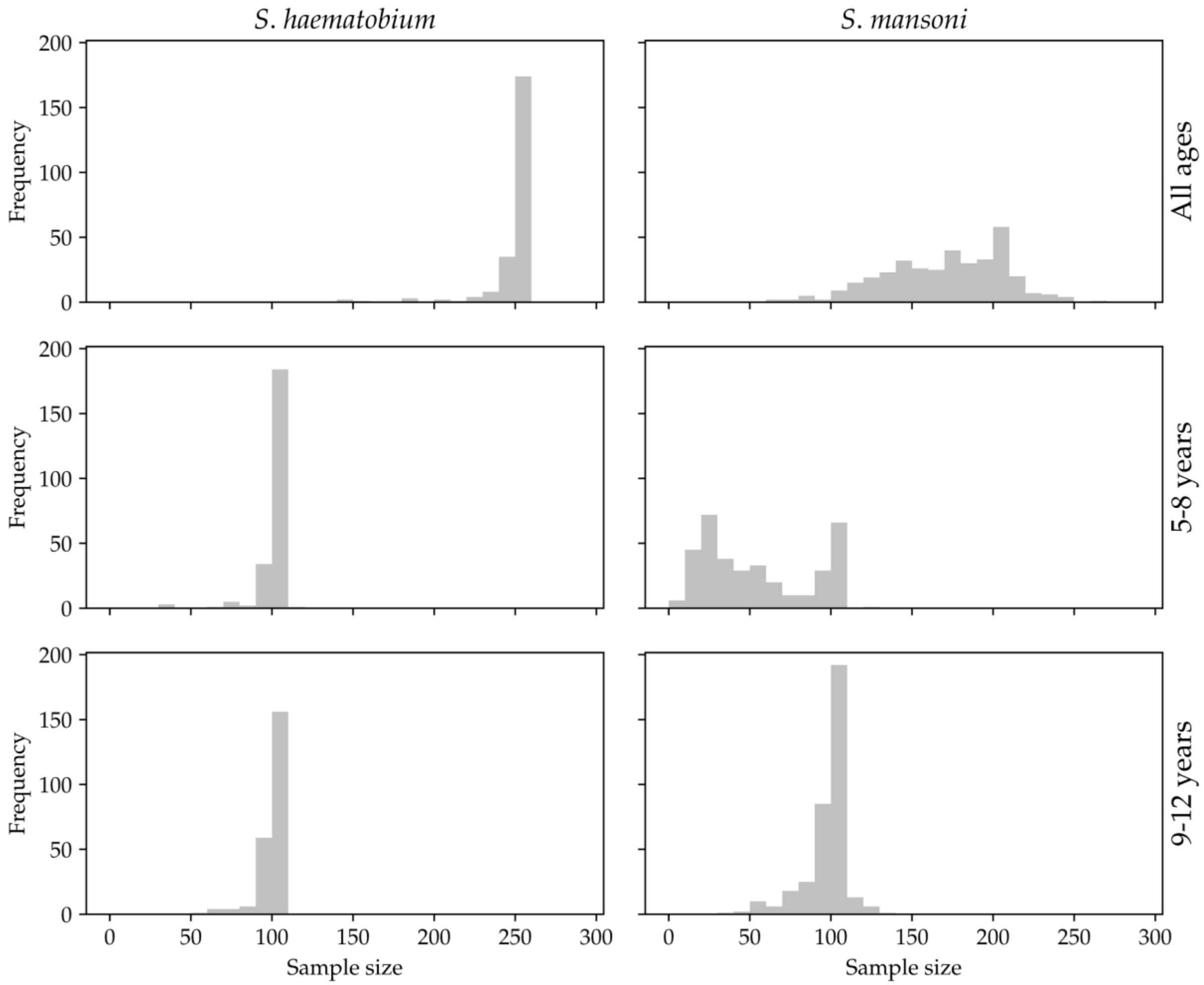

Figure S3: Histograms of number of individuals tested per community by age group and species in year 5 of the SCORE study.

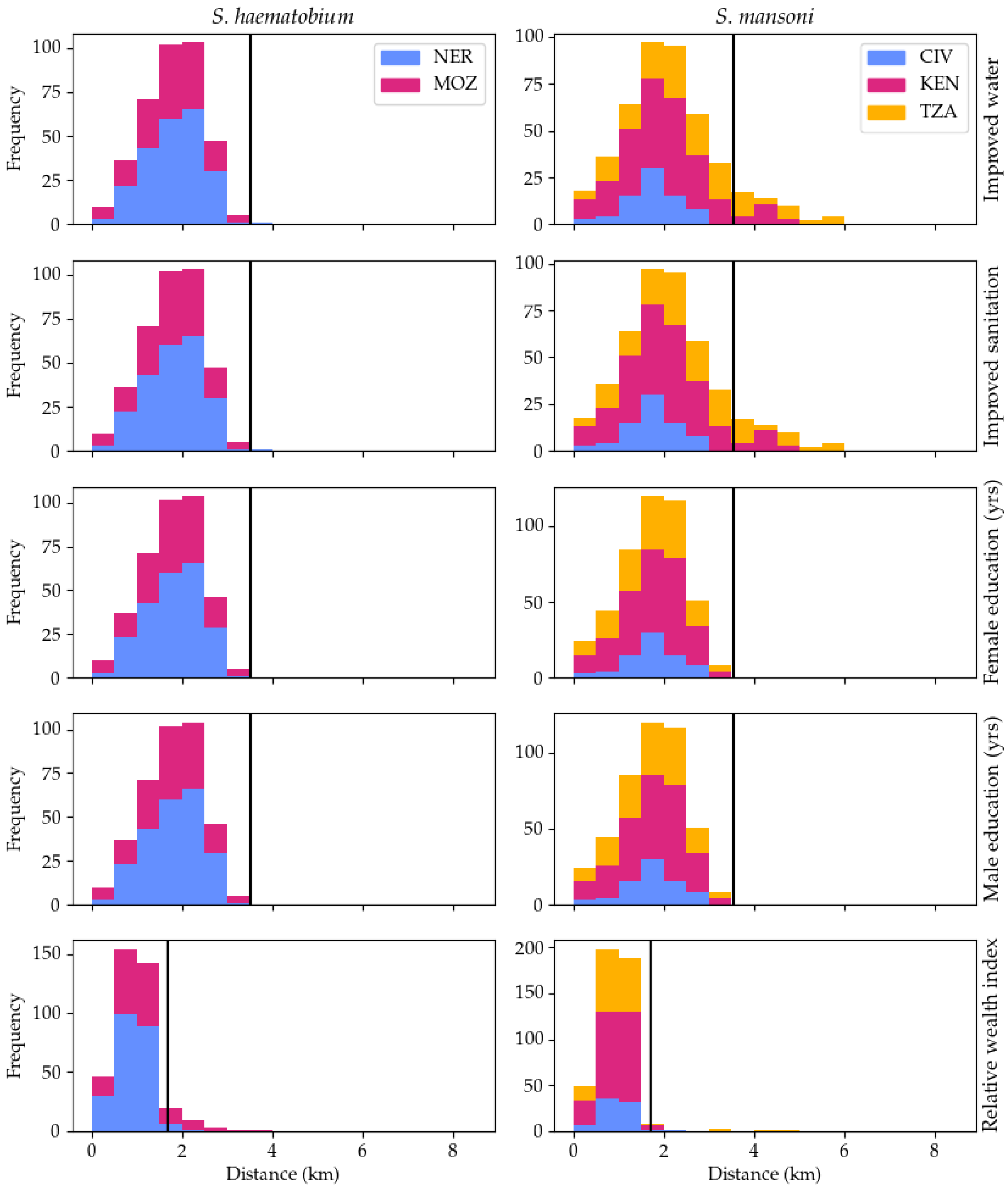

Figure S4: Histograms of distance between local school coordinates and the closest pixel centroid in the corresponding geospatial data set, for survey-based WASH, education, and wealth variables. Vertical black lines indicate the length of half a diagonal of a pixel in the geospatial data set, i.e. the distance from the corner of a pixel to its centroid. If the data is from the same pixel as the school coordinates then the distance will be smaller than or equal to this value. Country abbreviations: MOZ—Mozambique, NER—Niger, CIV—Côte d'Ivoire, KEN—Kenya, TZA—Tanzania.

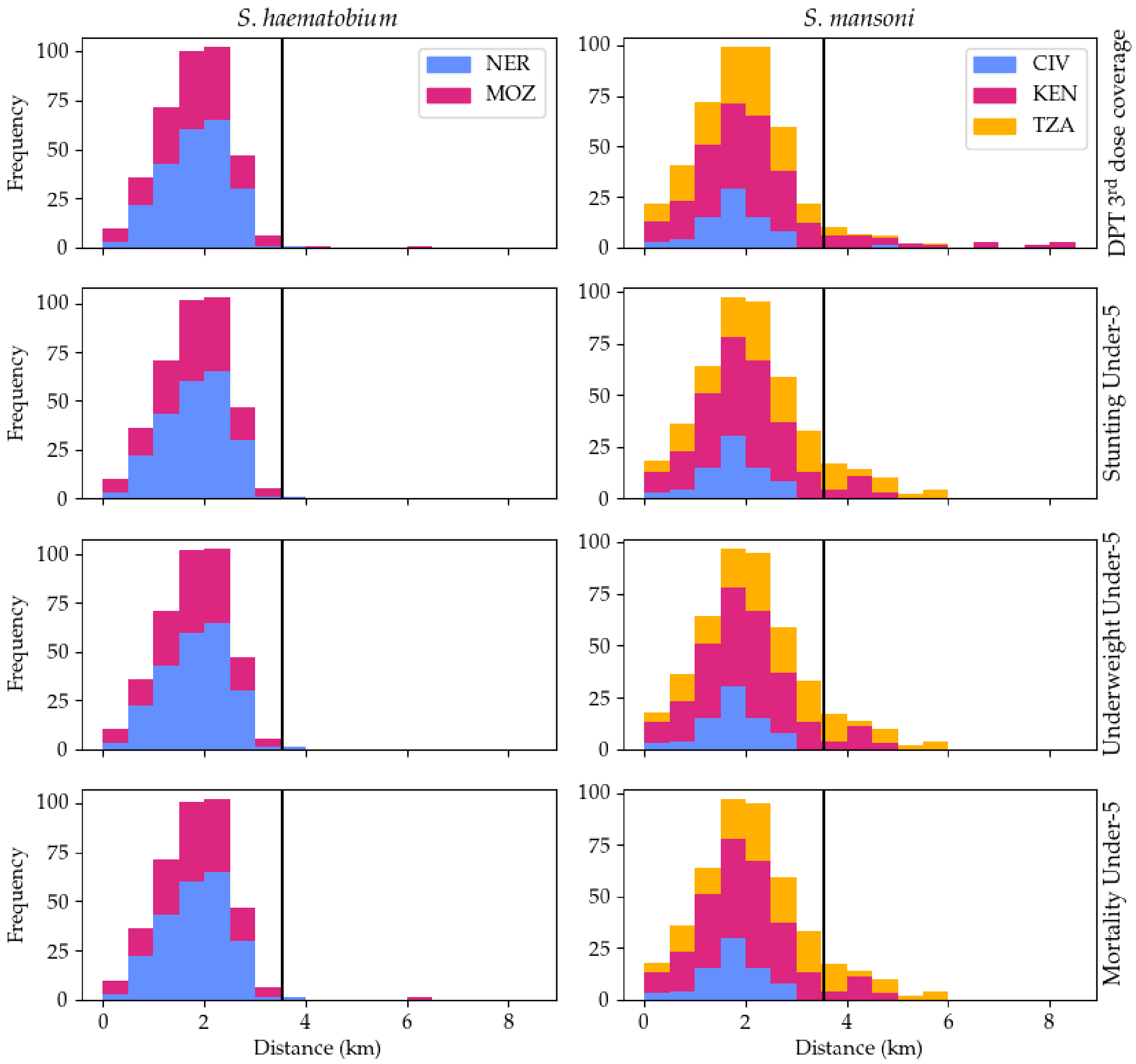

Figure S5: Histograms of distance between local school coordinates and the closest pixel centroid in the corresponding geospatial data set, for health survey-based variables. Vertical black lines indicate the length of half a diagonal of a pixel in the geospatial data set, i.e. the distance from the corner of a pixel to its centroid. Country abbreviations: MOZ—Mozambique, NER—Niger, CIV—Côte d'Ivoire, KEN—Kenya, TZA—Tanzania.

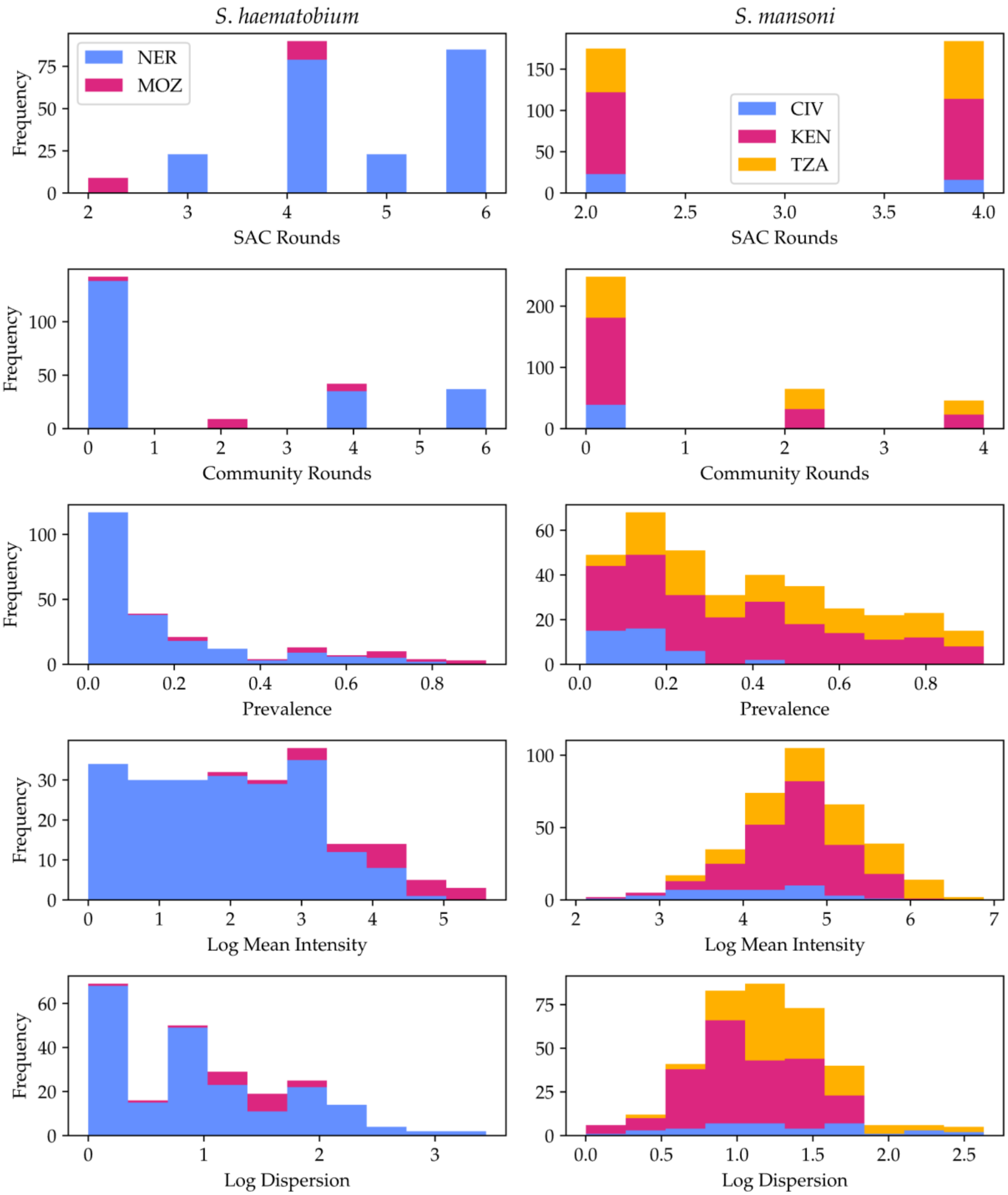

Figure S6: Histograms of non-age-structured variables based on SCORE data for included communities in *S. haematobium* and *S. mansoni* SCORE countries, colored by country. Country abbreviations: MOZ—Mozambique, NER—Niger, CIV—Côte d'Ivoire, KEN—Kenya, TZA—Tanzania.

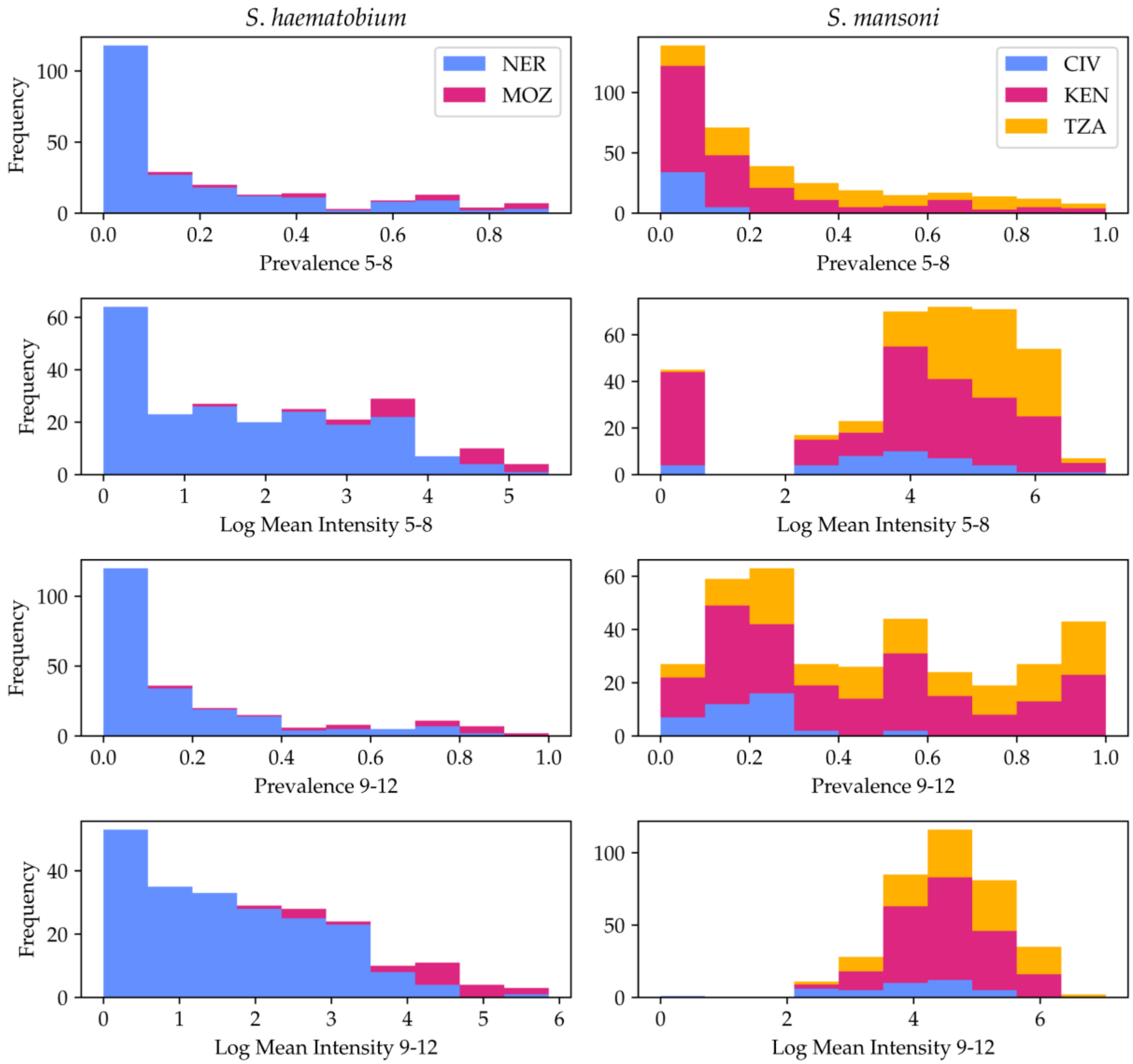

Figure S7: Histograms of age-structured variables based on SCORE data for included communities in *S. haematobium* and *S. mansoni* SCORE countries, colored by country. Country abbreviations: MOZ—Mozambique, NER—Niger, CIV—Côte d'Ivoire, KEN—Kenya, TZA—Tanzania.

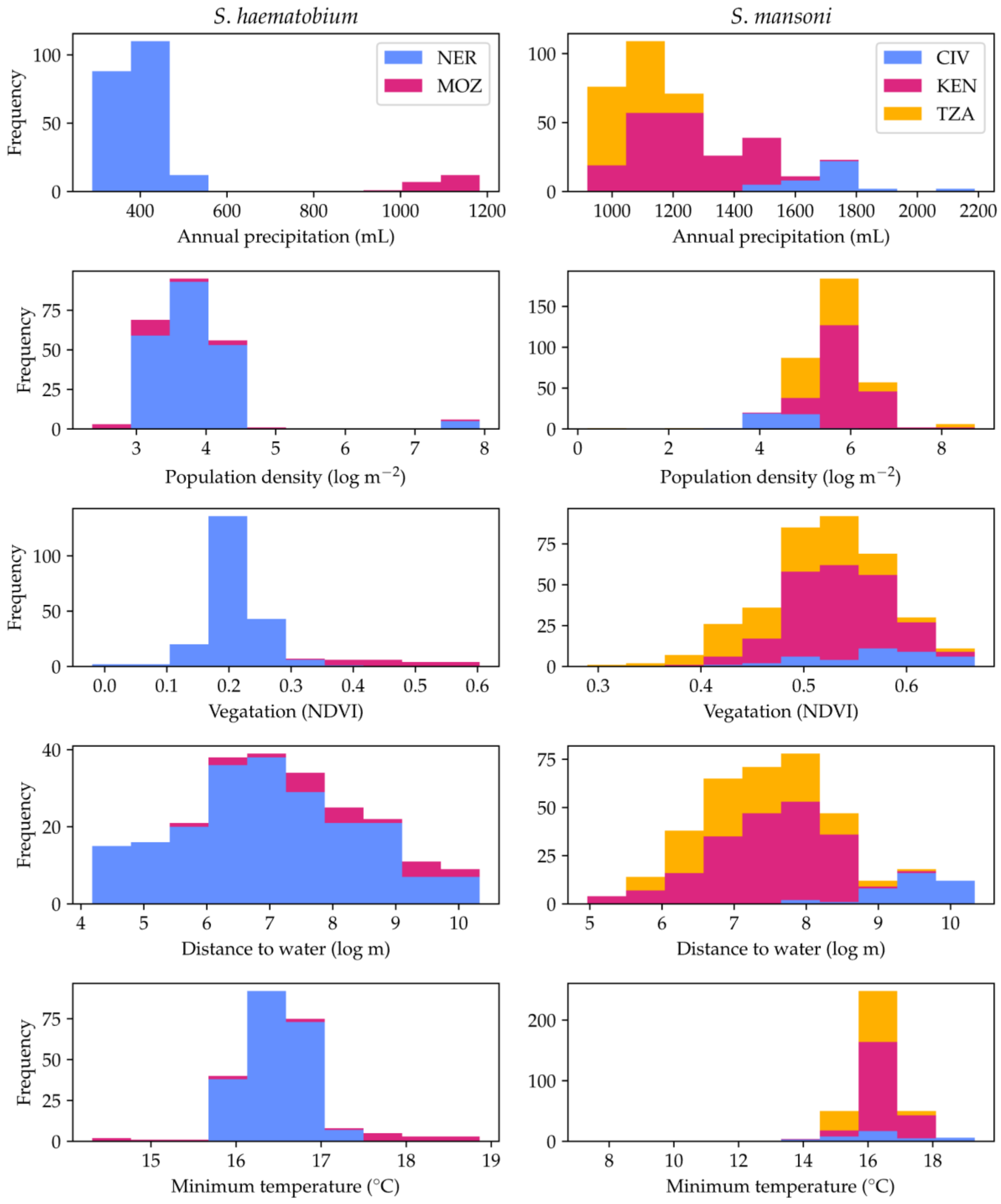

Figure S8: Histograms of remote sensing variables for communities in *S. haematobium* and *S. mansoni* SCORE countries, colored by country. Country abbreviations: MOZ—Mozambique, NER—Niger, CIV—Côte d'Ivoire, KEN—Kenya, TZA—Tanzania.

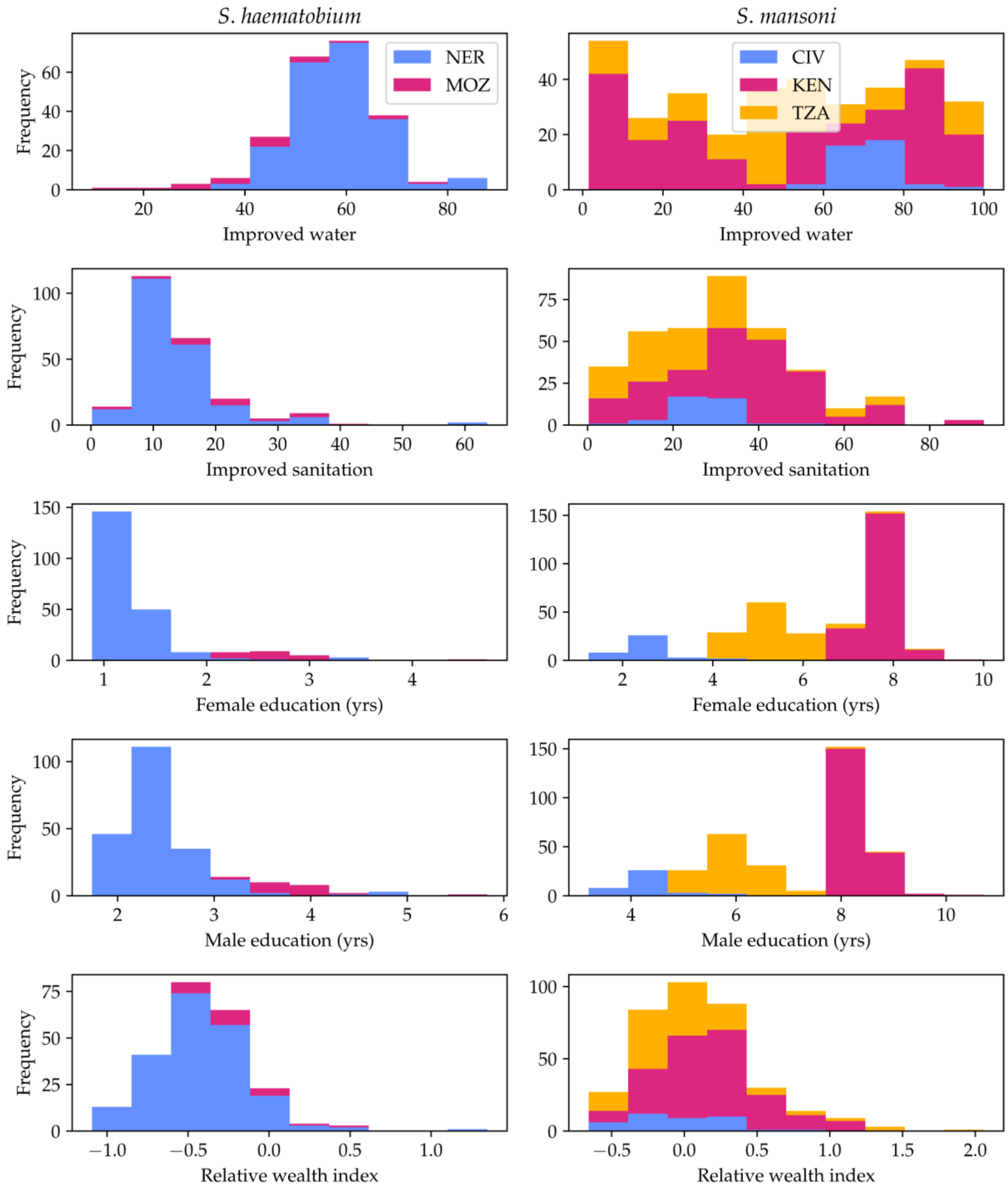

Figure S9: Histograms of WASH, education, and wealth survey-based variables for communities in *S. haematobium* and *S. mansoni* SCORE countries, colored by country. Country abbreviations: MOZ—Mozambique, NER—Niger, CIV—Côte d'Ivoire, KEN—Kenya, TZA—Tanzania. Relative wealth index is relative to others in same country. Improved water and sanitation expressed as percentage.

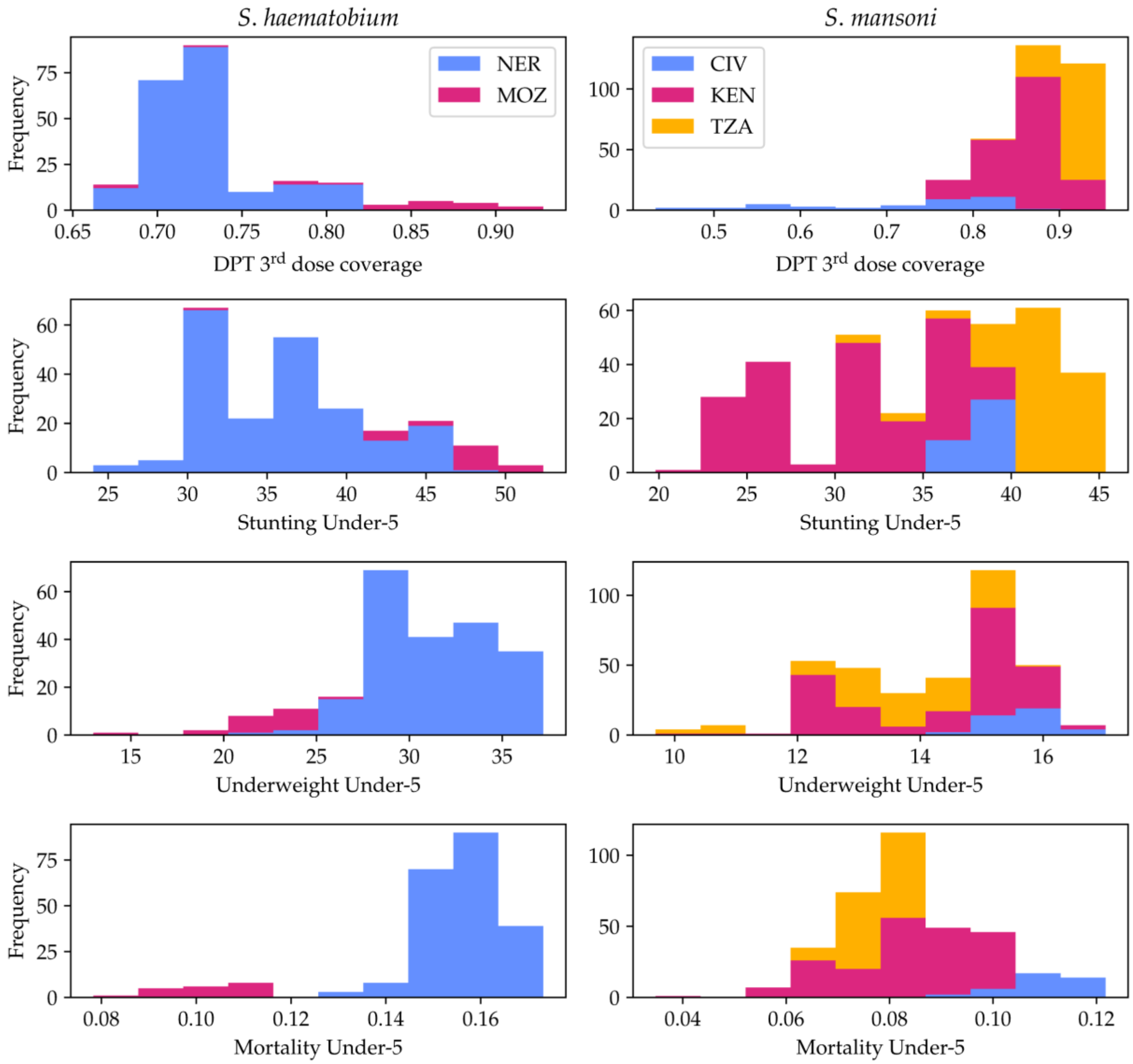

Figure S10: Histograms of health survey-based variables for communities in *S. haematobium* and *S. mansoni* SCORE countries, colored by country. Country abbreviations: MOZ—Mozambique, NER—Niger, CIV—Côte d'Ivoire, KEN—Kenya, TZA—Tanzania. Stunting and underweight expressed as prevalence (%), while mortality is given as probability of death by age 5.

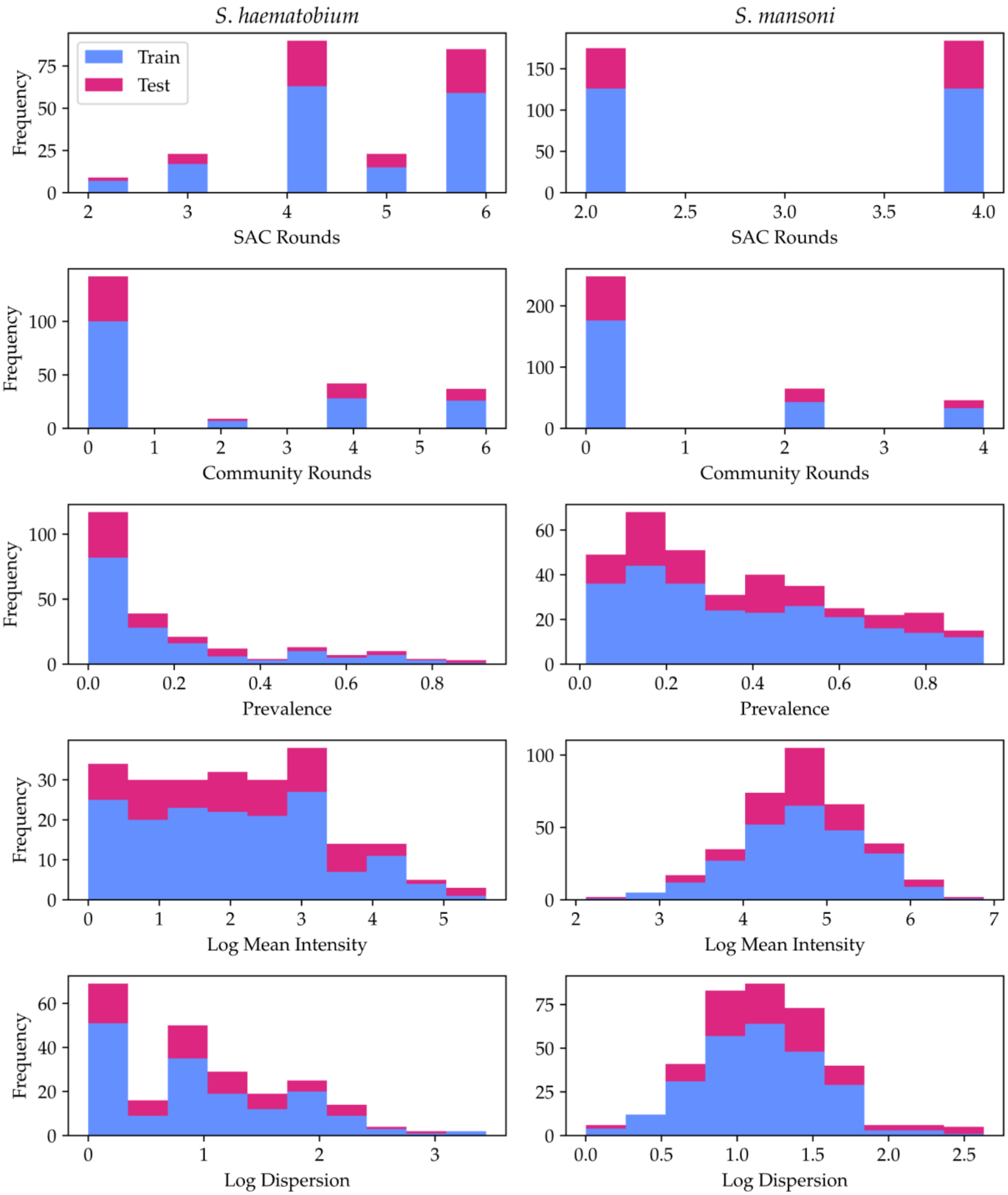

Figure S11: Histograms of non-age-structured variables based on SCORE data for included communities in *S. haematobium* and *S. mansoni* SCORE countries, colored by inclusion in the combined countries train set (for model development), or the combined countries test set.

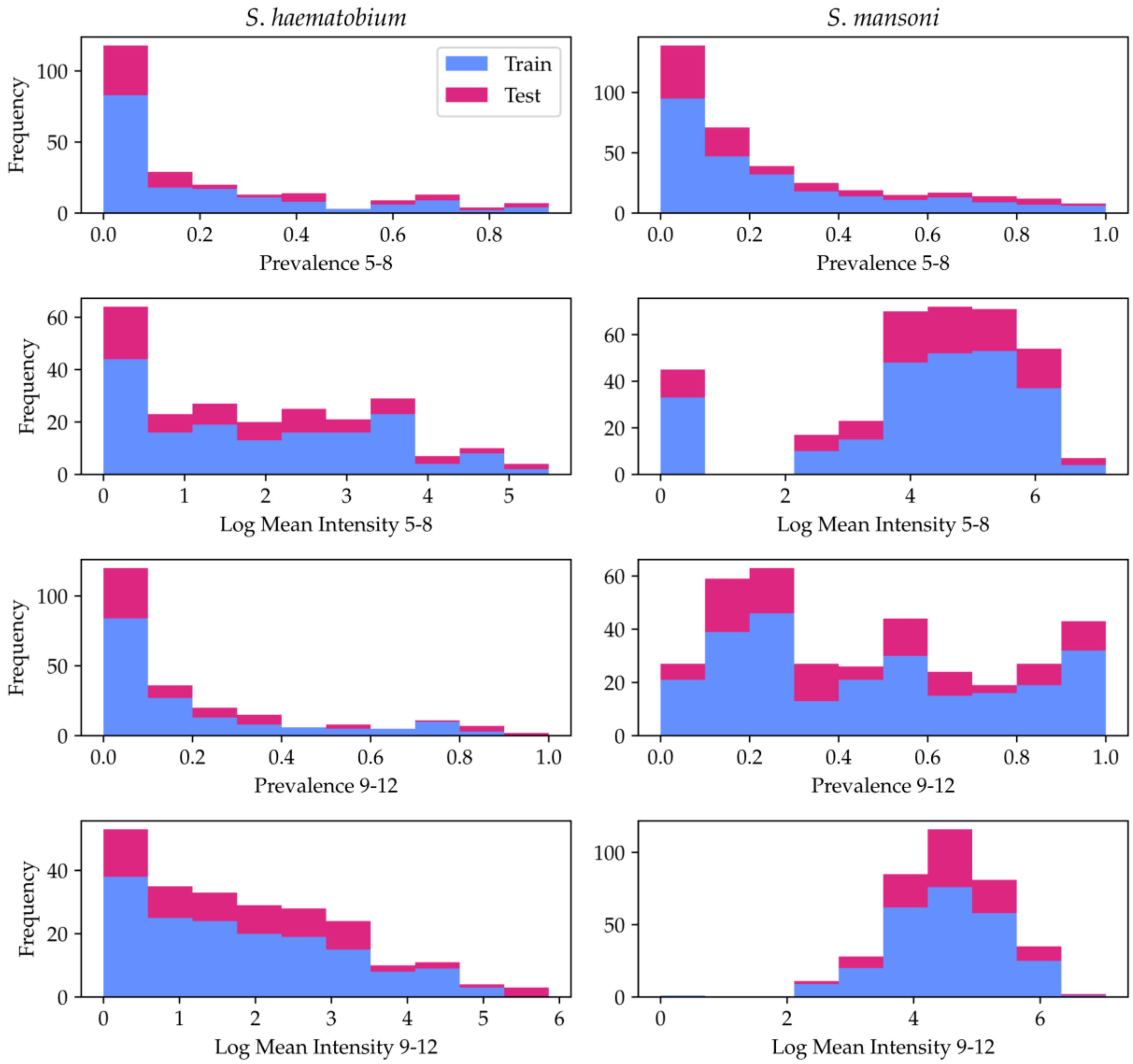

Figure S12: Histograms of age-structured variables based on SCORE data for included communities in *S. haematobium* and *S. mansoni* SCORE countries, colored by inclusion in the combined countries train set (for model development), or the combined countries test set.

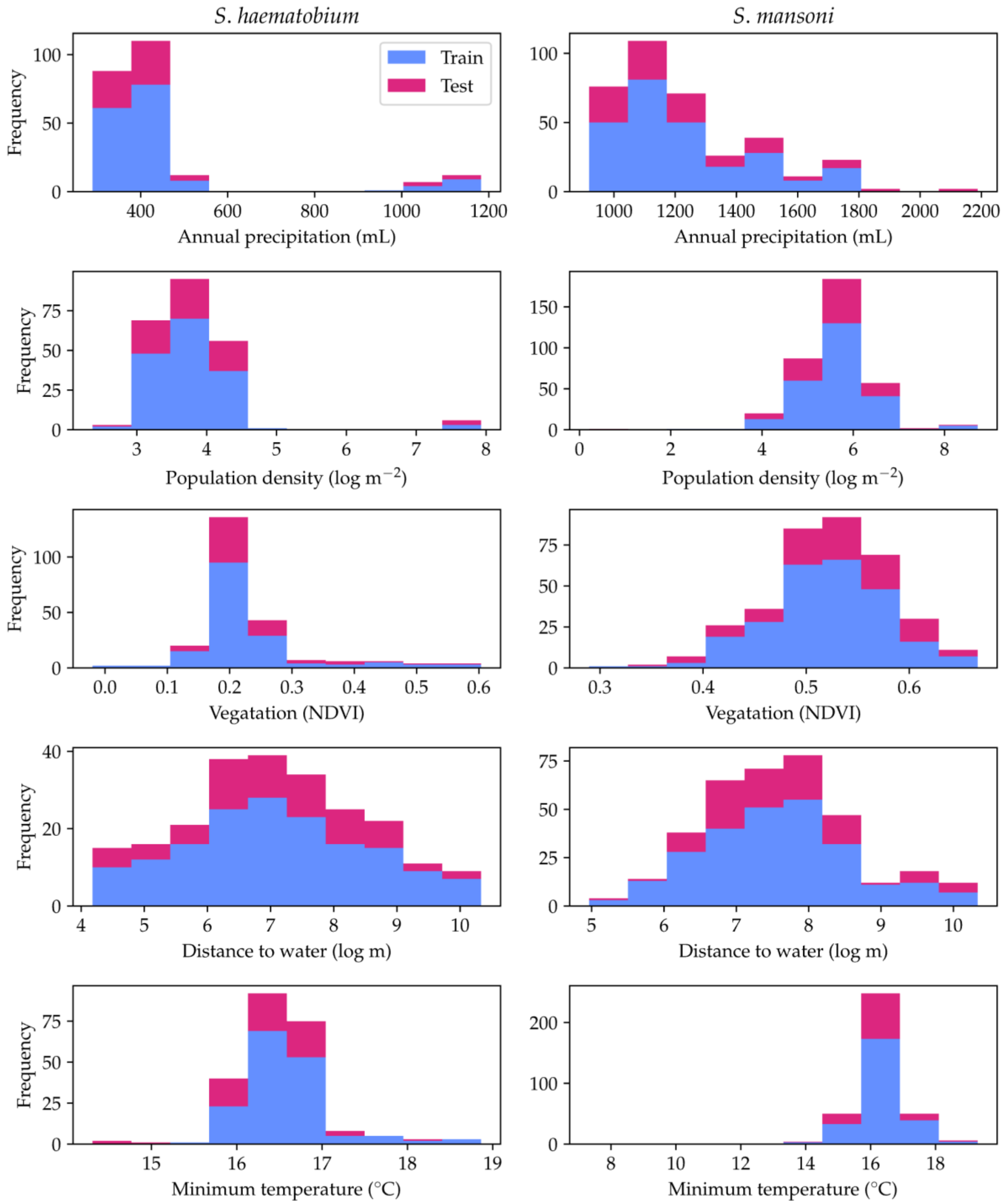

Figure S13: Histograms of remote sensing variables for communities in *S. haematobium* and *S. mansoni* SCORE countries, colored by inclusion in the combined countries train set (for model development), or the combined countries test set.

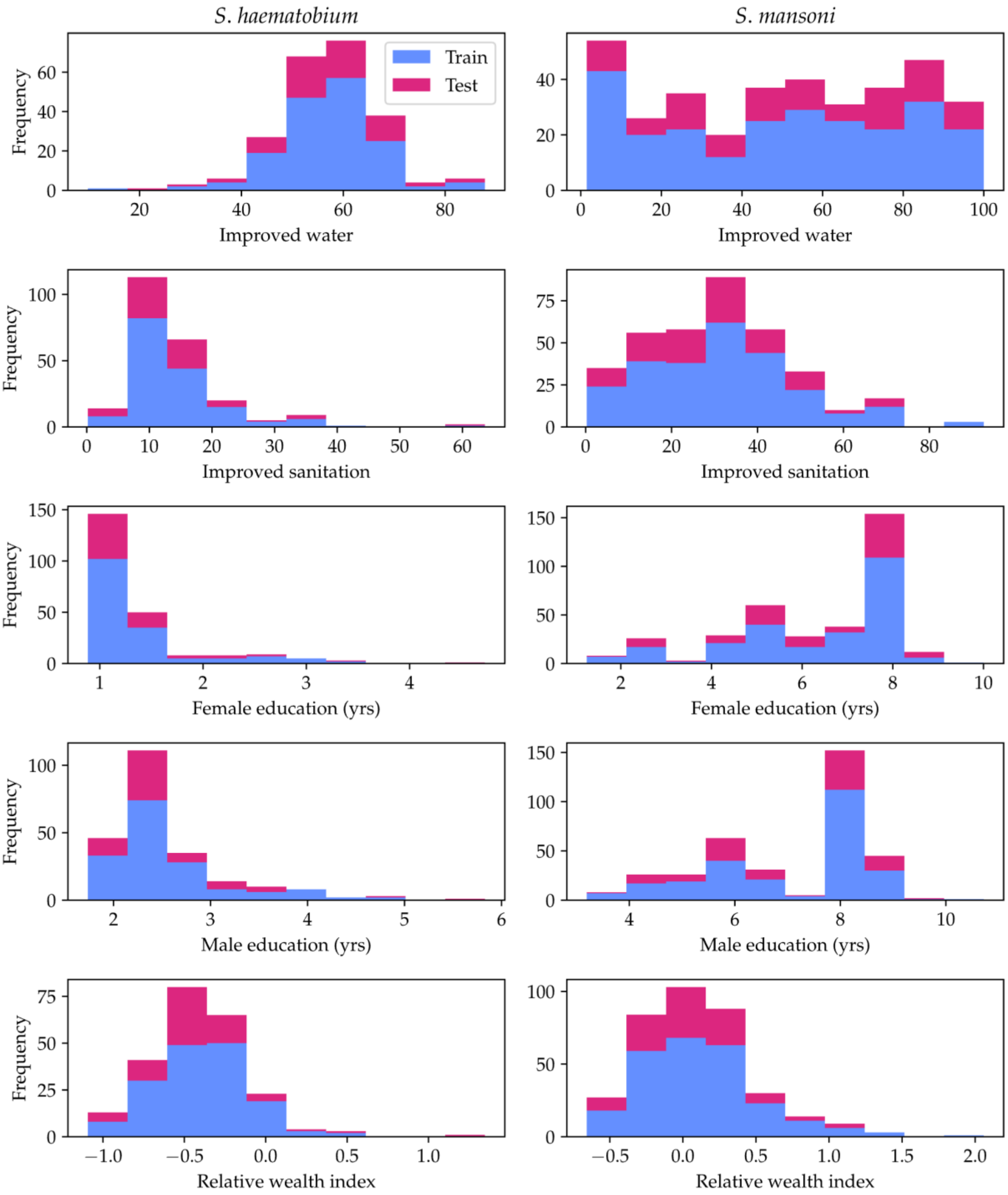

Figure S14: Histograms of WASH, education, and wealth survey-based variables for communities in *S. haematobium* and *S. mansoni* SCORE countries, colored by inclusion in the combined countries train set (for model development), or the combined countries test set.

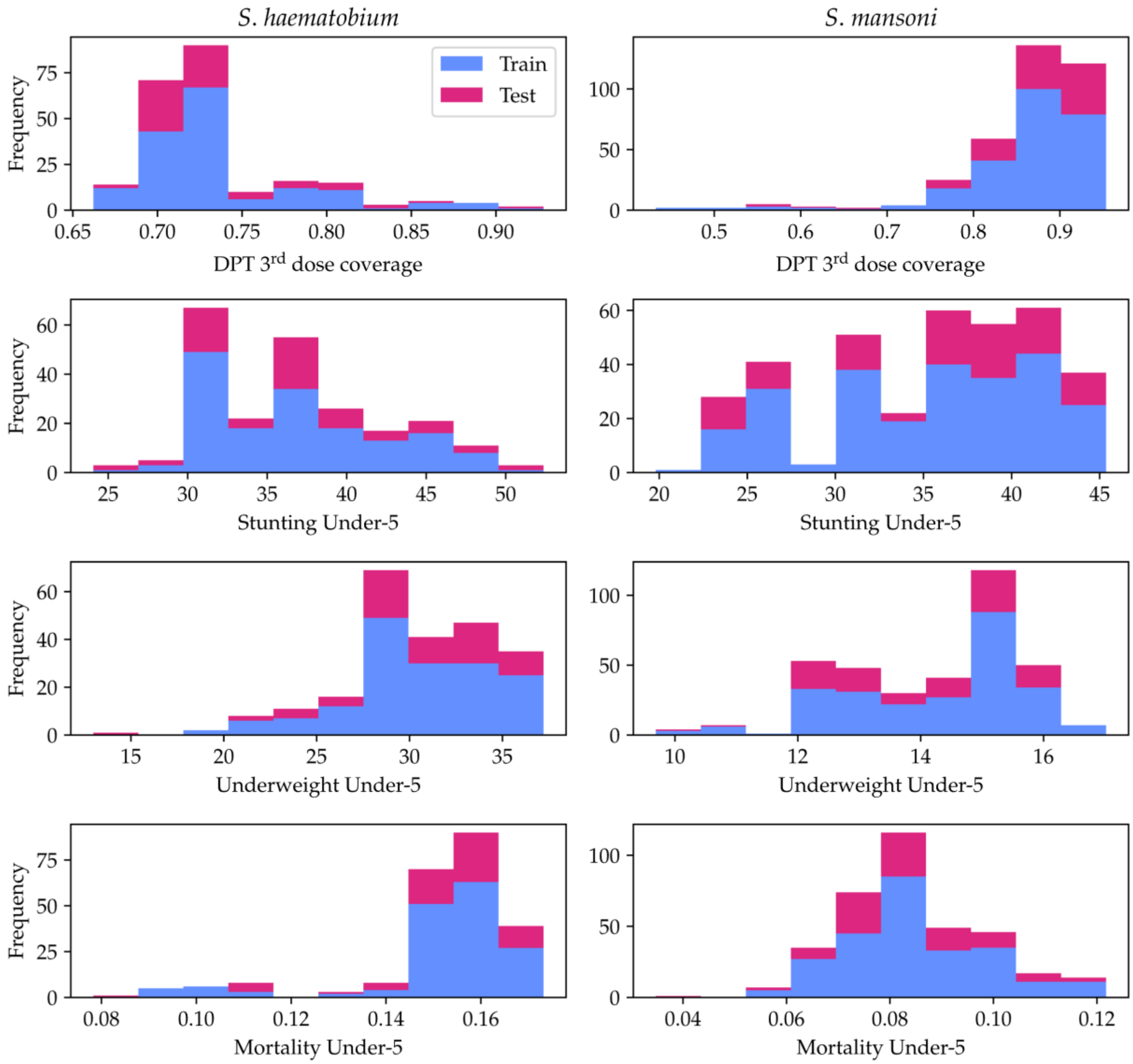

Figure S15: Histograms of health survey-based variables for communities in *S. haematobium* and *S. mansoni* SCORE countries, colored by inclusion in the combined countries train set (for model development), or the combined countries test set.

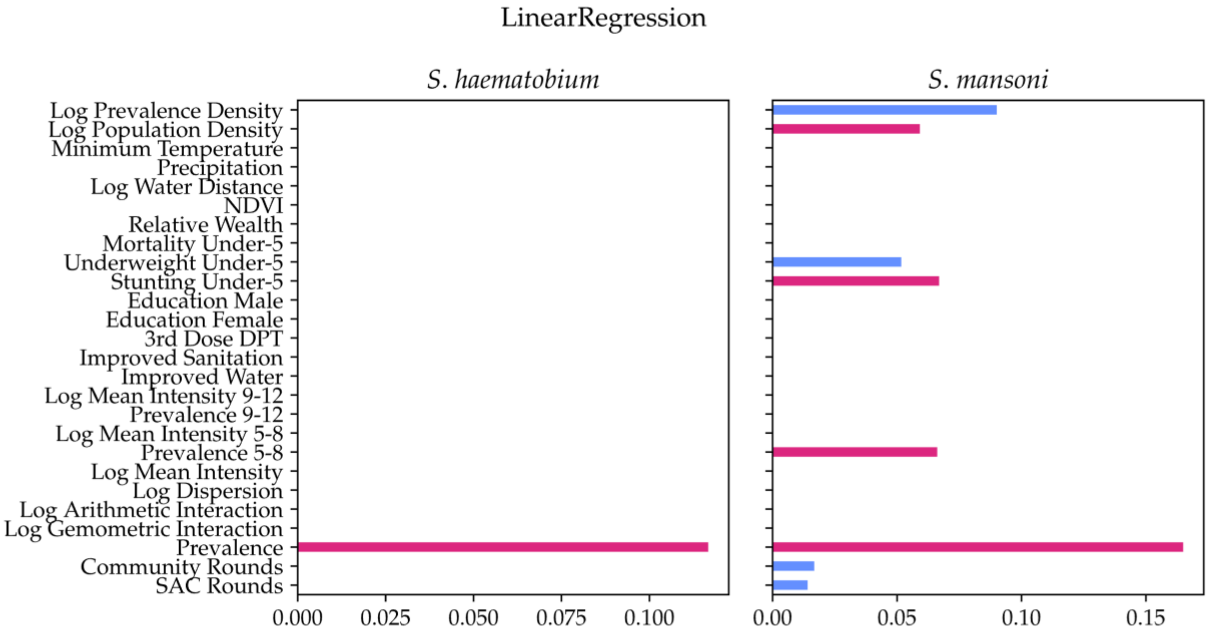

Figure S16: Coefficient sizes of the linear regression model with forward variable selection for predicting year 5 prevalence in the combined countries training set, for each species of *Schistosoma*. Absolute value of the coefficients is plotted—positive coefficients are in red, and negative coefficients in blue.

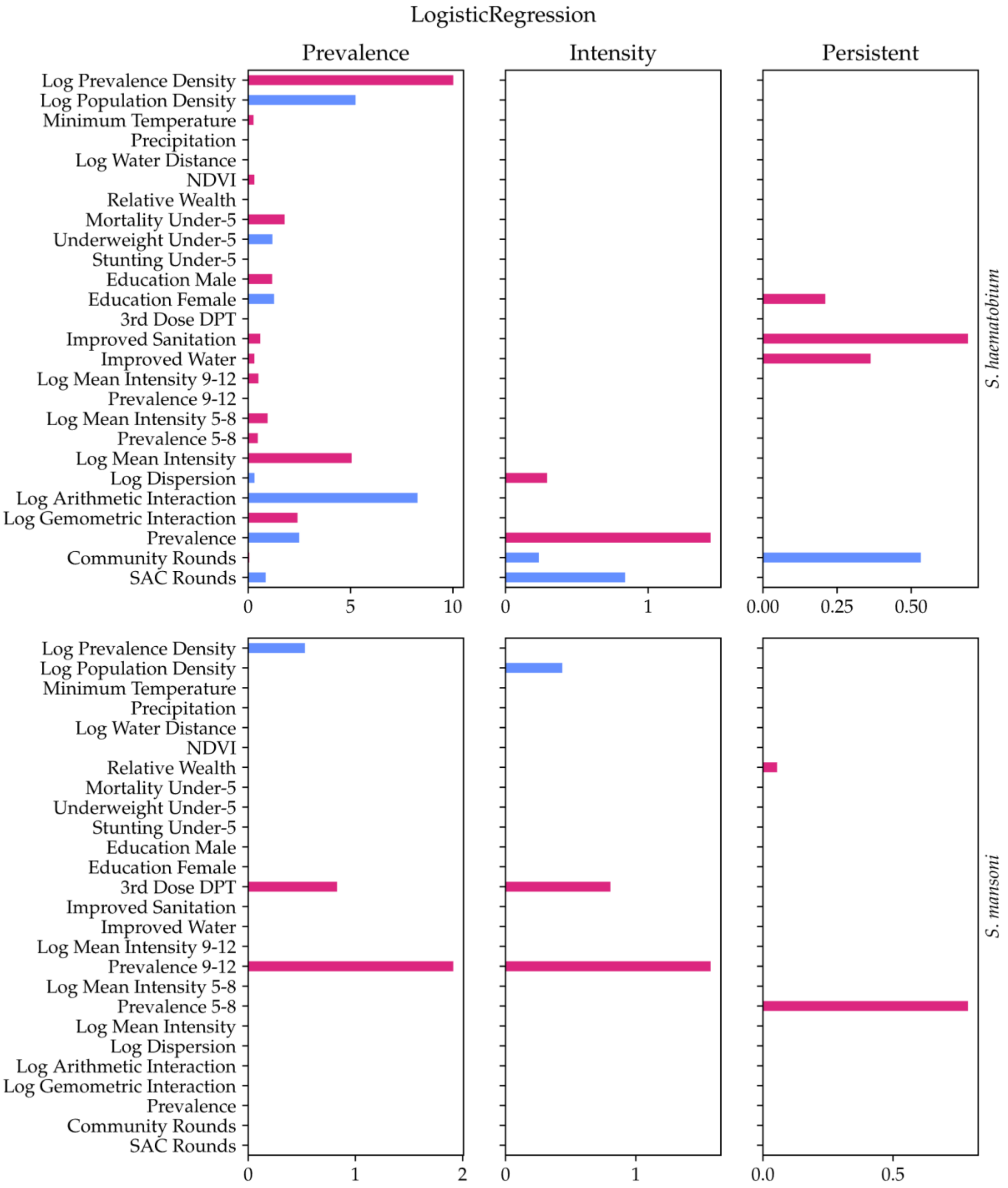

Figure S17: Coefficient sizes of the logistic regression model with forward variable selection using the combined country training set, by hotspot definition and *Schistosoma* species. Absolute value of the coefficients is plotted—positive coefficients are in red, and negative coefficients in blue.

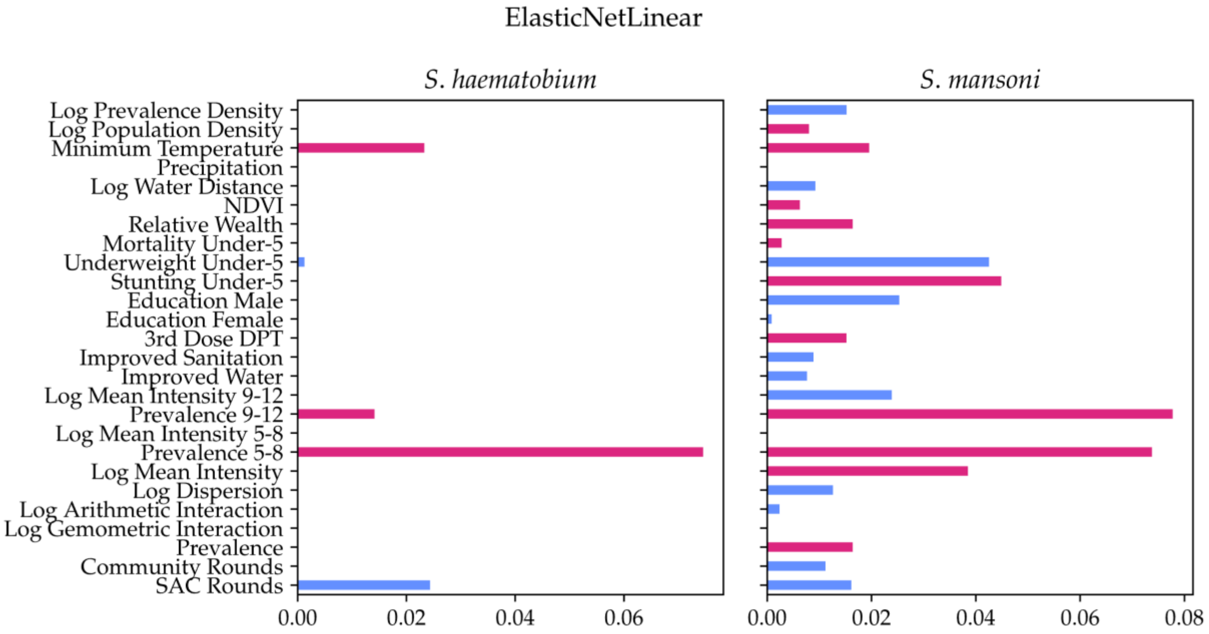

Figure S18: Coefficient sizes of the linear regression model with elastic net regularization for predicting year 5 prevalence in the combined countries training set, for each species of *Schistosoma*. Absolute value of the coefficients is plotted—positive coefficients are in red, and negative coefficients in blue.

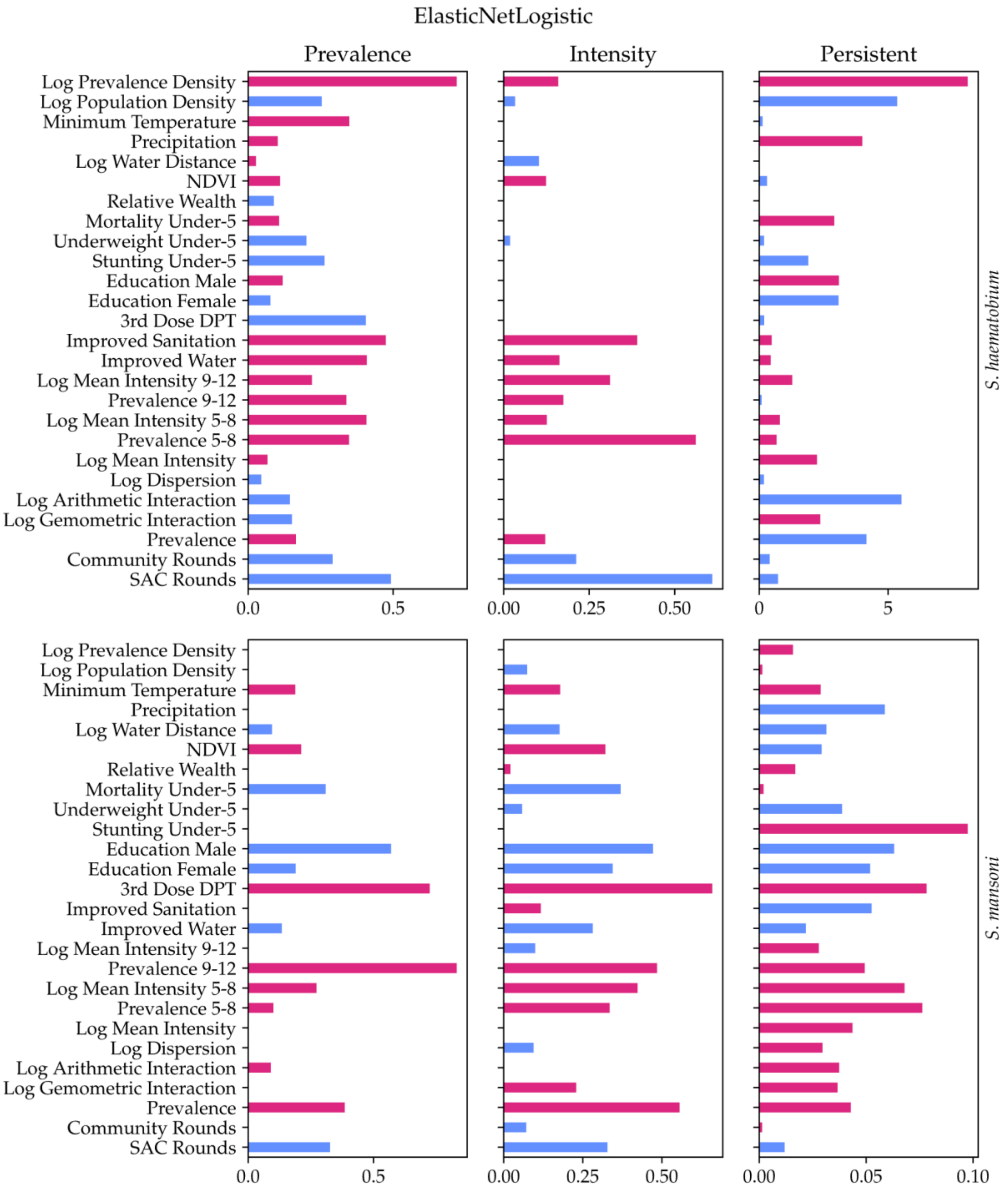

Figure S19: Coefficient sizes of the logistic regression model with elastic net regularization using the combined country training set, by hotspot definition and *Schistosoma* species. Absolute value of the coefficients is plotted—positive coefficients are in red, and negative coefficients in blue. Overall magnitude of coefficient sizes for persistent hotspot definition were highly divergent between the two species.

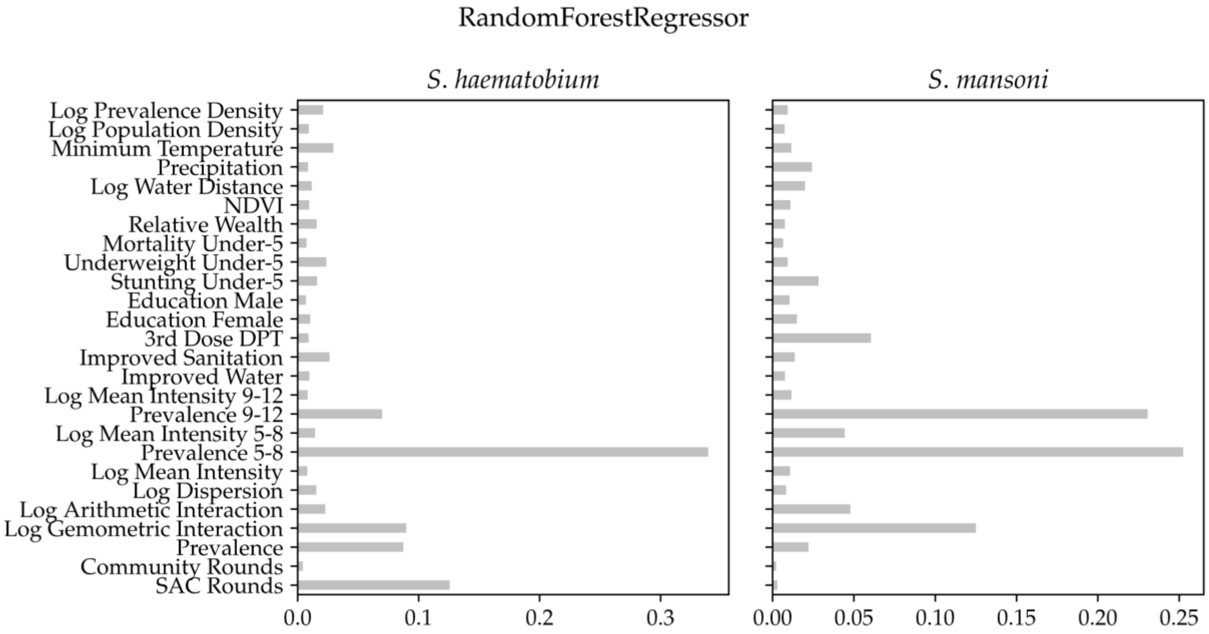

Figure S20: Impurity-based (Gini) importance of each variable in the random forest regression models using the combined country training data and the prevalence hotspot definition.

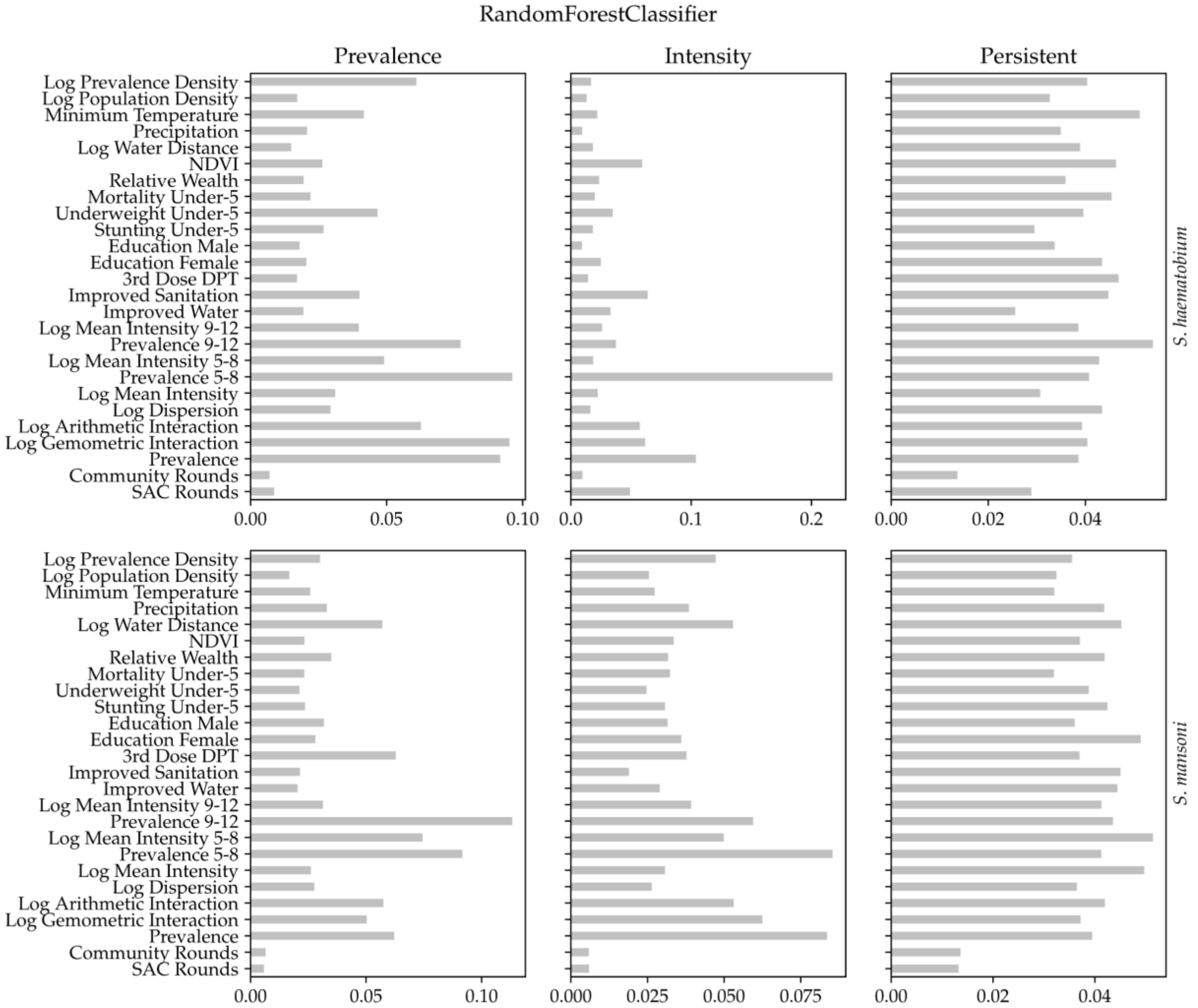

Figure S21: Impurity-based (Gini) importance of each variable in the random forest classification models using the combined country training data.

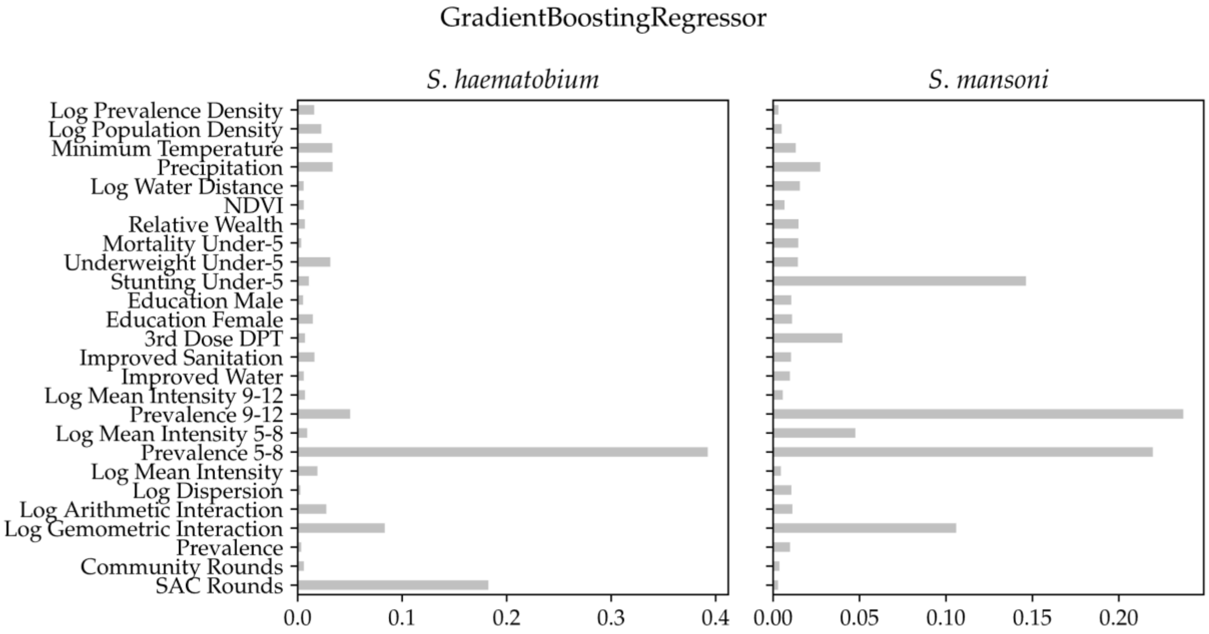

Figure S22: Impurity-based (Gini) importance of each variable in the boosted trees regression models using the combined country training data and the prevalence hotspot definition.

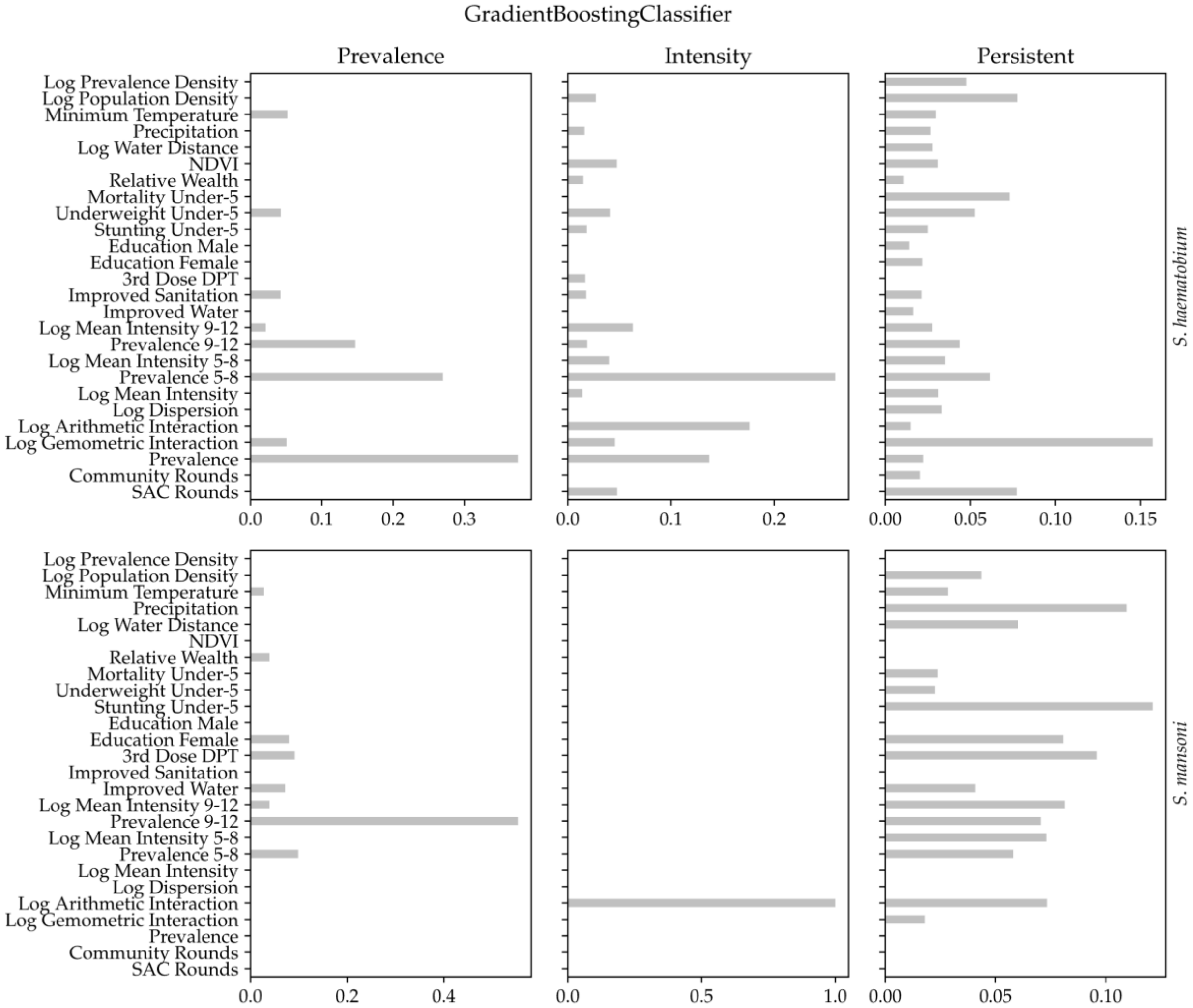

Figure S23: Impurity-based (Gini) importance of each variable in the boosted trees classification models using the combined country training data.

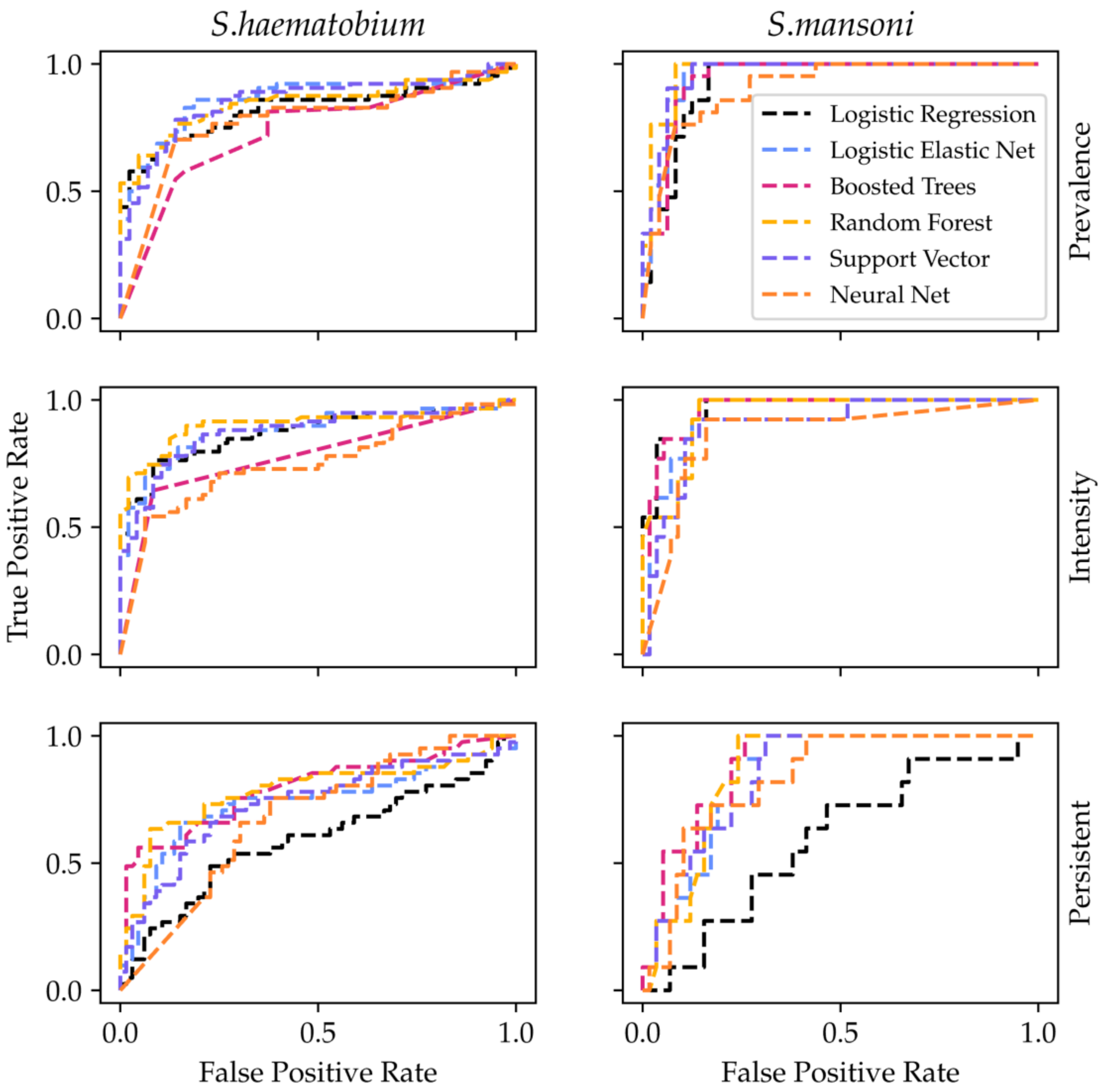

Figure S24: Receiver operator characteristic (ROC) curves for classification models in the combined countries test set, by species and hotspot definition. The hotspot definitions are, from top to bottom: 1) prevalence of *Schistosoma* infection in year 5 >10%; 2) prevalence of infection in year 5 >10% and prevalence of moderate to heavy intensity infection in year 5 >1%; and 3) prevalence of infection in year 5 >10% and relative reduction in infection prevalence from year 1 to year 5 <35%. Note, sensitivity is synonymous with true positive rate and specificity is equivalent to 1 minus the false positive rate.

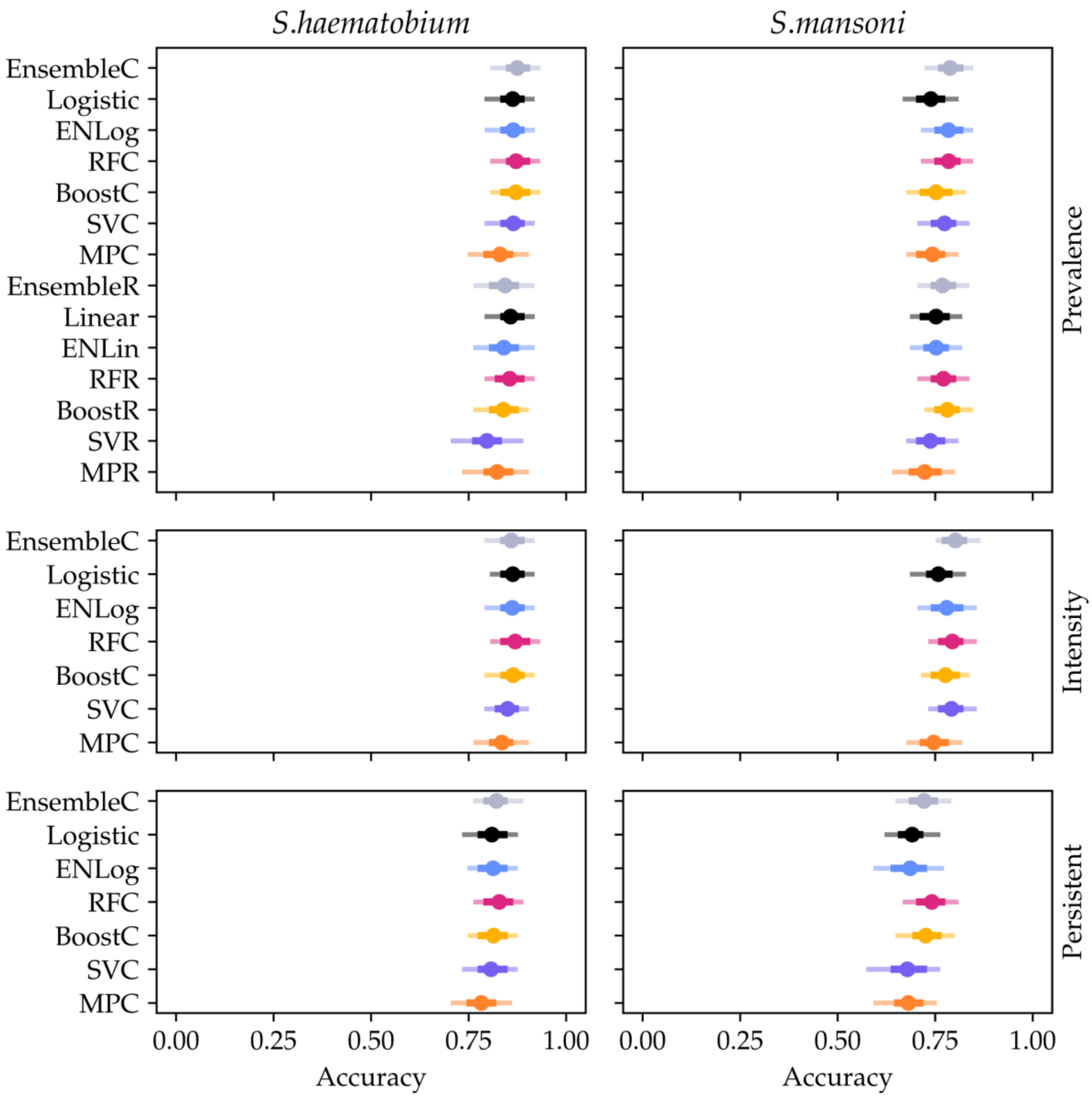

Figure S25: Accuracy of each model type in 500 resampled runs in the combined countries test set, by species and hotspot definition. Dots indicate median performance, with thick bars showing the inter-quartile range and thin bars showing the inter-decile range. The hotspot definitions are, from top to bottom: 1) prevalence of *Schistosoma* infection in year 5 >10%; 2) prevalence of moderate and heavy intensity infection in year 5 >1% and prevalence of infection in year 5 >10%; and 3) relative reduction in infection prevalence from year 1 to year 5 <35% and prevalence of infection in year 5 >10%. Model abbreviations:, *EnsembleC*—Ensemble classifier, *Logistic*—Logistic regression with forward variable selection, *ENLog*—Logistic regression with elastic net, *RFC*—Random forest classification, *BoostC*—Boosted decision trees, *SVC*—Support vector classifier, *MPC*—Multilayer perceptron classifier, *EnsembleR*—Ensemble regressor, *Linear*—Linear regression with forward variable selection, *ENLin*—Linear regression with elastic net, *RFR*—Random forest regression, *BoostR*—Boosted regression trees, *SVR*—Support vector regressor, *MPR*—Multilayer perceptron regressor.

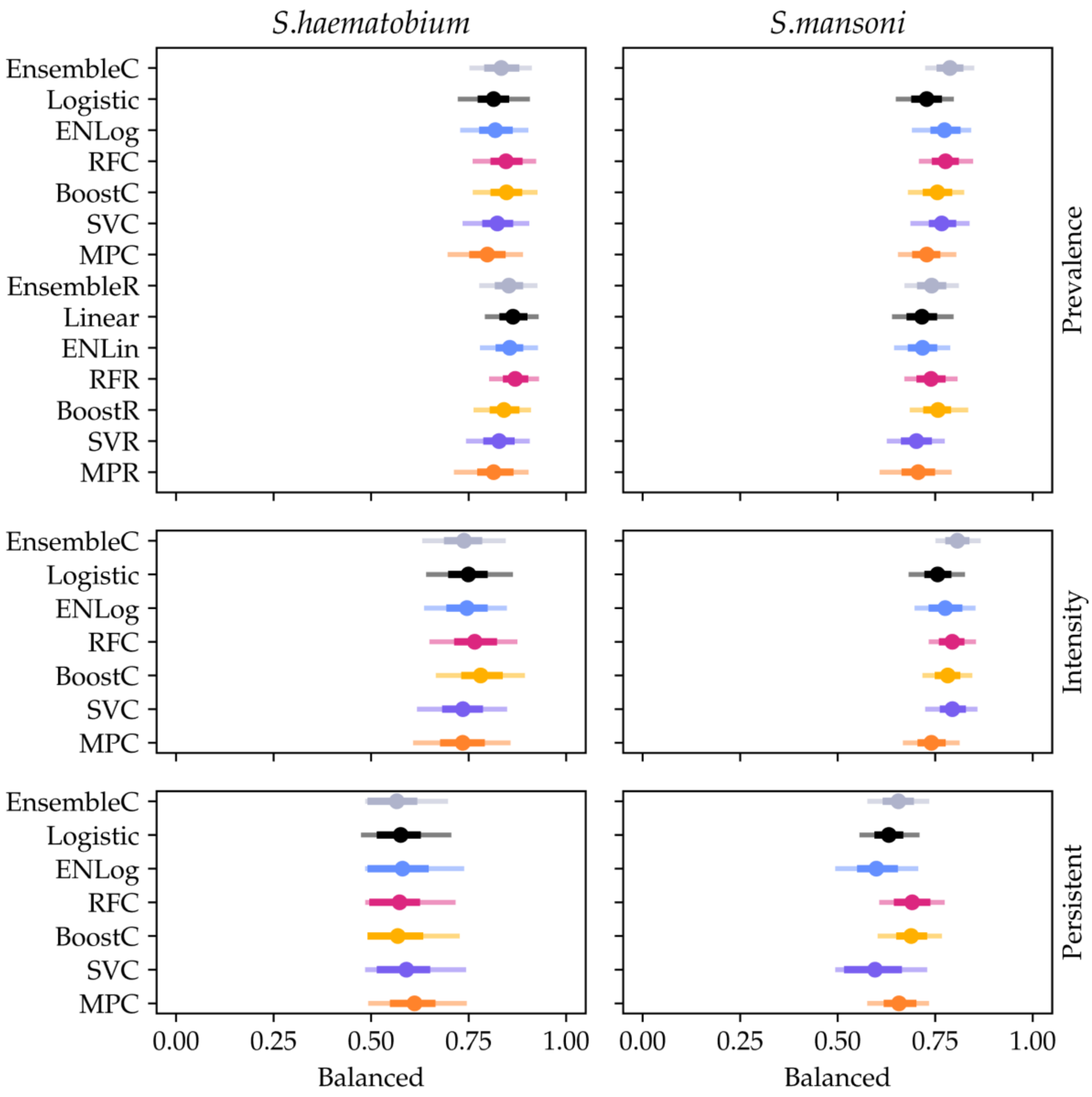

Figure S26: Balanced accuracy (mean of sensitivity and specificity) of each model type in 500 resampled runs in the combined countries test set, by species and hotspot definition. Dots indicate median performance, with thick bars showing the inter-quartile range and thin bars showing the inter-decile range. The hotspot definitions are, from top to bottom: 1) prevalence of Schistosoma infection in year 5 >10%; 2) prevalence of moderate and heavy intensity infection in year 5 >1% and prevalence of infection in year 5 >10%; and 3) relative reduction in infection prevalence from year 1 to year 5 <35% and prevalence of infection in year 5 >10%. Model abbreviations:, *EnsembleC*—Ensemble classifier, *Logistic*—Logistic regression with forward variable selection, *ENLog*—Logistic regression with elastic net, *RFC*—Random forest classification, *BoostC*—Boosted decision trees, *SVC*—Support vector classifier, *MPC*—Multilayer perceptron classifier, *EnsembleR*—Ensemble regressor, *Linear*—Linear regression with forward variable selection, *ENLin*—Linear regression with elastic net, *RFR*—Random forest regression, *BoostR*—Boosted regression trees, *SVR*—Support vector regressor, *MPR*—Multilayer perceptron regressor.

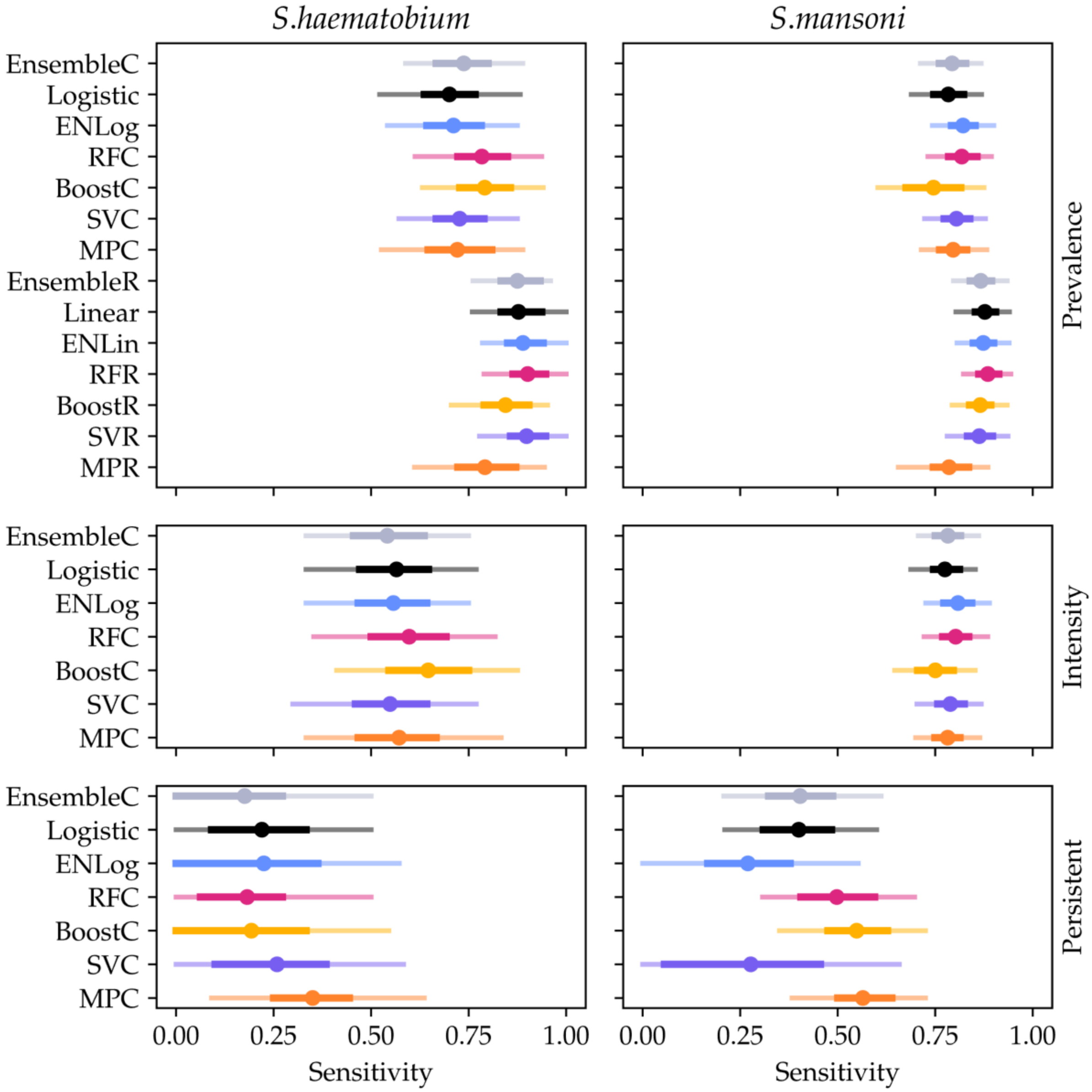

Figure S27: Sensitivity of each model type in 500 resampled runs in the combined countries test set, by species and hotspot definition. Dots indicate median performance, with thick bars showing the inter-quartile range and thin bars showing the inter-decile range. The hotspot definitions are, from top to bottom: 1) prevalence of Schistosoma infection in year 5 >10%; 2) prevalence of moderate and heavy intensity infection in year 5 >1% and prevalence of infection in year 5 >10%; and 3) relative reduction in infection prevalence from year 1 to year 5 <35% and prevalence of infection in year 5 >10%. Model abbreviations:, *EnsembleC*—Ensemble classifier, *Logistic*—Logistic regression with forward variable selection, *ENLog*—Logistic regression with elastic net, *RFC*—Random forest classification, *BoostC*—Boosted decision trees, *SVC*—Support vector classifier, *MPC*—Multilayer perceptron classifier, *EnsembleR*—Ensemble regressor, *Linear*—Linear regression with forward variable selection, *ENLin*—Linear regression with elastic net, *RFR*—Random forest regression, *BoostR*—Boosted regression trees, *SVR*—Support vector regressor, *MPR*—Multilayer perceptron regressor.

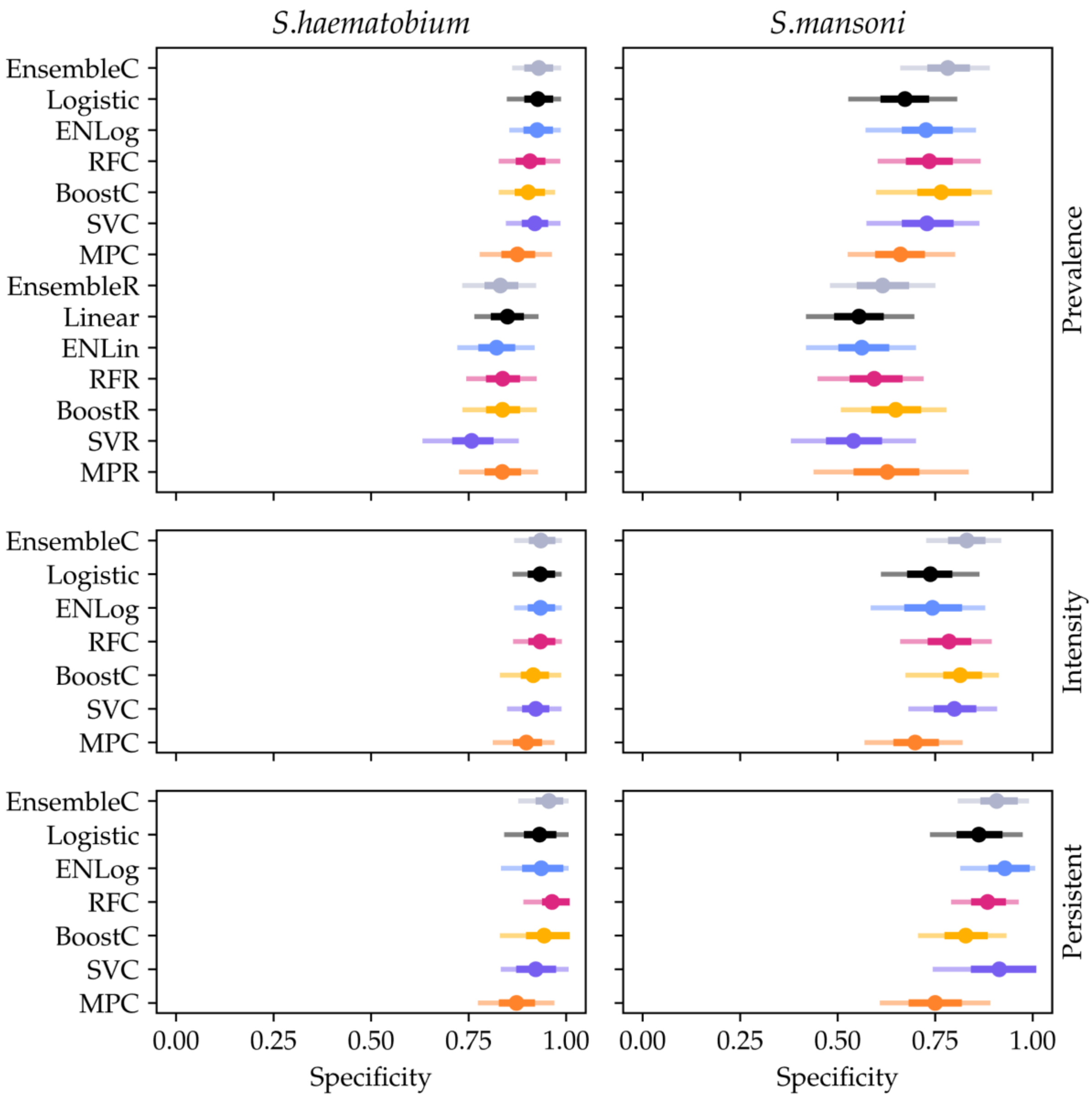

Figure S28: Specificity of each model type in 500 resampled runs in the combined countries test set, by species and hotspot definition. Dots indicate median performance, with thick bars showing the inter-quartile range and thin bars showing the inter-decile range. The hotspot definitions are, from top to bottom: 1) prevalence of Schistosoma infection in year 5 >10%; 2) prevalence of moderate and heavy intensity infection in year 5 >1% and prevalence of infection in year 5 >10%; and 3) relative reduction in infection prevalence from year 1 to year 5 <35% and prevalence of infection in year 5 >10%. Model abbreviations:, *EnsembleC*—Ensemble classifier, *Logistic*—Logistic regression with forward variable selection, *ENLog*—Logistic regression with elastic net, *RFC*—Random forest classification, *BoostC*—Boosted decision trees, *SVC*—Support vector classifier, *MPC*—Multilayer perceptron classifier, *EnsembleR*—Ensemble regressor, *Linear*—Linear regression with forward variable selection, *ENLin*—Linear regression with elastic net, *RFR*—Random forest regression, *BoostR*—Boosted regression trees, *SVR*—Support vector regressor, *MPR*—Multilayer perceptron regressor.

Table S3: Number of communities included in each training and test set.

Species	*S. haematobium*			*S. mansoni*					
	Combined	Between	Within	Combined	Between			Within	
Test data country		MOZ	NER		CIV	KEN	TZA	KEN	TZA
Train	161	210	147	252	320	162	236	138	87
Test	69	20	63	107	39	197	123	59	36
Total		230	210				359	197	123

Country abbreviations: MOZ—Mozambique, NER—Niger, CIV—Côte d'Ivoire, KEN—Kenya, TZA—Tanzania.

Table S4: Proportion of hotspots in each training and test set.

Species			*S. haematobium*			*S. mansoni*					
			Combined	Between	Within	Combined	Between			Within	
Test data country				MOZ	NER		CIV	KEN	TZA	KEN	TZA
Prevalence hotspot	(%)	Train	28.0	24.3	23.8	62.3	64.1	74.1	49.6	49.3	87.3
		Test	30.4	75.0	25.4	59.8	41.0	51.3	84.6	55.9	77.8
Intensity hotspot	(%)	Train	19.3	13.8	12.2	59.1	60.9	69.8	45.8	45.7	83.9
		Test	18.8	75.0	17.5	55.1	33.3	48.2	81.3	54.2	75.0
Persistent hotspot	(%)	Train	18.0	15.2	14.3	35.7	37.5	55.6	22.0	18.8	65.5
		Test	15.9	40.0	17.5	38.3	28.2	20.8	64.2	25.4	61.1

Country abbreviations: MOZ—Mozambique, NER—Niger, CIV—Côte d'Ivoire, KEN—Kenya, TZA—Tanzania.

Table S5: Accuracy (with 95% CI) of each model trained on the epidemiologic and secondary data sets, by hotspot definition and test set.

Species		*S. haematobium*			*S. mansoni*					
		Combined	Between	Within	Combined	Between			Within	
Test data country			MOZ*	NER		CIV*	KEN	TZA	KEN	TZA*
Prevalence hotspot	EnsembleClassifier	86 (75–92)	25 (11–47)	92 (83–97)	81 (73–88)	59 (43–73)	60 (53–66)	85 (78–91)	80 (68–88)	86 (71–94)
	LogisticRegression	86 (75–92)	85 (64–95)	83 (71–90)	75 (66–82)	59 (43–73)	54 (47–61)	75 (66–82)	80 (68–88)	81 (65–90)
	ElasticNetLogistic	86 (75–92)	65 (43–82)	90 (81–96)	81 (73–88)	59 (43–73)	53 (46–60)	84 (76–89)	75 (62–84)	86 (71–94)
	RandomForestClassifier	90 (81–95)	30 (15–52)	92 (83–97)	79 (71–86)	62 (46–75)	63 (57–70)	76 (68–83)	78 (66–87)	81 (65–90)
	GradientBoostingClassifier	90 (81–95)	60 (39–78)	87 (77–93)	71 (62–79)	59 (43–73)	62 (55–68)	74 (66–81)	86 (75–93)	81 (65–90)
	SupportVectorClassifier	87 (77–93)	25 (11–47)	87 (77–93)	81 (73–88)	59 (43–73)	56 (49–63)	84 (76–89)	78 (66–87)	83 (68–92)
	MultilayerPerceptronClassifier	87 (77–93)	25 (11–47)	92 (83–97)	70 (61–78)	59 (43–73)	65 (59–72)	84 (76–89)	76 (64–85)	83 (68–92)
	EnsembleRegressor	88 (79–94)	75 (53–89)	79 (68–88)	79 (70–85)	59 (43–73)	52 (45–59)	85 (77–90)	75 (62–84)	83 (68–92)
	LinearRegression	90 (81–95)	75 (53–89)	86 (75–92)	79 (71–86)	59 (43–73)	52 (45–59)	78 (70–84)	73 (60–83)	81 (65–90)
	ElasticNetLinear	88 (79–94)	75 (53–89)	84 (73–91)	77 (68–84)	59 (43–73)	51 (44–58)	81 (74–87)	71 (59–81)	86 (71–94)
	RandomForestRegressor	90 (81–95)	75 (53–89)	86 (75–92)	77 (68–84)	56 (41–71)	51 (44–58)	86 (79–91)	73 (60–83)	78 (62–88)
	GradientBoostingRegressor	86 (75–92)	75 (53–89)	81 (70–89)	77 (68–84)	59 (43–73)	55 (48–62)	87 (80–92)	80 (68–88)	81 (65–90)
	SupportVectorRegressor	83 (72–90)	25 (11–47)	75 (63–84)	80 (72–87)	59 (43–73)	51 (44–58)	83 (75–89)	71 (59–81)	83 (68–92)
	MultilayerPerceptronRegressor	88 (79–94)	70 (48–85)	79 (68–88)	70 (61–78)	54 (39–68)	69 (62–75)	82 (74–88)	68 (55–78)	69 (53–82)
Intensity hotspot	EnsembleClassifier	90 (81–95)	70 (48–85)	84 (73–91)	82 (74–88)	67 (51–79)	61 (54–67)	83 (75–89)	83 (72–91)	83 (68–92)
	LogisticRegression	88 (79–94)	65 (43–82)	86 (75–92)	77 (68–84)	67 (51–79)	53 (46–60)	76 (68–83)	73 (60–83)	72 (56–84)
	ElasticNetLogistic	88 (79–94)	55 (34–74)	83 (71–90)	82 (74–88)	67 (51–79)	57 (50–64)	80 (73–87)	83 (72–91)	81 (65–90)
	RandomForestClassifier	84 (74–91)	45 (26–66)	84 (73–91)	86 (78–91)	69 (54–81)	62 (55–69)	80 (72–86)	78 (66–87)	81 (65–90)
	GradientBoostingClassifier	91 (82–96)	80 (58–92)	84 (73–91)	77 (68–84)	67 (51–79)	62 (55–69)	84 (76–89)	78 (66–87)	81 (65–90)
	SupportVectorClassifier	86 (75–92)	25 (11–47)	86 (75–92)	83 (75–89)	67 (51–79)	60 (53–67)	83 (75–89)	83 (72–91)	78 (62–88)
	MultilayerPerceptronClassifier	87 (77–93)	60 (39–78)	81 (70–89)	64 (54–72)	67 (51–79)	54 (47–61)	82 (74–88)	73 (60–83)	78 (62–88)
Persistent hotspot	EnsembleClassifier	83 (72–90)	60 (39–78)	86 (75–92)	72 (63–80)	72 (56–83)	21 (16–28)	42 (34–51)	80 (68–88)	64 (48–78)
	LogisticRegression	81 (70–89)	55 (34–74)	78 (66–86)	64 (54–72)	72 (56–83)	21 (16–28)	37 (29–45)	80 (68–88)	58 (42–73)
	ElasticNetLogistic	83 (72–90)	55 (34–74)	83 (71–90)	69 (60–77)	72 (56–83)	21 (16–28)	45 (36–54)	78 (66–87)	64 (48–78)
	RandomForestClassifier	84 (74–91)	60 (39–78)	83 (71–90)	72 (63–80)	72 (56–83)	29 (24–36)	46 (38–55)	78 (66–87)	67 (50–80)
	GradientBoostingClassifier	87 (77–93)	60 (39–78)	83 (71–90)	74 (65–81)	67 (51–79)	24 (18–30)	46 (37–54)	80 (68–88)	64 (48–78)
	SupportVectorClassifier	83 (72–90)	60 (39–78)	87 (77–93)	67 (58–75)	72 (56–83)	21 (16–28)	36 (28–45)	81 (70–89)	42 (27–58)
	MultilayerPerceptronClassifier	83 (72–90)	50 (30–70)	83 (71–90)	64 (54–72)	72 (56–83)	31 (25–38)	39 (31–48)	78 (66–87)	56 (40–70)

See the Methods section (*Model training and validation*) for a full description of test sets: combined country, between country, and within country.
Country abbreviations: MOZ—Mozambique, NER—Niger, CIV—Côte d'Ivoire, KEN—Kenya, TZA—Tanzania.
*Note: Model performance was very poor for these models and test sets. These test sets each contained less than 50 communities, highlighting the challenge of model development and evaluation with a limited sample size. In particular, the 'between country' test sets performed poorly in general.

Table S6: Balanced accuracy (mean of sensitivity and specificity, with 95% CI) of each model trained on the epidemiologic and secondary data sets, by hotspot definition and test set.

Species		*S. haematobium*			*S. mansoni*					
		Combined	Between	Within	Combined	Between			Within	
Test data country			MOZ*	NER		CIV*	KEN	TZA	KEN	TZA*
Prevalence hotspot	EnsembleClassifier	80 (69–88)	50 (30–70)	88 (78–94)	81 (72–87)	50 (35–65)	59 (52–66)	72 (63–79)	80 (68–88)	69 (52–81)
	LogisticRegression	83 (72–90)	83 (62–94)	78 (66–86)	73 (64–81)	50 (35–65)	53 (46–60)	76 (68–83)	80 (68–88)	56 (40–71)
	ElasticNetLogistic	80 (69–88)	50 (30–70)	87 (77–94)	80 (72–87)	50 (35–65)	52 (45–59)	69 (60–76)	75 (63–84)	73 (57–85)
	RandomForestClassifier	89 (79–94)	47 (27–67)	91 (81–96)	78 (70–85)	53 (38–68)	63 (56–69)	77 (69–84)	78 (66–87)	56 (40–71)
	GradientBoostingClassifier	89 (79–94)	40 (22–61)	85 (75–92)	70 (60–78)	50 (35–65)	61 (54–68)	76 (68–83)	86 (75–93)	56 (40–71)
	SupportVectorClassifier	83 (72–90)	50 (30–70)	85 (75–92)	79 (71–86)	50 (35–65)	55 (48–62)	52 (43–60)	78 (66–87)	62 (46–76)
	MultilayerPerceptronClassifier	84 (74–91)	50 (30–70)	88 (78–94)	67 (58–75)	50 (35–65)	65 (58–71)	50 (41–58)	75 (63–84)	62 (46–76)
	EnsembleRegressor	89 (79–94)	50 (30–70)	80 (69–88)	76 (67–83)	50 (35–65)	51 (44–58)	52 (43–61)	73 (61–83)	67 (51–80)
	LinearRegression	90 (81–95)	50 (30–70)	84 (73–91)	77 (69–84)	50 (35–65)	51 (44–57)	66 (57–73)	71 (58–81)	61 (44–75)
	ElasticNetLinear	89 (79–94)	50 (30–70)	85 (74–92)	74 (65–82)	50 (35–65)	50 (43–57)	57 (48–65)	69 (56–79)	73 (57–85)
	RandomForestRegressor	93 (84–97)	50 (30–70)	86 (76–93)	74 (65–81)	61 (46–75)	50 (43–57)	70 (62–78)	72 (59–82)	50 (34–66)
	GradientBoostingRegressor	87 (77–93)	50 (30–70)	83 (72–90)	74 (65–81)	50 (35–65)	54 (47–61)	64 (56–72)	80 (68–88)	61 (44–75)
	SupportVectorRegressor	85 (75–91)	50 (30–70)	81 (70–89)	78 (70–85)	50 (35–65)	50 (43–57)	60 (51–68)	70 (57–80)	67 (51–80)
	MultilayerPerceptronRegressor	89 (79–94)	47 (27–67)	80 (69–88)	65 (56–74)	46 (31–61)	69 (62–75)	51 (42–59)	66 (54–77)	58 (42–73)
Intensity hotspot	EnsembleClassifier	79 (68–87)	73 (51–88)	65 (53–76)	82 (74–88)	50 (35–65)	62 (55–69)	83 (75–88)	84 (72–91)	67 (50–80)
	LogisticRegression	75 (64–84)	70 (48–85)	66 (54–77)	76 (67–83)	50 (35–65)	55 (48–62)	77 (69–84)	73 (61–83)	56 (40–70)
	ElasticNetLogistic	78 (67–86)	57 (36–76)	61 (48–72)	82 (74–88)	50 (35–65)	59 (52–65)	73 (64–80)	84 (72–91)	61 (45–75)
	RandomForestClassifier	72 (61–82)	57 (36–76)	65 (53–76)	85 (77–91)	54 (39–68)	63 (56–70)	82 (75–88)	78 (66–87)	61 (45–75)
	GradientBoostingClassifier	86 (76–92)	73 (51–88)	72 (60–82)	78 (69–85)	50 (35–65)	64 (57–70)	83 (76–89)	78 (66–86)	65 (48–78)
	SupportVectorClassifier	70 (59–80)	50 (30–70)	70 (58–80)	83 (75–89)	50 (35–65)	62 (55–68)	84 (77–90)	84 (72–91)	59 (43–74)
	MultilayerPerceptronClassifier	80 (69–88)	47 (27–67)	67 (55–77)	62 (53–71)	50 (35–65)	56 (49–62)	67 (59–75)	73 (60–82)	59 (43–74)
Persistent hotspot	EnsembleClassifier	57 (45–68)	50 (30–70)	63 (50–74)	65 (56–74)	50 (35–65)	50 (43–57)	54 (45–63)	69 (56–79)	57 (41–72)
	LogisticRegression	48 (37–60)	62 (41–80)	51 (39–63)	57 (48–66)	50 (35–65)	50 (43–57)	51 (42–59)	62 (49–73)	52 (36–67)
	ElasticNetLogistic	64 (52–74)	46 (26–67)	61 (48–72)	62 (52–70)	50 (35–65)	50 (43–57)	53 (45–62)	61 (48–72)	57 (41–72)
	RandomForestClassifier	50 (39–61)	50 (30–70)	54 (41–65)	65 (56–74)	50 (35–65)	50 (43–57)	57 (48–65)	68 (55–78)	61 (45–75)
	GradientBoostingClassifier	74 (62–83)	50 (30–70)	50 (38–62)	70 (61–78)	46 (32–62)	50 (43–57)	57 (48–65)	67 (54–77)	56 (40–71)
	SupportVectorClassifier	60 (48–71)	50 (30–70)	71 (59–81)	58 (49–67)	50 (35–65)	50 (43–57)	50 (41–59)	70 (57–80)	42 (27–58)
	MultilayerPerceptronClassifier	64 (52–74)	46 (26–67)	68 (56–78)	61 (52–70)	50 (35–65)	54 (47–61)	50 (41–58)	65 (53–76)	51 (35–66)

See the Methods section (*Model training and validation*) for a full description of test sets: combined country, between country, and within country.
Country abbreviations: MOZ—Mozambique, NER—Niger, CIV—Côte d'Ivoire, KEN—Kenya, TZA—Tanzania.
*Note: Model performance was very poor for these models and test sets. These test sets each contained less than 50 communities, highlighting the challenge of model development and evaluation with a limited sample size. In particular, the 'between country' test sets performed poorly in general.

Table S7: Sensitivity (with 95% CI) of each model trained on the epidemiologic and secondary data sets, by hotspot definition and test set.

Species		*S. haematobium*			*S. mansoni*					
		Combined	Between	Within	Combined	Between			Within	
Test data country			MOZ*	NER		CIV*	KEN	TZA	KEN	TZA*
Prevalence hotspot	EnsembleClassifier	67 (45–83)	0 (0–20)	81 (57–93)	83 (72–90)	0 (0–19)	98 (93–99)	91 (84–95)	76 (59–87)	100 (88–100)
	LogisticRegression	76 (55–89)	87 (62–96)	69 (44–86)	81 (70–89)	0 (0–19)	100 (96–100)	74 (65–82)	79 (62–89)	100 (88–100)
	ElasticNetLogistic	67 (45–83)	80 (55–93)	81 (57–93)	86 (75–92)	0 (0–19)	98 (93–99)	90 (83–95)	70 (53–83)	96 (82–99)
	RandomForestClassifier	86 (65–95)	13 (4–38)	88 (64–97)	84 (74–91)	6 (1–28)	98 (93–99)	76 (67–83)	76 (59–87)	100 (88–100)
	GradientBoostingClassifier	86 (65–95)	80 (55–93)	81 (57–93)	77 (65–85)	0 (0–19)	93 (86–97)	73 (64–81)	88 (73–95)	100 (88–100)
	SupportVectorClassifier	71 (50–86)	0 (0–20)	81 (57–93)	89 (79–95)	0 (0–19)	97 (92–99)	98 (93–99)	79 (62–89)	100 (88–100)
	MultilayerPerceptronClassifier	76 (55–89)	0 (0–20)	81 (57–93)	83 (72–90)	0 (0–19)	95 (89–98)	99 (95–100)	85 (69–93)	100 (88–100)
	EnsembleRegressor	90 (71–97)	100 (80–100)	81 (57–93)	88 (77–94)	0 (0–19)	99 (95–100)	99 (95–100)	85 (69–93)	96 (82–99)
	LinearRegression	90 (71–97)	100 (80–100)	81 (57–93)	88 (77–94)	0 (0–19)	100 (96–100)	84 (75–90)	88 (73–95)	96 (82–99)
	ElasticNetLinear	90 (71–97)	100 (80–100)	88 (64–97)	86 (75–92)	0 (0–19)	100 (96–100)	92 (86–96)	91 (76–97)	96 (82–99)
	RandomForestRegressor	100 (85–100)	100 (80–100)	88 (64–97)	89 (79–95)	88 (64–97)	100 (96–100)	93 (87–97)	82 (66–91)	100 (88–100)
	GradientBoostingRegressor	90 (71–97)	100 (80–100)	88 (64–97)	89 (79–95)	0 (0–19)	98 (93–99)	97 (92–99)	79 (62–89)	96 (82–99)
	SupportVectorRegressor	90 (71–97)	0 (0–20)	94 (72–99)	89 (79–95)	0 (0–19)	100 (96–100)	93 (87–97)	82 (66–91)	96 (82–99)
	MultilayerPerceptronRegressor	90 (71–97)	93 (70–99)	81 (57–93)	89 (79–95)	0 (0–19)	66 (57–75)	96 (91–98)	79 (62–89)	79 (60–90)
Intensity hotspot	EnsembleClassifier	62 (36–82)	67 (42–85)	36 (15–65)	83 (72–91)	0 (-0–23)	96 (90–98)	83 (74–89)	78 (61–89)	100 (88–100)
	LogisticRegression	54 (29–77)	60 (36–80)	36 (15–65)	85 (73–92)	0 (-0–23)	98 (93–99)	76 (67–83)	72 (55–84)	89 (72–96)
	ElasticNetLogistic	62 (36–82)	53 (30–75)	27 (10–57)	85 (73–92)	0 (-0–23)	97 (91–99)	85 (77–91)	78 (61–89)	100 (88–100)
	RandomForestClassifier	54 (29–77)	33 (15–58)	36 (15–65)	92 (82–96)	8 (1–33)	88 (80–93)	78 (69–85)	75 (58–87)	100 (88–100)
	GradientBoostingClassifier	77 (50–92)	87 (62–96)	55 (28–79)	64 (52–75)	0 (-0–23)	94 (87–97)	84 (76–90)	81 (65–91)	96 (82–99)
	SupportVectorClassifier	46 (23–71)	0 (0–20)	45 (21–72)	86 (75–93)	0 (-0–23)	98 (93–99)	82 (73–88)	78 (61–89)	96 (82–99)
	MultilayerPerceptronClassifier	69 (42–87)	73 (48–89)	45 (21–72)	73 (60–83)	0 (-0–23)	93 (86–96)	91 (84–95)	75 (58–87)	96 (82–99)
Persistent hotspot	EnsembleClassifier	18 (5–48)	0 (0–32)	27 (10–57)	37 (24–52)	0 (0–26)	100 (91–100)	13 (7–22)	47 (25–70)	86 (67–95)
	LogisticRegression	0 (0–26)	100 (68–100)	9 (2–38)	29 (18–44)	0 (0–26)	100 (91–100)	1 (0–7)	27 (11–52)	82 (61–93)
	ElasticNetLogistic	36 (15–65)	0 (0–32)	27 (10–57)	29 (18–44)	0 (0–26)	100 (91–100)	23 (15–33)	27 (11–52)	86 (67–95)
	RandomForestClassifier	0 (0–26)	0 (0–32)	9 (2–38)	37 (24–52)	0 (0–26)	85 (72–93)	20 (13–30)	47 (25–70)	86 (67–95)
	GradientBoostingClassifier	55 (28–79)	0 (0–32)	0 (0–26)	56 (41–70)	0 (0–26)	95 (84–99)	18 (11–28)	40 (20–64)	91 (72–97)
	SupportVectorClassifier	27 (10–57)	0 (0–32)	45 (21–72)	20 (10–34)	0 (0–26)	100 (91–100)	0 (0–5)	47 (25–70)	41 (23–61)
	MultilayerPerceptronClassifier	36 (15–65)	25 (7–59)	45 (21–72)	51 (36–66)	0 (0–26)	93 (81–97)	13 (7–22)	40 (20–64)	73 (52–87)

See the Methods section (*Model training and validation*) for a full description of test sets: combined country, between country, and within country.
Country abbreviations: MOZ—Mozambique, NER—Niger, CIV—Côte d'Ivoire, KEN—Kenya, TZA—Tanzania.
*Note: Model performance was very poor for these models and test sets. These test sets each contained less than 50 communities, highlighting the challenge of model development and evaluation with a limited sample size. In particular, the 'between country' test sets performed poorly in general.

Table S8: Specificity (with 95% CI) of each model trained on epidemiologic and secondary data sets, by hotspot definition and test set.

Species		*S. haematobium*			*S. mansoni*					
		Combined	Between	Within	Combined	Between			Within	
Test data country			MOZ*	NER		CIV*	KEN	TZA	KEN	TZA*
Prevalence hotspot	EnsembleClassifier	94 (83–98)	100 (57–100)	96 (86–99)	79 (65–89)	100 (86–100)	20 (13–29)	53 (32–73)	85 (66–94)	38 (14–69)
	LogisticRegression	90 (78–95)	80 (38–96)	87 (75–94)	65 (50–78)	100 (86–100)	6 (3–13)	79 (57–91)	81 (62–91)	12 (2–47)
	ElasticNetLogistic	94 (83–98)	20 (4–62)	94 (83–98)	74 (60–85)	100 (86–100)	6 (3–13)	47 (27–68)	81 (62–91)	50 (22–78)
	RandomForestClassifier	92 (80–97)	80 (38–96)	94 (83–98)	72 (57–83)	100 (86–100)	27 (19–37)	79 (57–91)	81 (62–91)	12 (2–47)
	GradientBoostingClassifier	92 (80–97)	0 (0–43)	89 (77–95)	63 (48–76)	100 (86–100)	29 (21–39)	79 (57–91)	85 (66–94)	12 (2–47)
	SupportVectorClassifier	94 (83–98)	100 (57–100)	89 (77–95)	70 (55–81)	100 (86–100)	14 (8–22)	5 (1–25)	77 (58–89)	25 (7–59)
	MultilayerPerceptronClassifier	92 (80–97)	100 (57–100)	96 (86–99)	51 (37–65)	100 (86–100)	34 (26–44)	0 (0–17)	65 (46–81)	25 (7–59)
	EnsembleRegressor	88 (75–94)	0 (0–43)	79 (65–88)	65 (50–78)	100 (86–100)	3 (1–9)	5 (1–25)	62 (43–78)	38 (14–69)
	LinearRegression	90 (78–95)	0 (0–43)	87 (75–94)	67 (53–80)	100 (86–100)	1 (0–6)	47 (27–68)	54 (35–71)	25 (7–59)
	ElasticNetLinear	88 (75–94)	0 (0–43)	83 (70–91)	63 (48–76)	100 (86–100)	0 (0–4)	21 (9–43)	46 (29–65)	50 (22–78)
	RandomForestRegressor	85 (73–93)	0 (0–43)	85 (72–93)	58 (43–72)	35 (19–55)	0 (0–4)	47 (27–68)	62 (43–78)	0 (0–32)
	GradientBoostingRegressor	83 (70–91)	0 (0–43)	79 (65–88)	58 (43–72)	100 (86–100)	9 (5–17)	32 (15–54)	81 (62–91)	25 (7–59)
	SupportVectorRegressor	79 (66–88)	100 (57–100)	68 (54–80)	67 (53–80)	100 (86–100)	0 (0–4)	26 (12–49)	58 (39–74)	38 (14–69)
	MultilayerPerceptronRegressor	88 (75–94)	0 (0–43)	79 (65–88)	42 (28–57)	91 (73–98)	71 (61–79)	5 (1–25)	54 (35–71)	38 (14–69)
Intensity hotspot	EnsembleClassifier	96 (88–99)	80 (38–96)	94 (84–98)	81 (68–90)	100 (87–100)	28 (21–38)	83 (63–93)	89 (72–96)	33 (12–65)
	LogisticRegression	96 (88–99)	80 (38–96)	96 (87–99)	67 (53–78)	100 (87–100)	12 (7–19)	78 (58–90)	74 (55–87)	22 (6–55)
	ElasticNetLogistic	95 (85–98)	60 (23–88)	94 (84–98)	79 (66–88)	100 (87–100)	21 (14–29)	61 (41–78)	89 (72–96)	22 (6–55)
	RandomForestClassifier	91 (81–96)	80 (38–96)	94 (84–98)	79 (66–88)	100 (87–100)	38 (29–48)	87 (68–95)	81 (63–92)	22 (6–55)
	GradientBoostingClassifier	95 (85–98)	60 (23–88)	90 (79–96)	92 (80–97)	100 (87–100)	33 (25–43)	83 (63–93)	74 (55–87)	33 (12–65)
	SupportVectorClassifier	95 (85–98)	100 (57–100)	94 (84–98)	79 (66–88)	100 (87–100)	25 (18–35)	87 (68–95)	89 (72–96)	22 (6–55)
	MultilayerPerceptronClassifier	91 (81–96)	20 (4–62)	88 (77–95)	52 (38–66)	100 (87–100)	19 (12–27)	43 (26–63)	70 (52–84)	22 (6–55)
Persistent hotspot	EnsembleClassifier	95 (86–98)	100 (76–100)	98 (90–100)	94 (85–98)	100 (88–100)	1 (0–4)	95 (85–99)	91 (79–96)	29 (12–55)
	LogisticRegression	97 (88–99)	25 (9–53)	92 (82–97)	85 (74–92)	100 (88–100)	1 (0–4)	100 (92–100)	98 (88–100)	21 (8–48)
	ElasticNetLogistic	91 (81–96)	92 (65–99)	94 (84–98)	94 (85–98)	100 (88–100)	1 (0–4)	84 (71–92)	95 (85–99)	29 (12–55)
	RandomForestClassifier	100 (94–100)	100 (76–100)	98 (90–100)	94 (85–98)	100 (88–100)	15 (10–21)	93 (82–98)	89 (76–95)	36 (16–61)
	GradientBoostingClassifier	93 (84–97)	100 (76–100)	100 (93–100)	85 (74–92)	93 (77–98)	5 (3–10)	95 (85–99)	93 (82–98)	21 (8–48)
	SupportVectorClassifier	93 (84–97)	100 (76–100)	96 (87–99)	97 (90–99)	100 (88–100)	1 (0–4)	100 (92–100)	93 (82–98)	43 (21–67)
	MultilayerPerceptronClassifier	91 (81–96)	67 (39–86)	90 (79–96)	71 (59–81)	100 (88–100)	15 (10–21)	86 (73–94)	91 (79–96)	29 (12–55)

See the Methods section (*Model training and validation*) for a full description of test sets: combined country, between country, and within country.
Country abbreviations: MOZ—Mozambique, NER—Niger, CIV—Côte d'Ivoire, KEN—Kenya, TZA—Tanzania.
*Note: Model performance was very poor for these models and test sets. These test sets each contained less than 50 communities, highlighting the challenge of model development and evaluation with a limited sample size. In particular, the 'between country' test sets performed poorly in general.

Table S9: False positive rate (complement of specificity) (with 95% CI) of each model trained on epidemiologic and secondary data sets, by hotspot definition and test set.

Species		*S. haematobium*			*S. mansoni*					
		Combined	Between	Within	Combined	Between			Within	
Test data country			MOZ*	NER		CIV*	KEN	TZA	KEN	TZA*
Prevalence hotspot	EnsembleClassifier	6 (2–17)	0 (0–43)	4 (1–14)	21 (11–35)	0 (0–14)	80 (71–87)	47 (27–68)	15 (6–34)	62 (31–86)
	LogisticRegression	10 (5–22)	20 (4–62)	13 (6–25)	35 (22–50)	0 (0–14)	94 (87–97)	21 (9–43)	19 (9–38)	88 (53–98)
	ElasticNetLogistic	6 (2–17)	80 (38–96)	6 (2–17)	26 (15–40)	0 (0–14)	94 (87–97)	53 (32–73)	19 (9–38)	50 (22–78)
	RandomForestClassifier	8 (3–20)	20 (4–62)	6 (2–17)	28 (17–43)	0 (0–14)	73 (63–81)	21 (9–43)	19 (9–38)	88 (53–98)
	GradientBoostingClassifier	8 (3–20)	100 (57–100)	11 (5–23)	37 (24–52)	0 (0–14)	71 (61–79)	21 (9–43)	15 (6–34)	88 (53–98)
	SupportVectorClassifier	6 (2–17)	0 (0–43)	11 (5–23)	30 (19–45)	0 (0–14)	86 (78–92)	95 (75–99)	23 (11–42)	75 (41–93)
	MultilayerPerceptronClassifier	8 (3–20)	0 (0–43)	4 (1–14)	49 (35–63)	0 (0–14)	66 (56–74)	100 (83–100)	35 (19–54)	75 (41–93)
	EnsembleRegressor	12 (6–25)	100 (57–100)	21 (12–35)	35 (22–50)	0 (0–14)	97 (91–99)	95 (75–99)	38 (22–57)	62 (31–86)
	LinearRegression	10 (5–22)	100 (57–100)	13 (6–25)	33 (20–47)	0 (0–14)	99 (94–100)	53 (32–73)	46 (29–65)	75 (41–93)
	ElasticNetLinear	12 (6–25)	100 (57–100)	17 (9–30)	37 (24–52)	0 (0–14)	100 (96–100)	79 (57–91)	54 (35–71)	50 (22–78)
	RandomForestRegressor	15 (7–27)	100 (57–100)	15 (7–28)	42 (28–57)	65 (45–81)	100 (96–100)	53 (32–73)	38 (22–57)	100 (68–100)
	GradientBoostingRegressor	17 (9–30)	100 (57–100)	21 (12–35)	42 (28–57)	0 (0–14)	91 (83–95)	68 (46–85)	19 (9–38)	75 (41–93)
	SupportVectorRegressor	21 (12–34)	0 (0–43)	32 (20–46)	33 (20–47)	0 (0–14)	100 (96–100)	74 (51–88)	42 (26–61)	62 (31–86)
	MultilayerPerceptronRegressor	12 (6–25)	100 (57–100)	21 (12–35)	58 (43–72)	9 (2–27)	29 (21–39)	95 (75–99)	46 (29–65)	62 (31–86)
Intensity hotspot	EnsembleClassifier	4 (1–12)	20 (4–62)	6 (2–16)	19 (10–32)	0 (0–13)	72 (62–79)	17 (7–37)	11 (4–28)	67 (35–88)
	LogisticRegression	4 (1–12)	20 (4–62)	4 (1–13)	33 (22–47)	0 (0–13)	88 (81–93)	22 (10–42)	26 (13–45)	78 (45–94)
	ElasticNetLogistic	5 (2–15)	40 (12–77)	6 (2–16)	21 (12–34)	0 (0–13)	79 (71–86)	39 (22–59)	11 (4–28)	78 (45–94)
	RandomForestClassifier	9 (4–19)	20 (4–62)	6 (2–16)	21 (12–34)	0 (0–13)	62 (52–71)	13 (5–32)	19 (8–37)	78 (45–94)
	GradientBoostingClassifier	5 (2–15)	40 (12–77)	10 (4–21)	8 (3–20)	0 (0–13)	67 (57–75)	17 (7–37)	26 (13–45)	67 (35–88)
	SupportVectorClassifier	5 (2–15)	0 (0–43)	6 (2–16)	21 (12–34)	0 (0–13)	75 (65–82)	13 (5–32)	11 (4–28)	78 (45–94)
	MultilayerPerceptronClassifier	9 (4–19)	80 (38–96)	12 (5–23)	48 (34–62)	0 (0–13)	81 (73–88)	57 (37–74)	30 (16–48)	78 (45–94)
Persistent hotspot	EnsembleClassifier	5 (2–14)	0 (0–24)	2 (0–10)	6 (2–15)	0 (0–12)	99 (96–100)	5 (1–15)	9 (4–21)	71 (45–88)
	LogisticRegression	3 (1–12)	75 (47–91)	8 (3–18)	15 (8–26)	0 (0–12)	99 (96–100)	0 (0–8)	2 (0–12)	79 (52–92)
	ElasticNetLogistic	9 (4–19)	8 (1–35)	6 (2–16)	6 (2–15)	0 (0–12)	99 (96–100)	16 (8–29)	5 (1–15)	71 (45–88)
	RandomForestClassifier	0 (0–6)	0 (0–24)	2 (0–10)	6 (2–15)	0 (0–12)	85 (79–90)	7 (2–18)	11 (5–24)	64 (39–84)
	GradientBoostingClassifier	7 (3–16)	0 (0–24)	0 (0–7)	15 (8–26)	7 (2–23)	95 (90–97)	5 (1–15)	7 (2–18)	79 (52–92)
	SupportVectorClassifier	7 (3–16)	0 (0–24)	4 (1–13)	3 (1–10)	0 (0–12)	99 (96–100)	0 (0–8)	7 (2–18)	57 (33–79)
	MultilayerPerceptronClassifier	9 (4–19)	33 (14–61)	10 (4–21)	29 (19–41)	0 (0–12)	85 (79–90)	14 (6–27)	9 (4–21)	71 (45–88)

See the Methods section (*Model training and validation*) for a full description of test sets: combined country, between country, and within country.
Country abbreviations: MOZ—Mozambique, NER—Niger, CIV—Côte d'Ivoire, KEN—Kenya, TZA—Tanzania.
*Note: Model performance was very poor for these models and test sets. These test sets each contained less than 50 communities, highlighting the challenge of model development and evaluation with a limited sample size. In particular, the 'between country' test sets performed poorly in general.

Table S10: F1 (harmonic mean of sensitivity and positive predictive value, with 95% CI) of each model trained on the epidemiologic and secondary data sets, by hotspot definition and test set.

Species		*S. haematobium*			*S. mansoni*					
		Combined	Between	Within	Combined	Between			Within	
Test data country			MOZ*	NER		CIV*	KEN	TZA	KEN	TZA*
Prevalence hotspot	EnsembleClassifier	74	—	84	84	—	71	91	81	92
	LogisticRegression	76	90	67	79	—	69	83	81	89
	ElasticNetLogistic	74	77	81	85	—	68	90	75	92
	RandomForestClassifier	84	22	85	83	12	73	84	79	89
	GradientBoostingClassifier	84	75	76	76	—	71	83	88	89
	SupportVectorClassifier	77	—	76	85	—	70	91	80	90
	MultilayerPerceptronClassifier	78	—	84	77	—	74	91	80	90
	EnsembleRegressor	83	86	67	83	—	68	92	79	90
	LinearRegression	84	86	74	84	—	68	87	78	89
	ElasticNetLinear	83	86	74	81	—	68	89	78	92
	RandomForestRegressor	86	86	76	82	62	68	92	77	88
	GradientBoostingRegressor	79	86	70	82	—	69	93	81	89
	SupportVectorRegressor	76	—	65	84	—	68	90	76	90
	MultilayerPerceptronRegressor	83	82	67	78	—	68	90	73	80
Intensity hotspot	EnsembleClassifier	70	77	44	84	—	70	89	83	90
	LogisticRegression	64	72	47	80	—	67	84	74	83
	ElasticNetLogistic	67	64	35	84	—	69	88	83	89
	RandomForestClassifier	56	48	44	88	14	69	86	79	89
	GradientBoostingClassifier	77	87	55	75	—	71	89	80	88
	SupportVectorClassifier	55	—	53	85	—	70	89	83	87
	MultilayerPerceptronClassifier	67	73	45	69	—	66	89	75	87
Persistent hotspot	EnsembleClassifier	25	—	40	50	0	35	22	54	75
	LogisticRegression	—	64	12	38	—	35	2	40	71
	ElasticNetLogistic	40	—	35	42	—	35	35	38	75
	RandomForestClassifier	—	—	15	50	—	33	33	52	76
	GradientBoostingClassifier	57	—	—	62	—	34	29	50	75
	SupportVectorClassifier	33	—	56	31	—	35	—	56	46
	MultilayerPerceptronClassifier	40	29	48	52	—	36	21	48	67

See the Methods section (*Model training and validation*) for a full description of test sets: combined country, between country, and within country.
Country abbreviations: MOZ—Mozambique, NER—Niger, CIV—Côte d'Ivoire, KEN—Kenya, TZA—Tanzania.
*Note: Model performance was very poor for these models and test sets. These test sets each contained less than 50 communities, highlighting the challenge of model development and evaluation with a limited sample size. In particular, the 'between country' test sets performed poorly in general.

Table S11: Area under receiver operating characteristic (ROC) curve of each classification model trained on the epidemiologic and secondary data sets, by hotspot definition and test set.

Species		*S. haematobium*			*S. mansoni*					
		Combined	Between	Within	Combined	Between			Within	
Test data country			MOZ*	NER		CIV*	KEN	TZA	KEN	TZA*
Prevalence hotspot	LogisticRegression	93	87	89	82	61	76	83	85	86
	ElasticNetLogistic	96	69	95	86	65	84	85	83	77
	RandomForestClassifier	97	40	95	85	63	83	87	85	69
	GradientBoostingClassifier	94	22	88	74	52	78	85	87	88
	SupportVectorClassifier	96	53	95	85	66	81	75	86	75
	MultilayerPerceptronClassifier	91	71	92	78	65	76	58	85	84
Intensity hotspot	LogisticRegression	97	84	82	87	69	75	86	86	81
	ElasticNetLogistic	95	61	87	88	70	84	87	85	86
	RandomForestClassifier	95	69	84	90	58	84	88	85	73
	GradientBoostingClassifier	96	75	82	78	68	84	88	78	65
	SupportVectorClassifier	90	67	79	88	70	84	88	86	86
	MultilayerPerceptronClassifier	87	47	87	75	67	62	80	80	79
Persistent hotspot	LogisticRegression	59	54	81	59	56	47	62	60	60
	ElasticNetLogistic	85	54	80	74	54	50	51	65	57
	RandomForestClassifier	86	55	83	78	54	59	66	72	60
	GradientBoostingClassifier	89	56	73	79	52	36	55	75	56
	SupportVectorClassifier	85	60	83	74	53	62	50	76	41
	MultilayerPerceptronClassifier	84	36	82	68	61	50	49	78	53

See the Methods section (*Model training and validation*) for a full description of test sets: combined country, between country, and within country.
Country abbreviations: MOZ—Mozambique, NER—Niger, CIV—Côte d'Ivoire, KEN—Kenya, TZA—Tanzania.
*Note: Model performance was very poor for these models and test sets. These test sets each contained less than 50 communities, highlighting the challenge of model development and evaluation with a limited sample size. In particular, the 'between country' test sets performed poorly in general.

Table S12: Coefficients of the highlighted linear regression model to predict year 5 prevalence in the *S. haematobium* combined countries test set.

Variable	Coefficient
Intercept	0.1101
Prevalence	0.1167

Hotspot predictions are derived from this model by dichotomizing at 10% prevalence.

Table S13: Coefficients of the highlighted logistic regression model with elastic net regularization to predict hotspots *S. mansoni* combined countries test set under the prevalence hotspot definition.

Variable	Coefficient
Intercept	0.9313
SAC Rounds	-0.3267
Prevalence	0.3853
Log Arithmetic Interaction	0.0903
Prevalence 5–8	0.1006
Prevalence 9–12	0.8320
Improved Water	-0.1341
DPT 3rd Dose	0.7245
Education Female	-0.1893
Education Male	-0.5699
Mortality Under-5	-0.3091
Vegetation	0.2114
Log Water Distance	-0.0951
Minimum Temperature	0.1878

This study is not a causal inference study, and these coefficients should not be taken to indicate causal relations between the listed variables and hotspot status.

Table S14: Odds ratios (with standard error) of scaled variables in bivariate logistic regressions for the combined countries training sets, by hotspot definition.

Species	*S. haematobium*			*S. mansoni*		
Hotspot definition	Prevalence	Intensity	Persistent	Prevalence	Intensity	Persistent
SAC Rounds	**0.55 (0.10)**	**0.41 (0.096)**	**0.45 (0.10)**	0.89 (0.12)	0.89 (0.11)	1.0 (0.13)
Community Rounds	1.1 (0.19)	1.0 (0.20)	0.95 (0.20)	1.3 (0.18)	1.3 (0.17)	1.2 (0.15)
Prevalence	**5.1 (1.4)**	**4.3 (1.0)**	**2.0 (0.38)**	**5.0 (1.1)**	**5.1 (1.1)**	**1.8 (0.25)**
Log Gemometric Interaction	**4.2 (1.1)**	**4.1 (1.0)**	**1.9 (0.34)**	**4.7 (1.0)**	**5.0 (1.1)**	**1.8 (0.26)**
Log Arithmetic Interaction	**4.2 (1.0)**	**4.2 (1.0)**	**2.0 (0.38)**	**4.2 (0.84)**	**4.3 (0.85)**	**1.9 (0.27)**
Log Dispersion	**2.6 (0.55)**	**2.4 (0.52)**	**1.9 (0.40)**	1.3 (0.17)	1.1 (0.14)	1.2 (0.16)
Log Mean Intensity	**3.7 (0.90)**	**4.2 (1.2)**	**2.3 (0.52)**	**2.5 (0.40)**	**2.5 (0.40)**	**1.8 (0.27)**
Prevalence 5-8	**5.5 (1.5)**	**4.3 (1.1)**	**2.2 (0.42)**	**7.7 (2.5)**	**8.8 (2.9)**	**2.2 (0.32)**
Log Mean Intensity 5-8	**3.9 (0.95)**	**4.0 (1.1)**	**2.4 (0.55)**	**2.7 (0.45)**	**3.2 (0.59)**	**2.3 (0.44)**
Prevalence 9-12	**4.9 (1.3)**	**4.0 (0.94)**	**2.0 (0.36)**	**4.7 (0.98)**	**4.6 (0.92)**	**1.9 (0.26)**
Log Mean Intensity 9-12	**3.6 (0.83)**	**4.4 (1.2)**	**2.2 (0.48)**	**2.2 (0.34)**	**2.2 (0.33)**	**1.6 (0.24)**
Improved Water	1.5 (0.28)	1.0 (0.21)	1.3 (0.28)	**0.75 (0.10)**	**0.73 (0.096)**	0.81 (0.11)
Improved Sanitation	**2.2 (0.46)**	**2.0 (0.41)**	**1.7 (0.32)**	**0.77 (0.10)**	**0.77 (0.10)**	**0.64 (0.093)**
3rd Dose DPT	1.2 (0.20)	**1.5 (0.26)**	1.1 (0.22)	**2.4 (0.46)**	**2.3 (0.43)**	**2.9 (0.75)**
Education Female	**2.0 (0.36)**	**2.0 (0.36)**	1.4 (0.24)	0.92 (0.12)	0.91 (0.12)	**0.73 (0.096)**
Education Male	**2.1 (0.39)**	**2.1 (0.39)**	**1.5 (0.27)**	0.85 (0.11)	0.84 (0.11)	**0.67 (0.089)**
Stunting Under-5	0.72 (0.14)	1.1 (0.21)	0.74 (0.16)	**1.7 (0.23)**	**1.7 (0.23)**	**2.4 (0.39)**
Underweight Under-5	**0.29 (0.071)**	**0.28 (0.074)**	**0.44 (0.098)**	0.96 (0.13)	0.97 (0.13)	0.87 (0.11)
Mortality Under-5	**0.48 (0.092)**	**0.43 (0.083)**	**0.60 (0.11)**	**0.64 (0.086)**	**0.63 (0.085)**	0.91 (0.12)
Relative Wealth	**1.5 (0.27)**	**1.5 (0.30)**	1.3 (0.25)	1.0 (0.13)	1.0 (0.13)	1.1 (0.14)
NDVI	**2.1 (0.43)**	**2.7 (0.60)**	**1.6 (0.29)**	0.78 (0.11)	0.79 (0.10)	**0.76 (0.10)**
Log Water Distance	0.78 (0.14)	0.94 (0.19)	0.66 (0.14)	**0.40 (0.065)**	**0.39 (0.063)**	**0.64 (0.091)**
Precipitation	**2.0 (0.37)**	**2.3 (0.43)**	**1.6 (0.26)**	**0.49 (0.071)**	**0.48 (0.070)**	**0.49 (0.084)**
Minimum Temperature	**2.8 (0.69)**	**2.5 (0.55)**	**2.1 (0.44)**	1.2 (0.16)	1.2 (0.16)	1.2 (0.16)
Log Population Density	1.1 (0.18)	0.90 (0.21)	0.58 (0.20)	1.3 (0.18)	1.2 (0.16)	1.0 (0.14)
Log Prevalence Density	**6.8 (2.2)**	**3.9 (1.1)**	**1.9 (0.41)**	**2.8 (0.47)**	**2.7 (0.45)**	**1.4 (0.20)**

Bold indicates p-value < 0.05. The magnitude of the odds ratio indicates how the odds of being a hotspot changes when comparing an average community to a community one standard deviation above the mean, for a given variable. This study is not a causal inference study, and these coefficients should not be taken to indicate causal relations between the listed variables and hotspot status. Some relationships may be driven by differences at the country level.

Table S15: Accuracy (with 95% CI) of each model trained on epidemiologic data alone, by hotspot definition and test set.

Species		*S. haematobium*			*S. mansoni*					
		Combined	Between	Within	Combined	Between			Within	
Test data country			MOZ*	NER		CIV*	KEN	TZA	KEN	TZA*
Prevalence hotspot	EnsembleClassifier	91 (82–96)	50 (30–70)	90 (81–96)	75 (66–82)	69 (54–81)	70 (63–76)	75 (66–82)	83 (72–91)	78 (62–88)
	LogisticRegression	88 (79–94)	85 (64–95)	86 (75–92)	75 (66–82)	41 (27–57)	66 (59–72)	75 (66–82)	80 (68–88)	78 (62–88)
	ElasticNetLogistic	91 (82–96)	55 (34–74)	92 (83–97)	68 (59–76)	69 (54–81)	67 (60–73)	76 (67–82)	80 (68–88)	78 (62–88)
	RandomForestClassifier	93 (84–97)	65 (43–82)	84 (73–91)	76 (67–83)	64 (48–77)	68 (61–74)	76 (67–82)	78 (66–87)	78 (62–88)
	GradientBoostingClassifier	91 (82–96)	90 (70–97)	86 (75–92)	75 (66–82)	72 (56–83)	70 (63–76)	77 (69–84)	75 (62–84)	81 (65–90)
	SupportVectorClassifier	91 (82–96)	50 (30–70)	89 (79–95)	72 (63–80)	56 (41–71)	68 (61–74)	72 (64–79)	81 (70–89)	78 (62–88)
	MultilayerPerceptronClassifier	88 (79–94)	45 (26–66)	84 (73–91)	79 (70–85)	46 (32–61)	70 (63–76)	72 (63–79)	69 (57–80)	81 (65–90)
	EnsembleRegressor	88 (79–94)	75 (53–89)	84 (73–91)	72 (63–80)	62 (46–75)	59 (52–66)	82 (74–88)	75 (62–84)	78 (62–88)
	LinearRegression	90 (81–95)	80 (58–92)	86 (75–92)	70 (61–78)	41 (27–57)	61 (54–68)	81 (74–87)	76 (64–85)	78 (62–88)
	ElasticNetLinear	86 (75–92)	90 (70–97)	84 (73–91)	68 (59–76)	69 (54–81)	63 (57–70)	81 (74–87)	66 (53–77)	78 (62–88)
	RandomForestRegressor	88 (79–94)	80 (58–92)	84 (73–91)	72 (63–80)	56 (41–71)	59 (52–66)	80 (72–86)	69 (57–80)	78 (62–88)
	GradientBoostingRegressor	91 (82–96)	80 (58–92)	83 (71–90)	71 (62–79)	54 (39–68)	63 (56–69)	80 (72–86)	76 (64–85)	78 (62–88)
	SupportVectorRegressor	86 (75–92)	75 (53–89)	75 (63–84)	67 (58–75)	49 (34–64)	59 (52–66)	83 (75–89)	69 (57–80)	81 (65–90)
	MultilayerPerceptronRegressor	90 (81–95)	75 (53–89)	84 (73–91)	77 (68–84)	49 (34–64)	63 (56–69)	78 (70–84)	71 (59–81)	75 (59–86)
Intensity hotspot	EnsembleClassifier	87 (77–93)	75 (53–89)	83 (71–90)	80 (72–87)	77 (62–87)	71 (64–77)	72 (64–79)	85 (73–92)	81 (65–90)
	LogisticRegression	90 (81–95)	80 (58–92)	81 (70–89)	79 (70–85)	49 (34–64)	69 (62–75)	76 (68–83)	76 (64–85)	81 (65–90)
	ElasticNetLogistic	90 (81–95)	80 (58–92)	83 (71–90)	79 (71–86)	79 (64–89)	70 (63–76)	75 (66–82)	85 (73–92)	75 (59–86)
	RandomForestClassifier	93 (84–97)	80 (58–92)	84 (73–91)	81 (73–88)	64 (48–77)	70 (63–76)	78 (70–84)	81 (70–89)	83 (68–92)
	GradientBoostingClassifier	86 (75–92)	80 (58–92)	84 (73–91)	78 (69–84)	72 (56–83)	70 (63–76)	76 (67–82)	78 (66–87)	78 (62–88)
	SupportVectorClassifier	88 (79–94)	85 (64–95)	86 (75–92)	78 (69–84)	67 (51–79)	70 (63–76)	72 (64–79)	83 (72–91)	75 (59–86)
	MultilayerPerceptronClassifier	83 (72–90)	40 (22–61)	83 (71–90)	72 (63–80)	59 (43–73)	72 (65–77)	68 (60–76)	71 (59–81)	78 (62–88)
Persistent hotspot	EnsembleClassifier	84 (74–91)	60 (39–78)	83 (71–90)	69 (60–77)	69 (54–81)	61 (54–67)	44 (35–53)	78 (66–87)	56 (40–70)
	LogisticRegression	84 (74–91)	60 (39–78)	83 (71–90)	64 (55–73)	72 (56–83)	62 (55–69)	36 (28–45)	80 (68–88)	69 (53–82)
	ElasticNetLogistic	84 (74–91)	35 (18–57)	83 (71–90)	67 (58–75)	77 (62–87)	56 (49–63)	41 (32–49)	80 (68–88)	64 (48–78)
	RandomForestClassifier	86 (75–92)	70 (48–85)	83 (71–90)	65 (56–74)	69 (54–81)	56 (49–63)	46 (38–55)	78 (66–87)	61 (45–75)
	GradientBoostingClassifier	86 (75–92)	75 (53–89)	83 (71–90)	69 (60–77)	64 (48–77)	56 (49–63)	46 (37–54)	75 (62–84)	53 (37–68)
	SupportVectorClassifier	83 (72–90)	60 (39–78)	83 (71–90)	68 (59–76)	62 (46–75)	56 (49–63)	44 (35–53)	80 (68–88)	61 (45–75)
	MultilayerPerceptronClassifier	80 (69–88)	60 (39–78)	87 (77–93)	67 (58–75)	38 (25–54)	48 (41–55)	54 (45–62)	75 (62–84)	47 (32–63)

See the Methods section (*Model training and validation*) for a full description of test sets: combined country, between country, and within country.
Country abbreviations: MOZ—Mozambique, NER—Niger, CIV—Côte d'Ivoire, KEN—Kenya, TZA—Tanzania.
*Note: Model performance was very poor for these models and test sets. These test sets each contained less than 50 communities, highlighting the challenge of model development and evaluation with a limited sample size. In particular, the 'between country' test sets performed poorly in general.

Table S16: Balanced accuracy (with 95% CI) of each model trained on epidemiologic data alone, by hotspot definition and test set.

Species		*S. haematobium*			*S. mansoni*					
		Combined	Between	Within	Combined	Between			Within	
Test data country			MOZ*	NER		CIV*	KEN	TZA	KEN	TZA*
Prevalence hotspot	EnsembleClassifier	88 (79–94)	60 (39–78)	87 (77–94)	73 (64–81)	64 (49–78)	69 (62–75)	76 (68–83)	84 (73–91)	50 (34–66)
	LogisticRegression	84 (73–91)	77 (55–90)	76 (64–85)	72 (63–80)	46 (32–61)	66 (59–72)	76 (68–83)	80 (68–88)	50 (34–66)
	ElasticNetLogistic	87 (77–93)	70 (48–85)	86 (76–93)	65 (56–73)	64 (49–78)	66 (60–73)	75 (66–82)	80 (68–88)	50 (34–66)
	RandomForestClassifier	92 (83–96)	57 (36–76)	83 (72–90)	76 (67–83)	60 (44–74)	68 (61–74)	77 (69–84)	78 (66–87)	50 (34–66)
	GradientBoostingClassifier	90 (80–95)	80 (58–92)	86 (76–93)	74 (65–81)	68 (53–81)	70 (63–76)	74 (65–81)	76 (63–85)	56 (40–71)
	SupportVectorClassifier	88 (79–94)	60 (39–78)	84 (73–91)	69 (60–77)	54 (39–69)	68 (61–74)	75 (67–82)	83 (71–90)	50 (34–66)
	MultilayerPerceptronClassifier	86 (76–93)	50 (30–70)	85 (74–92)	79 (70–85)	51 (36–65)	70 (63–76)	72 (64–80)	68 (55–78)	65 (49–79)
	EnsembleRegressor	89 (79–94)	50 (30–70)	85 (74–92)	68 (58–76)	59 (43–73)	58 (52–65)	72 (64–79)	73 (61–83)	50 (34–66)
	LinearRegression	90 (81–95)	60 (39–78)	84 (73–91)	66 (57–74)	48 (33–63)	61 (54–67)	72 (63–79)	75 (62–84)	50 (34–66)
	ElasticNetLinear	87 (77–93)	80 (58–92)	83 (72–90)	64 (54–72)	66 (51–79)	63 (56–69)	70 (61–77)	64 (51–75)	50 (34–66)
	RandomForestRegressor	90 (81–95)	60 (39–78)	85 (74–92)	67 (58–75)	59 (44–73)	59 (52–65)	71 (62–78)	69 (56–79)	50 (34–66)
	GradientBoostingRegressor	92 (84–97)	60 (39–78)	84 (73–91)	68 (59–76)	53 (38–68)	62 (55–69)	75 (67–82)	76 (64–85)	50 (34–66)
	SupportVectorRegressor	87 (77–93)	50 (30–70)	77 (65–85)	63 (53–71)	51 (36–66)	58 (51–65)	66 (58–74)	67 (55–78)	56 (40–71)
	MultilayerPerceptronRegressor	89 (79–94)	50 (30–70)	83 (72–90)	73 (64–81)	50 (35–65)	62 (55–69)	70 (61–77)	71 (58–81)	53 (37–68)
Intensity hotspot	EnsembleClassifier	71 (60–81)	77 (55–90)	61 (48–72)	80 (72–87)	67 (52–80)	72 (65–77)	78 (70–84)	85 (74–92)	61 (45–75)
	LogisticRegression	76 (65–85)	73 (51–88)	56 (44–68)	78 (70–85)	52 (37–67)	70 (63–76)	77 (69–84)	76 (64–85)	61 (45–75)
	ElasticNetLogistic	76 (65–85)	73 (51–88)	61 (48–72)	79 (70–86)	69 (54–81)	70 (63–76)	79 (72–86)	85 (74–92)	50 (34–66)
	RandomForestClassifier	84 (73–91)	73 (51–88)	65 (53–76)	82 (73–88)	54 (39–68)	71 (64–77)	81 (74–87)	82 (70–89)	67 (50–80)
	GradientBoostingClassifier	67 (56–77)	73 (51–88)	72 (60–82)	78 (69–85)	63 (48–77)	71 (64–76)	77 (68–83)	78 (66–86)	56 (40–70)
	SupportVectorClassifier	78 (67–86)	83 (62–94)	70 (58–80)	76 (67–83)	63 (48–77)	70 (64–76)	78 (70–84)	84 (72–91)	50 (34–66)
	MultilayerPerceptronClassifier	69 (57–78)	40 (22–61)	68 (56–78)	71 (62–79)	52 (37–67)	72 (65–77)	70 (62–78)	71 (59–81)	67 (50–80)
Persistent hotspot	EnsembleClassifier	50 (39–61)	50 (30–70)	50 (38–62)	61 (52–70)	51 (36–66)	56 (49–63)	54 (46–63)	61 (48–72)	47 (32–63)
	LogisticRegression	54 (42–65)	50 (30–70)	50 (38–62)	58 (48–67)	50 (35–65)	58 (51–65)	50 (41–59)	62 (49–73)	62 (46–76)
	ElasticNetLogistic	50 (39–61)	35 (18–57)	50 (38–62)	60 (50–68)	59 (44–73)	59 (52–66)	52 (43–60)	60 (47–72)	54 (38–69)
	RandomForestClassifier	55 (43–66)	67 (45–83)	50 (38–62)	58 (49–67)	54 (38–68)	55 (48–62)	52 (43–61)	61 (48–72)	54 (38–69)
	GradientBoostingClassifier	58 (46–69)	75 (53–89)	50 (38–62)	60 (51–69)	58 (43–72)	54 (47–60)	57 (48–65)	59 (46–70)	48 (33–64)
	SupportVectorClassifier	49 (38–61)	50 (30–70)	50 (38–62)	60 (51–69)	54 (39–68)	51 (44–58)	53 (45–62)	62 (49–73)	50 (34–66)
	MultilayerPerceptronClassifier	66 (54–76)	50 (30–70)	74 (62–84)	60 (50–68)	35 (22–51)	46 (39–53)	54 (46–63)	61 (48–72)	41 (27–57)

See the Methods section (*Model training and validation*) for a full description of test sets: combined country, between country, and within country.
Country abbreviations: MOZ—Mozambique, NER—Niger, CIV—Côte d'Ivoire, KEN—Kenya, TZA—Tanzania.
*Note: Model performance was very poor for these models and test sets. These test sets each contained less than 50 communities, highlighting the challenge of model development and evaluation with a limited sample size. In particular, the 'between country' test sets performed poorly in general.

Table S17: Sensitivity (with 95% CI) of each model trained on epidemiologic data alone, by hotspot definition and test set.

Species		*S. haematobium*			*S. mansoni*					
		Combined	Between	Within	Combined	Between			Within	
Test data country			MOZ*	NER		CIV*	KEN	TZA	KEN	TZA*
Prevalence hotspot	EnsembleClassifier	81 (60–92)	40 (20–64)	81 (57–93)	81 (70–89)	38 (18–61)	82 (74–88)	74 (65–82)	76 (59–87)	100 (88–100)
	LogisticRegression	71 (50–86)	93 (70–99)	56 (33–77)	84 (74–91)	75 (51–90)	84 (76–90)	74 (65–82)	79 (62–89)	100 (88–100)
	ElasticNetLogistic	76 (55–89)	40 (20–64)	75 (51–90)	81 (70–89)	38 (18–61)	87 (79–92)	76 (67–83)	76 (59–87)	100 (88–100)
	RandomForestClassifier	90 (71–97)	73 (48–89)	81 (57–93)	75 (63–84)	38 (18–61)	87 (79–92)	75 (66–82)	76 (59–87)	100 (88–100)
	GradientBoostingClassifier	86 (65–95)	100 (80–100)	88 (64–97)	78 (67–86)	50 (28–72)	80 (71–87)	79 (70–86)	67 (50–80)	100 (88–100)
	SupportVectorClassifier	81 (60–92)	40 (20–64)	75 (51–90)	84 (74–91)	44 (23–67)	78 (69–85)	71 (62–79)	73 (56–85)	100 (88–100)
	MultilayerPerceptronClassifier	81 (60–92)	40 (20–64)	88 (64–97)	78 (67–86)	75 (51–90)	83 (75–89)	71 (62–79)	82 (66–91)	93 (77–98)
	EnsembleRegressor	90 (71–97)	100 (80–100)	88 (64–97)	89 (79–95)	44 (23–67)	94 (88–97)	87 (79–92)	85 (69–93)	100 (88–100)
	LinearRegression	90 (71–97)	100 (80–100)	81 (57–93)	86 (75–92)	88 (64–97)	94 (88–97)	86 (78–91)	88 (73–95)	100 (88–100)
	ElasticNetLinear	90 (71–97)	100 (80–100)	81 (57–93)	88 (77–94)	50 (28–72)	95 (89–98)	87 (79–92)	82 (66–91)	100 (88–100)
	RandomForestRegressor	95 (77–99)	100 (80–100)	88 (64–97)	92 (83–97)	75 (51–90)	92 (85–96)	84 (75–90)	76 (59–87)	100 (88–100)
	GradientBoostingRegressor	95 (77–99)	100 (80–100)	88 (64–97)	83 (72–90)	50 (28–72)	93 (86–97)	82 (73–88)	79 (62–89)	100 (88–100)
	SupportVectorRegressor	90 (71–97)	100 (80–100)	81 (57–93)	86 (75–92)	62 (39–82)	87 (79–92)	90 (83–95)	85 (69–93)	100 (88–100)
	MultilayerPerceptronRegressor	86 (65–95)	100 (80–100)	81 (57–93)	91 (81–96)	56 (33–77)	90 (83–95)	82 (73–88)	76 (59–87)	93 (77–98)
Intensity hotspot	EnsembleClassifier	46 (23–71)	73 (48–89)	27 (10–57)	81 (70–89)	38 (18–64)	84 (76–90)	69 (59–77)	78 (61–89)	100 (88–100)
	LogisticRegression	54 (29–77)	87 (62–96)	18 (5–48)	80 (68–88)	62 (36–82)	88 (80–93)	76 (67–83)	75 (58–87)	100 (88–100)
	ElasticNetLogistic	54 (29–77)	87 (62–96)	27 (10–57)	83 (72–91)	38 (18–64)	87 (79–93)	72 (63–80)	78 (61–89)	100 (88–100)
	RandomForestClassifier	69 (42–87)	87 (62–96)	36 (15–65)	78 (66–87)	23 (8–50)	86 (78–92)	76 (67–83)	78 (61–89)	100 (88–100)
	GradientBoostingClassifier	38 (18–64)	87 (62–96)	55 (28–79)	76 (64–85)	38 (18–64)	84 (76–90)	75 (66–82)	81 (65–91)	100 (88–100)
	SupportVectorClassifier	62 (36–82)	87 (62–96)	45 (21–72)	88 (77–94)	54 (29–77)	80 (71–87)	69 (59–77)	75 (58–87)	100 (88–100)
	MultilayerPerceptronClassifier	46 (23–71)	40 (20–64)	45 (21–72)	78 (66–87)	31 (13–58)	73 (63–81)	67 (57–75)	72 (55–84)	89 (72–96)
Persistent hotspot	EnsembleClassifier	0 (0–26)	0 (0–32)	0 (0–26)	27 (16–42)	9 (2–38)	49 (34–64)	18 (11–28)	27 (11–52)	86 (67–95)
	LogisticRegression	9 (2–38)	0 (0–32)	0 (0–26)	29 (18–44)	0 (0–26)	51 (36–66)	0 (0–5)	27 (11–52)	95 (78–99)
	ElasticNetLogistic	0 (0–26)	38 (14–69)	0 (0–26)	27 (16–42)	18 (5–48)	63 (48–76)	13 (7–22)	20 (7–45)	100 (85–100)
	RandomForestClassifier	9 (2–38)	50 (22–78)	0 (0–26)	27 (16–42)	18 (5–48)	54 (39–68)	32 (22–43)	27 (11–52)	86 (67–95)
	GradientBoostingClassifier	18 (5–48)	75 (41–93)	0 (0–26)	22 (12–37)	45 (21–72)	49 (34–64)	18 (11–28)	27 (11–52)	68 (47–84)
	SupportVectorClassifier	0 (0–26)	0 (0–32)	0 (0–26)	27 (16–42)	36 (15–65)	44 (30–59)	20 (13–30)	27 (11–52)	100 (85–100)
	MultilayerPerceptronClassifier	45 (21–72)	0 (0–32)	55 (28–79)	27 (16–42)	27 (10–57)	41 (28–57)	52 (41–63)	33 (15–58)	68 (47–84)

See the Methods section (*Model training and validation*) for a full description of test sets: combined country, between country, and within country.
Country abbreviations: MOZ—Mozambique, NER—Niger, CIV—Côte d'Ivoire, KEN—Kenya, TZA—Tanzania.
*Note: Model performance was very poor for these models and test sets. These test sets each contained less than 50 communities, highlighting the challenge of model development and evaluation with a limited sample size. In particular, the 'between country' test sets performed poorly in general.

Table S18: Specificity (with 95% CI) of each model trained on epidemiologic data alone, by hotspot definition and test set.

Species		*S. haematobium*			*S. mansoni*					
		Combined	Between	Within	Combined	Between			Within	
Test data country			MOZ*	NER		CIV*	KEN	TZA	KEN	TZA*
Prevalence hotspot	EnsembleClassifier	96 (86–99)	80 (38–96)	94 (83–98)	65 (50–78)	91 (73–98)	56 (46–66)	79 (57–91)	92 (76–98)	0 (0–32)
	LogisticRegression	96 (86–99)	60 (23–88)	96 (86–99)	60 (46–74)	17 (7–37)	47 (37–57)	79 (57–91)	81 (62–91)	0 (0–32)
	ElasticNetLogistic	98 (89–100)	100 (57–100)	98 (89–100)	49 (35–63)	91 (73–98)	46 (36–56)	74 (51–88)	85 (66–94)	0 (0–32)
	RandomForestClassifier	94 (83–98)	40 (12–77)	85 (72–93)	77 (62–87)	83 (63–93)	48 (38–58)	79 (57–91)	81 (62–91)	0 (0–32)
	GradientBoostingClassifier	94 (83–98)	60 (23–88)	85 (72–93)	70 (55–81)	87 (68–95)	59 (49–69)	68 (46–85)	85 (66–94)	12 (2–47)
	SupportVectorClassifier	96 (86–99)	80 (38–96)	94 (83–98)	53 (39–67)	65 (45–81)	57 (47–67)	79 (57–91)	92 (76–98)	0 (0–32)
	MultilayerPerceptronClassifier	92 (80–97)	60 (23–88)	83 (70–91)	79 (65–89)	26 (13–46)	56 (46–66)	74 (51–88)	54 (35–71)	38 (14–69)
	EnsembleRegressor	88 (75–94)	0 (0–43)	83 (70–91)	47 (33–61)	74 (54–87)	23 (16–32)	58 (36–77)	62 (43–78)	0 (0–32)
	LinearRegression	90 (78–95)	20 (4–62)	87 (75–94)	47 (33–61)	9 (2–27)	27 (19–37)	58 (36–77)	62 (43–78)	0 (0–32)
	ElasticNetLinear	83 (70–91)	60 (23–88)	85 (72–93)	40 (26–54)	83 (63–93)	30 (22–40)	53 (32–73)	46 (29–65)	0 (0–32)
	RandomForestRegressor	85 (73–93)	20 (4–62)	83 (70–91)	42 (28–57)	43 (26–63)	25 (17–35)	58 (36–77)	62 (43–78)	0 (0–32)
	GradientBoostingRegressor	90 (78–95)	20 (4–62)	81 (67–90)	53 (39–67)	57 (37–74)	31 (23–41)	68 (46–85)	73 (54–86)	0 (0–32)
	SupportVectorRegressor	83 (70–91)	0 (0–43)	72 (58–83)	40 (26–54)	39 (22–59)	29 (21–39)	42 (23–64)	50 (32–68)	12 (2–47)
	MultilayerPerceptronRegressor	92 (80–97)	0 (0–43)	85 (72–93)	56 (41–70)	43 (26–63)	34 (26–44)	58 (36–77)	65 (46–81)	12 (2–47)
Intensity hotspot	EnsembleClassifier	96 (88–99)	80 (38–96)	94 (84–98)	79 (66–88)	96 (81–99)	59 (49–68)	87 (68–95)	93 (77–98)	22 (6–55)
	LogisticRegression	98 (91–100)	60 (23–88)	94 (84–98)	77 (63–87)	42 (26–61)	51 (41–60)	78 (58–90)	78 (59–89)	22 (6–55)
	ElasticNetLogistic	98 (91–100)	60 (23–88)	94 (84–98)	75 (61–85)	100 (87–100)	53 (43–62)	87 (68–95)	93 (77–98)	0 (0–30)
	RandomForestClassifier	98 (91–100)	60 (23–88)	94 (84–98)	85 (73–93)	85 (66–94)	55 (45–64)	87 (68–95)	85 (68–94)	33 (12–65)
	GradientBoostingClassifier	96 (88–99)	60 (23–88)	90 (79–96)	79 (66–88)	88 (71–96)	57 (47–66)	78 (58–90)	74 (55–87)	11 (2–43)
	SupportVectorClassifier	95 (85–98)	80 (38–96)	94 (84–98)	65 (50–77)	73 (54–86)	61 (51–70)	87 (68–95)	93 (77–98)	0 (0–30)
	MultilayerPerceptronClassifier	91 (81–96)	40 (12–77)	90 (79–96)	65 (50–77)	73 (54–86)	71 (61–79)	74 (54–87)	70 (52–84)	44 (19–73)
Persistent hotspot	EnsembleClassifier	100 (94–100)	100 (76–100)	100 (93–100)	95 (87–98)	93 (77–98)	64 (56–71)	91 (79–96)	95 (85–99)	7 (1–31)
	LogisticRegression	98 (91–100)	100 (76–100)	100 (93–100)	86 (76–93)	100 (88–100)	65 (58–72)	100 (92–100)	98 (88–100)	29 (12–55)
	ElasticNetLogistic	100 (94–100)	33 (14–61)	100 (93–100)	92 (83–97)	100 (88–100)	54 (47–62)	91 (79–96)	100 (92–100)	7 (1–31)
	RandomForestClassifier	100 (94–100)	83 (55–95)	100 (93–100)	89 (80–95)	89 (73–96)	56 (49–64)	73 (58–84)	95 (85–99)	21 (8–48)
	GradientBoostingClassifier	98 (91–100)	75 (47–91)	100 (93–100)	98 (92–100)	71 (53–85)	58 (50–66)	95 (85–99)	91 (79–96)	29 (12–55)
	SupportVectorClassifier	98 (91–100)	100 (76–100)	100 (93–100)	94 (85–98)	71 (53–85)	59 (51–66)	86 (73–94)	98 (88–100)	0 (0–22)
	MultilayerPerceptronClassifier	86 (75–93)	100 (76–100)	94 (84–98)	92 (83–97)	43 (27–61)	50 (42–58)	57 (42–70)	89 (76–95)	14 (4–40)

See the Methods section (*Model training and validation*) for a full description of test sets: combined country, between country, and within country.
Country abbreviations: MOZ—Mozambique, NER—Niger, CIV—Côte d'Ivoire, KEN—Kenya, TZA—Tanzania.
*Note: Model performance was very poor for these models and test sets. These test sets each contained less than 50 communities, highlighting the challenge of model development and evaluation with a limited sample size. In particular, the 'between country' test sets performed poorly in general.

Table S19: Accuracy (with 95% CI) of each model trained using a 6:2:2 train-validate-test data split, by hotspot definition and test set.

Species		*S. haematobium*			*S. mansoni*					
		Combined	Between	Within	Combined	Between			Within	
Test data country			MOZ	NER		CIV	KEN	TZA	KEN	TZA
Prevalence hotspot	EnsembleClassifier	87 (74–94)	25 (11–47)	88 (75–95)	85 (74–91)	59 (43–73)	52 (45–59)	84 (76–89)	79 (64–89)	88 (69–96)
	LogisticRegression	74 (60–84)	50 (30–70)	83 (69–92)	75 (63–83)	59 (43–73)	56 (49–63)	75 (66–82)	79 (64–89)	83 (64–93)
	ElasticNetLogistic	87 (74–94)	25 (11–47)	88 (75–95)	83 (73–90)	59 (43–73)	51 (44–58)	85 (77–90)	79 (64–89)	83 (64–93)
	RandomForestClassifier	87 (74–94)	85 (64–95)	90 (78–96)	82 (71–89)	64 (48–77)	56 (49–63)	80 (72–86)	82 (67–91)	83 (64–93)
	GradientBoostingClassifier	80 (67–89)	80 (58–92)	90 (78–96)	75 (63–83)	62 (46–75)	65 (58–71)	69 (60–77)	79 (64–89)	83 (64–93)
	SupportVectorClassifier	80 (67–89)	25 (11–47)	83 (69–92)	82 (71–89)	59 (43–73)	52 (45–59)	84 (76–89)	74 (59–85)	83 (64–93)
	MultilayerPerceptronClassifier	83 (69–91)	25 (11–47)	88 (75–95)	79 (68–87)	56 (41–71)	51 (44–58)	80 (73–87)	79 (64–89)	88 (69–96)
	EnsembleRegressor	80 (67–89)	40 (22–61)	88 (75–95)	86 (76–92)	59 (43–73)	51 (44–58)	85 (77–90)	87 (73–94)	83 (64–93)
	LinearRegression	85 (72–92)	80 (58–92)	83 (69–92)	76 (65–84)	59 (43–73)	63 (56–69)	84 (76–89)	67 (51–79)	79 (60–91)
	ElasticNetLinear	78 (64–88)	75 (53–89)	69 (54–81)	79 (68–87)	59 (43–73)	51 (44–58)	85 (77–90)	77 (62–87)	92 (74–98)
	RandomForestRegressor	80 (67–89)	75 (53–89)	83 (69–92)	80 (70–88)	62 (46–75)	51 (44–58)	85 (77–90)	85 (70–93)	83 (64–93)
	GradientBoostingRegressor	74 (60–84)	75 (53–89)	81 (67–90)	83 (73–90)	59 (43–73)	51 (44–58)	85 (77–90)	82 (67–91)	83 (64–93)
	SupportVectorRegressor	85 (72–92)	75 (53–89)	83 (69–92)	73 (62–82)	56 (41–71)	51 (44–58)	85 (77–90)	77 (62–87)	83 (64–93)
	MultilayerPerceptronRegressor	89 (77–95)	25 (11–47)	71 (56–83)	76 (65–84)	59 (43–73)	51 (44–58)	82 (74–88)	82 (67–91)	75 (55–88)
Intensity hotspot	EnsembleClassifier	85 (72–92)	25 (11–47)	81 (67–90)	85 (74–91)	67 (51–79)	52 (45–59)	85 (78–91)	82 (67–91)	92 (74–98)
	LogisticRegression	83 (69–91)	80 (58–92)	79 (64–88)	76 (65–84)	69 (54–81)	68 (61–74)	71 (62–78)	82 (67–91)	79 (60–91)
	ElasticNetLogistic	83 (69–91)	75 (53–89)	81 (67–90)	79 (68–87)	67 (51–79)	49 (42–56)	81 (74–87)	79 (64–89)	96 (80–99)
	RandomForestClassifier	83 (69–91)	25 (11–47)	76 (61–87)	80 (70–88)	72 (56–83)	63 (56–69)	81 (74–87)	85 (70–93)	79 (60–91)
	GradientBoostingClassifier	78 (64–88)	20 (8–42)	86 (72–93)	80 (70–88)	67 (51–79)	70 (63–76)	72 (64–79)	82 (67–91)	75 (55–88)
	SupportVectorClassifier	74 (60–84)	25 (11–47)	79 (64–88)	87 (78–93)	67 (51–79)	50 (43–57)	85 (77–90)	82 (67–91)	79 (60–91)
	MultilayerPerceptronClassifier	83 (69–91)	25 (11–47)	79 (64–88)	75 (63–83)	67 (51–79)	53 (46–60)	81 (74–87)	82 (67–91)	88 (69–96)
Persistent hotspot	EnsembleClassifier	76 (62–86)	60 (39–78)	81 (67–90)	80 (70–88)	72 (56–83)	24 (18–30)	37 (29–46)	59 (43–73)	71 (51–85)
	LogisticRegression	76 (62–86)	45 (26–66)	71 (56–83)	72 (60–81)	72 (56–83)	21 (16–27)	36 (28–45)	59 (43–73)	83 (64–93)
	ElasticNetLogistic	76 (62–86)	55 (34–74)	79 (64–88)	80 (70–88)	72 (56–83)	22 (17–29)	50 (41–58)	59 (43–73)	71 (51–85)
	RandomForestClassifier	76 (62–86)	60 (39–78)	83 (69–92)	82 (71–89)	72 (56–83)	36 (30–43)	37 (29–46)	59 (43–73)	79 (60–91)
	GradientBoostingClassifier	83 (69–91)	60 (39–78)	79 (64–88)	79 (68–87)	69 (54–81)	40 (33–47)	46 (37–54)	69 (54–81)	67 (47–82)
	SupportVectorClassifier	76 (62–86)	60 (39–78)	88 (75–95)	72 (60–81)	67 (51–79)	21 (16–27)	36 (28–45)	59 (43–73)	79 (60–91)
	MultilayerPerceptronClassifier	80 (67–89)	55 (34–74)	79 (64–88)	59 (48–70)	67 (51–79)	34 (28–41)	55 (46–64)	77 (62–87)	62 (43–79)

See the Methods section (*Model training and validation*) for a full description of test sets: combined country, between country, and within country.
Country abbreviations: MOZ—Mozambique, NER—Niger, CIV—Côte d'Ivoire, KEN—Kenya, TZA—Tanzania.
*Note: These test sets contained under 50 communities in the 6:2:2 split, limiting a meaningful model development and evaluation.

Table S20: Balanced accuracy (mean of sensitivity and specificity, with 95% CI) of each model trained using a 6:2:2 train-validate-test data split, by hotspot definition and test set.

Species		*S. haematobium*			*S. mansoni*					
		Combined	Between	Within	Combined	Between			Within	
Test data country			MOZ	NER		CIV	KEN	TZA	KEN	TZA
Prevalence hotspot	EnsembleClassifier	85 (72–93)	50 (30–70)	83 (69–91)	86 (76–92)	50 (35–65)	51 (44–58)	58 (49–66)	81 (66–90)	82 (63–93)
	LogisticRegression	67 (52–79)	67 (45–83)	82 (67–90)	73 (62–82)	50 (35–65)	55 (48–62)	79 (71–85)	81 (66–90)	50 (31–69)
	ElasticNetLogistic	87 (74–94)	50 (30–70)	83 (69–91)	83 (72–90)	50 (35–65)	50 (43–57)	50 (41–59)	81 (66–90)	80 (60–91)
	RandomForestClassifier	85 (72–93)	70 (48–85)	87 (73–94)	82 (71–89)	56 (41–71)	55 (48–62)	75 (67–82)	84 (70–92)	50 (31–69)
	GradientBoostingClassifier	80 (67–89)	80 (58–92)	87 (73–94)	76 (65–84)	53 (38–68)	64 (57–71)	73 (65–80)	81 (66–90)	80 (60–91)
	SupportVectorClassifier	75 (61–85)	50 (30–70)	75 (60–86)	83 (73–90)	50 (35–65)	51 (44–58)	50 (41–58)	76 (61–87)	50 (31–69)
	MultilayerPerceptronClassifier	82 (69–90)	50 (30–70)	85 (71–93)	78 (67–86)	48 (33–63)	50 (43–57)	58 (50–67)	80 (66–90)	92 (75–98)
	EnsembleRegressor	82 (69–91)	60 (39–78)	87 (74–94)	84 (74–91)	50 (35–65)	50 (43–57)	50 (41–59)	89 (75–95)	50 (31–69)
	LinearRegression	85 (72–93)	60 (39–78)	82 (67–90)	73 (62–82)	50 (35–65)	62 (55–69)	71 (62–78)	65 (49–78)	57 (38–75)
	ElasticNetLinear	79 (65–88)	50 (30–70)	50 (36–64)	76 (65–84)	50 (35–65)	50 (43–57)	50 (41–59)	77 (62–87)	95 (79–99)
	RandomForestRegressor	84 (71–92)	50 (30–70)	86 (72–93)	79 (68–86)	59 (43–73)	50 (43–57)	50 (41–59)	85 (71–93)	50 (31–69)
	GradientBoostingRegressor	75 (61–86)	50 (30–70)	82 (68–91)	82 (71–89)	50 (35–65)	50 (43–57)	50 (41–59)	83 (68–92)	50 (31–69)
	SupportVectorRegressor	89 (76–95)	63 (42–81)	84 (70–92)	69 (57–78)	51 (36–66)	50 (43–57)	50 (41–59)	76 (61–87)	50 (31–69)
	MultilayerPerceptronRegressor	88 (76–95)	50 (30–70)	67 (51–79)	73 (62–82)	50 (35–65)	50 (43–57)	57 (48–66)	84 (70–92)	65 (45–81)
Intensity hotspot	EnsembleClassifier	74 (60–84)	50 (30–70)	60 (45–73)	85 (75–91)	50 (35–65)	54 (47–61)	83 (75–88)	83 (68–92)	87 (69–96)
	LogisticRegression	72 (58–83)	80 (58–92)	62 (47–75)	76 (65–84)	58 (42–72)	68 (62–74)	77 (69–84)	83 (68–92)	50 (31–69)
	ElasticNetLogistic	72 (58–83)	57 (36–76)	68 (53–80)	79 (68–87)	50 (35–65)	50 (44–57)	50 (41–59)	81 (66–90)	97 (82–100)
	RandomForestClassifier	72 (58–83)	50 (30–70)	53 (38–67)	80 (70–88)	58 (42–72)	64 (57–70)	83 (76–89)	86 (72–93)	50 (31–69)
	GradientBoostingClassifier	74 (60–84)	33 (17–55)	71 (56–82)	81 (70–88)	50 (35–65)	71 (64–76)	76 (68–83)	83 (69–92)	77 (57–89)
	SupportVectorClassifier	67 (53–79)	50 (30–70)	50 (36–64)	88 (78–93)	50 (35–65)	52 (45–59)	82 (74–88)	83 (68–92)	50 (31–69)
	MultilayerPerceptronClassifier	77 (63–86)	50 (30–70)	70 (55–82)	74 (63–83)	50 (35–65)	54 (47–61)	60 (51–68)	83 (68–92)	85 (66–94)
Persistent hotspot	EnsembleClassifier	50 (36–64)	50 (30–70)	60 (45–73)	77 (66–85)	50 (35–65)	51 (44–58)	51 (43–60)	50 (35–65)	66 (46–81)
	LogisticRegression	50 (36–64)	44 (25–65)	49 (35–64)	65 (53–75)	50 (35–65)	50 (43–57)	50 (41–59)	50 (35–65)	78 (58–90)
	ElasticNetLogistic	59 (45–72)	46 (26–67)	58 (43–72)	77 (66–86)	50 (35–65)	51 (44–58)	56 (47–65)	50 (35–65)	66 (46–81)
	RandomForestClassifier	50 (36–64)	50 (30–70)	65 (50–78)	77 (66–85)	50 (35–65)	52 (45–58)	51 (43–60)	50 (35–65)	72 (52–86)
	GradientBoostingClassifier	70 (56–81)	50 (30–70)	50 (36–64)	74 (62–82)	48 (33–63)	52 (45–59)	56 (47–65)	62 (47–76)	62 (43–79)
	SupportVectorClassifier	50 (36–64)	50 (30–70)	76 (62–87)	70 (59–79)	49 (34–64)	50 (43–57)	50 (41–59)	50 (35–65)	72 (52–86)
	MultilayerPerceptronClassifier	68 (54–80)	48 (28–68)	66 (51–79)	58 (46–68)	46 (32–62)	53 (46–60)	47 (38–55)	74 (58–85)	56 (37–74)

See the Methods section (*Model training and validation*) for a full description of test sets: combined country, between country, and within country.
Country abbreviations: MOZ—Mozambique, NER—Niger, CIV—Côte d'Ivoire, KEN—Kenya, TZA—Tanzania.
*Note: These test sets contained under 50 communities in the 6:2:2 split, limiting a meaningful model development and evaluation.

Table S21: Sensitivity (with 95% CI) of each model trained using a 6:2:2 train-validate-test data split, by hotspot definition and test set.

Species		*S. haematobium*			*S. mansoni*					
		Combined	Between	Within	Combined	Between			Within	
Test data country			MOZ	NER		CIV	KEN	TZA	KEN	TZA
Prevalence hotspot	EnsembleClassifier	80 (55–93)	0 (0–20)	69 (42–87)	79 (64–88)	0 (0–19)	99 (95–100)	95 (89–98)	68 (47–84)	90 (70–97)
	LogisticRegression	47 (25–70)	33 (15–58)	77 (50–92)	81 (67–90)	0 (0–19)	93 (86–97)	73 (64–81)	68 (47–84)	100 (84–100)
	ElasticNetLogistic	87 (62–96)	0 (0–20)	69 (42–87)	86 (72–93)	0 (0–19)	99 (95–100)	100 (96–100)	68 (47–84)	85 (64–95)
	RandomForestClassifier	80 (55–93)	100 (80–100)	77 (50–92)	81 (67–90)	12 (3–36)	98 (93–99)	82 (73–88)	68 (47–84)	100 (84–100)
	GradientBoostingClassifier	80 (55–93)	80 (55–93)	77 (50–92)	69 (54–81)	6 (1–28)	90 (83–95)	67 (58–76)	68 (47–84)	85 (64–95)
	SupportVectorClassifier	60 (36–80)	0 (0–20)	54 (29–77)	76 (61–87)	0 (0–19)	97 (92–99)	99 (95–100)	64 (43–80)	100 (84–100)
	MultilayerPerceptronClassifier	80 (55–93)	0 (0–20)	77 (50–92)	83 (69–92)	0 (0–19)	100 (96–100)	90 (83–95)	73 (52–87)	85 (64–95)
	EnsembleRegressor	87 (62–96)	20 (7–45)	85 (58–96)	95 (84–99)	0 (0–19)	100 (96–100)	100 (96–100)	77 (57–90)	100 (84–100)
	LinearRegression	87 (62–96)	100 (80–100)	77 (50–92)	88 (75–95)	0 (0–19)	95 (89–98)	89 (82–94)	77 (57–90)	90 (70–97)
	ElasticNetLinear	80 (55–93)	100 (80–100)	0 (-0–23)	93 (81–98)	0 (0–19)	100 (96–100)	100 (96–100)	77 (57–90)	90 (70–97)
	RandomForestRegressor	93 (70–99)	100 (80–100)	92 (67–99)	88 (75–95)	44 (23–67)	100 (96–100)	100 (96–100)	82 (61–93)	100 (84–100)
	GradientBoostingRegressor	80 (55–93)	100 (80–100)	85 (58–96)	88 (75–95)	0 (0–19)	100 (96–100)	100 (96–100)	77 (57–90)	100 (84–100)
	SupportVectorRegressor	100 (80–100)	87 (62–96)	85 (58–96)	93 (81–98)	19 (7–43)	99 (95–100)	100 (96–100)	82 (61–93)	100 (84–100)
	MultilayerPerceptronRegressor	87 (62–96)	0 (0–20)	54 (29–77)	88 (75–95)	0 (0–19)	100 (96–100)	93 (87–97)	68 (47–84)	80 (58–92)
Intensity hotspot	EnsembleClassifier	56 (27–81)	0 (0–20)	22 (6–55)	79 (64–89)	0 (-0–23)	99 (94–100)	87 (79–92)	71 (50–86)	95 (75–99)
	LogisticRegression	56 (27–81)	80 (55–93)	33 (12–65)	76 (61–87)	23 (8–50)	91 (83–95)	67 (57–75)	71 (50–86)	100 (83–100)
	ElasticNetLogistic	56 (27–81)	93 (70–99)	44 (19–73)	82 (67–91)	0 (-0–23)	100 (96–100)	100 (96–100)	67 (45–83)	95 (75–99)
	RandomForestClassifier	56 (27–81)	0 (0–20)	11 (2–43)	79 (64–89)	15 (4–42)	89 (82–94)	80 (71–87)	71 (50–86)	100 (83–100)
	GradientBoostingClassifier	67 (35–88)	7 (1–30)	44 (19–73)	76 (61–87)	0 (-0–23)	84 (76–90)	70 (60–78)	67 (45–83)	74 (51–88)
	SupportVectorClassifier	56 (27–81)	0 (0–20)	0 (0–30)	82 (67–91)	0 (-0–23)	100 (96–100)	86 (78–91)	71 (50–86)	100 (83–100)
	MultilayerPerceptronClassifier	67 (35–88)	0 (0–20)	56 (27–81)	82 (67–91)	0 (-0–23)	99 (94–100)	94 (88–97)	71 (50–86)	89 (69–97)
Persistent hotspot	EnsembleClassifier	0 (0–26)	0 (0–32)	22 (6–55)	64 (45–80)	0 (0–26)	98 (87–100)	3 (1–9)	0 (0–19)	81 (57–93)
	LogisticRegression	0 (0–26)	38 (14–69)	11 (2–43)	40 (23–59)	0 (0–26)	100 (91–100)	0 (0–5)	0 (0–19)	94 (72–99)
	ElasticNetLogistic	27 (10–57)	0 (0–32)	22 (6–55)	68 (48–83)	0 (0–26)	100 (91–100)	33 (24–44)	0 (0–19)	81 (57–93)
	RandomForestClassifier	0 (0–26)	0 (0–32)	33 (12–65)	60 (41–77)	0 (0–26)	78 (63–88)	3 (1–9)	0 (0–19)	94 (72–99)
	GradientBoostingClassifier	45 (21–72)	0 (0–32)	0 (0–30)	56 (37–73)	0 (0–26)	73 (58–84)	19 (12–29)	25 (10–49)	75 (51–90)
	SupportVectorClassifier	0 (0–26)	0 (0–32)	56 (27–81)	64 (45–80)	9 (2–38)	100 (91–100)	0 (0–5)	0 (0–19)	94 (72–99)
	MultilayerPerceptronClassifier	45 (21–72)	12 (2–47)	44 (19–73)	52 (33–70)	0 (0–26)	85 (72–93)	77 (67–85)	56 (33–77)	75 (51–90)

See the Methods section (*Model training and validation*) for a full description of test sets: combined country, between country, and within country.
Country abbreviations: MOZ—Mozambique, NER—Niger, CIV—Côte d'Ivoire, KEN—Kenya, TZA—Tanzania.
*Note: These test sets contained under 50 communities in the 6:2:2 split, limiting a meaningful model development and evaluation.

Table S22: Specificity (with 95% CI) of each model trained using a 6:2:2 train-validate-test data split, by hotspot definition and test set.

Species		*S. haematobium*			*S. mansoni*					
		Combined	Between	Within	Combined	Between			Within	
Test data country			MOZ	NER		CIV	KEN	TZA	KEN	TZA
Prevalence hotspot	EnsembleClassifier	90 (75–97)	100 (57–100)	97 (83–99)	93 (78–98)	100 (86–100)	3 (1–9)	21 (9–43)	94 (73–99)	75 (30–95)
	LogisticRegression	87 (71–95)	100 (57–100)	86 (69–95)	66 (47–80)	100 (86–100)	17 (11–25)	84 (62–94)	94 (73–99)	0 (0–49)
	ElasticNetLogistic	87 (71–95)	100 (57–100)	97 (83–99)	79 (62–90)	100 (86–100)	1 (0–6)	0 (0–17)	94 (73–99)	75 (30–95)
	RandomForestClassifier	90 (75–97)	40 (12–77)	97 (83–99)	83 (65–92)	100 (86–100)	11 (7–19)	68 (46–85)	100 (82–100)	0 (0–49)
	GradientBoostingClassifier	81 (64–91)	80 (38–96)	97 (83–99)	83 (65–92)	100 (86–100)	39 (29–49)	79 (57–91)	94 (73–99)	75 (30–95)
	SupportVectorClassifier	90 (75–97)	100 (57–100)	97 (83–99)	90 (74–96)	100 (86–100)	5 (2–12)	0 (0–17)	88 (66–97)	0 (0–49)
	MultilayerPerceptronClassifier	84 (67–93)	100 (57–100)	93 (78–98)	72 (54–85)	96 (79–99)	0 (0–4)	26 (12–49)	88 (66–97)	100 (51–100)
	EnsembleRegressor	77 (60–89)	100 (57–100)	90 (74–96)	72 (54–85)	100 (86–100)	0 (0–4)	0 (0–17)	100 (82–100)	0 (0–49)
	LinearRegression	84 (67–93)	20 (4–62)	86 (69–95)	59 (41–74)	100 (86–100)	29 (21–39)	53 (32–73)	53 (31–74)	25 (5–70)
	ElasticNetLinear	77 (60–89)	0 (0–43)	100 (88–100)	59 (41–74)	100 (86–100)	0 (0–4)	0 (0–17)	76 (53–90)	100 (51–100)
	RandomForestRegressor	74 (57–86)	0 (0–43)	79 (62–90)	69 (51–83)	74 (54–87)	0 (0–4)	0 (0–17)	88 (66–97)	0 (0–49)
	GradientBoostingRegressor	71 (53–84)	0 (0–43)	79 (62–90)	76 (58–88)	100 (86–100)	0 (0–4)	0 (0–17)	88 (66–97)	0 (0–49)
	SupportVectorRegressor	77 (60–89)	40 (12–77)	83 (65–92)	45 (28–62)	83 (63–93)	1 (0–6)	0 (0–17)	71 (47–87)	0 (0–49)
	MultilayerPerceptronRegressor	90 (75–97)	100 (57–100)	79 (62–90)	59 (41–74)	100 (86–100)	0 (0–4)	21 (9–43)	100 (82–100)	50 (15–85)
Intensity hotspot	EnsembleClassifier	92 (79–97)	100 (57–100)	97 (85–99)	91 (76–97)	100 (87–100)	9 (5–16)	78 (58–90)	94 (74–99)	80 (38–96)
	LogisticRegression	89 (75–96)	80 (38–96)	91 (76–97)	76 (59–87)	92 (76–98)	46 (37–56)	87 (68–95)	94 (74–99)	0 (0–43)
	ElasticNetLogistic	89 (75–96)	20 (4–62)	91 (76–97)	76 (59–87)	100 (87–100)	1 (0–5)	0 (0–14)	94 (74–99)	100 (57–100)
	RandomForestClassifier	89 (75–96)	100 (57–100)	94 (80–98)	82 (66–91)	100 (87–100)	38 (29–48)	87 (68–95)	100 (82–100)	0 (0–43)
	GradientBoostingClassifier	81 (66–91)	60 (23–88)	97 (85–99)	85 (69–93)	100 (87–100)	57 (47–66)	83 (63–93)	100 (82–100)	80 (38–96)
	SupportVectorClassifier	78 (63–89)	100 (57–100)	100 (90–100)	94 (80–98)	100 (87–100)	4 (2–10)	78 (58–90)	94 (74–99)	0 (0–43)
	MultilayerPerceptronClassifier	86 (72–94)	100 (57–100)	85 (69–93)	67 (50–80)	100 (87–100)	10 (5–17)	26 (13–46)	94 (74–99)	80 (38–96)
Persistent hotspot	EnsembleClassifier	100 (90–100)	100 (76–100)	97 (85–99)	89 (77–95)	100 (88–100)	4 (2–9)	100 (92–100)	100 (86–100)	50 (22–78)
	LogisticRegression	100 (90–100)	50 (25–75)	88 (73–95)	89 (77–95)	100 (88–100)	0 (-0–2)	100 (92–100)	100 (86–100)	62 (31–86)
	ElasticNetLogistic	91 (78–97)	92 (65–99)	94 (80–98)	87 (74–94)	100 (88–100)	2 (1–6)	80 (65–89)	100 (86–100)	50 (22–78)
	RandomForestClassifier	100 (90–100)	100 (76–100)	97 (85–99)	93 (82–98)	100 (88–100)	25 (19–32)	100 (92–100)	100 (86–100)	50 (22–78)
	GradientBoostingClassifier	94 (81–98)	100 (76–100)	100 (90–100)	91 (80–97)	96 (82–99)	31 (24–38)	93 (82–98)	100 (86–100)	50 (22–78)
	SupportVectorClassifier	100 (90–100)	100 (76–100)	97 (85–99)	76 (62–86)	89 (73–96)	0 (-0–2)	100 (92–100)	100 (86–100)	50 (22–78)
	MultilayerPerceptronClassifier	91 (78–97)	83 (55–95)	88 (73–95)	63 (49–75)	93 (77–98)	21 (15–28)	16 (8–29)	91 (73–98)	38 (14–69)

See the Methods section (*Model training and validation*) for a full description of test sets: combined country, between country, and within country.
Country abbreviations: MOZ—Mozambique, NER—Niger, CIV—Côte d'Ivoire, KEN—Kenya, TZA—Tanzania.
*Note: These test sets contained under 50 communities in the 6:2:2 split, limiting a meaningful model development and evaluation.

Table S23: Accuracy (with 95% CI) of each model trained on the epidemiologic and secondary data sets, by hotspot definition and test set, with prevalence variables corrected for estimated test sensitivity.

Species		*S. haematobium*			*S. mansoni*					
		Combined	Between	Within	Combined	Between			Within	
Test data country			MOZ*	NER		CIV*	KEN	TZA	KEN	TZA*
Prevalence hotspot	EnsembleClassifier	80 (69–88)	—	78 (66–86)	82 (74–88)	33 (21–49)	77 (70–82)	95 (90–98)	85 (73–92)	89 (75–96)
	LogisticRegression	71 (59–80)	—	76 (64–85)	82 (74–88)	67 (51–79)	78 (71–83)	95 (90–98)	80 (68–88)	89 (75–96)
	ElasticNetLogistic	80 (69–88)	—	76 (64–85)	82 (74–88)	33 (21–49)	77 (71–82)	95 (90–98)	81 (70–89)	89 (75–96)
	RandomForestClassifier	80 (69–88)	—	76 (64–85)	81 (73–88)	64 (48–77)	77 (71–82)	95 (90–98)	85 (73–92)	89 (75–96)
	GradientBoostingClassifier	78 (67–86)	—	76 (64–85)	82 (74–88)	64 (48–77)	77 (70–82)	91 (85–95)	86 (75–93)	92 (78–97)
	SupportVectorClassifier	78 (67–86)	—	76 (64–85)	81 (73–88)	33 (21–49)	77 (71–82)	95 (90–98)	83 (72–91)	92 (78–97)
	MultilayerPerceptronClassifier	83 (72–90)	—	79 (68–88)	79 (71–86)	41 (27–57)	77 (71–82)	94 (89–97)	75 (62–84)	86 (71–94)
	EnsembleRegressor	81 (70–89)	100 (84–100)	76 (64–85)	82 (74–88)	49 (34–64)	77 (71–82)	95 (90–98)	75 (62–84)	92 (78–97)
	LinearRegression	72 (61–82)	90 (70–97)	71 (59–81)	79 (70–85)	67 (51–79)	75 (69–81)	93 (88–97)	78 (66–87)	92 (78–97)
	ElasticNetLinear	72 (61–82)	100 (84–100)	68 (56–78)	84 (76–90)	69 (54–81)	77 (71–82)	95 (90–98)	75 (62–84)	92 (78–97)
	RandomForestRegressor	86 (75–92)	100 (84–100)	78 (66–86)	83 (75–89)	67 (51–79)	77 (71–82)	96 (91–98)	85 (73–92)	92 (78–97)
	GradientBoostingRegressor	81 (70–89)	100 (84–100)	75 (63–84)	82 (74–88)	67 (51–79)	77 (71–82)	95 (90–98)	80 (68–88)	92 (78–97)
	SupportVectorRegressor	75 (64–84)	0 (0–16)	75 (63–84)	80 (72–87)	33 (21–49)	77 (71–82)	94 (89–97)	80 (68–88)	92 (78–97)
	MultilayerPerceptronRegressor	80 (69–88)	95 (76–99)	70 (58–80)	82 (74–88)	36 (23–52)	62 (55–68)	93 (88–97)	75 (62–84)	92 (78–97)
Intensity hotspot	EnsembleClassifier	91 (82–96)	25 (11–47)	84 (73–91)	77 (68–84)	54 (39–68)	63 (57–70)	88 (81–92)	76 (64–85)	83 (68–92)
	LogisticRegression	91 (82–96)	40 (22–61)	87 (77–93)	67 (58–75)	56 (41–71)	63 (56–69)	78 (70–84)	63 (50–74)	83 (68–92)
	ElasticNetLogistic	91 (82–96)	80 (58–92)	83 (71–90)	67 (58–75)	54 (39–68)	61 (54–68)	83 (75–89)	71 (59–81)	83 (68–92)
	RandomForestClassifier	91 (82–96)	30 (15–52)	87 (77–93)	74 (65–81)	69 (54–81)	65 (58–71)	84 (76–89)	75 (62–84)	83 (68–92)
	GradientBoostingClassifier	87 (77–93)	80 (58–92)	84 (73–91)	79 (70–85)	54 (39–68)	61 (54–68)	85 (78–91)	76 (64–85)	83 (68–92)
	SupportVectorClassifier	88 (79–94)	20 (8–42)	87 (77–93)	67 (58–75)	54 (39–68)	63 (56–69)	86 (79–91)	68 (55–78)	83 (68–92)
	MultilayerPerceptronClassifier	87 (77–93)	20 (8–42)	86 (75–92)	72 (63–80)	46 (32–61)	62 (55–68)	87 (80–92)	66 (53–77)	83 (68–92)
Persistent hotspot	EnsembleClassifier	68 (56–78)	35 (18–57)	63 (51–74)	75 (66–82)	59 (43–73)	35 (29–42)	67 (59–75)	75 (62–84)	64 (48–78)
	LogisticRegression	65 (53–75)	40 (22–61)	62 (50–73)	67 (58–75)	59 (43–73)	29 (24–36)	49 (40–58)	68 (55–78)	72 (56–84)
	ElasticNetLogistic	65 (53–75)	60 (39–78)	62 (50–73)	67 (58–75)	59 (43–73)	29 (24–36)	67 (59–75)	68 (55–78)	67 (50–80)
	RandomForestClassifier	70 (58–79)	40 (22–61)	65 (53–76)	75 (66–82)	67 (51–79)	35 (28–41)	65 (56–73)	78 (66–87)	69 (53–82)
	GradientBoostingClassifier	70 (58–79)	35 (18–57)	63 (51–74)	71 (62–79)	54 (39–68)	52 (45–59)	62 (53–70)	76 (64–85)	61 (45–75)
	SupportVectorClassifier	57 (45–68)	40 (22–61)	67 (54–77)	74 (65–81)	59 (43–73)	35 (29–42)	63 (54–71)	69 (57–80)	64 (48–78)
	MultilayerPerceptronClassifier	65 (53–75)	60 (39–78)	68 (56–78)	72 (63–80)	62 (46–75)	36 (30–43)	73 (65–80)	69 (57–80)	72 (56–84)

See the Methods section (*Model training and validation*) for a full description of test sets: combined country, between country, and within country.

The adjustment for imperfect sensitivity has some limitations given the SCORE study includes communities with baseline high prevalence of infection, including an inability to adjust communities with very high baseline prevalence (given lack of data to inform this estimate) and a less balanced training and test set. The analysis was not possible for the prevalence hotspot classifiers in Mozambique because all communities were prevalence hotspots after correction for test sensitivity.

*The between country test set (Mozambique) for *S. haematobium*, between country test set (Côte d'Ivoire) for *S. mansoni*, and within country test set (Tanzania) for *S. mansoni* each contained under 50 communities.

Table S24: Balanced accuracy (mean of sensitivity and specificity, with 95% CI) of each model trained on the epidemiologic and secondary data sets, by hotspot definition and test set, with prevalence variables corrected for estimated test sensitivity.

Species		*S. haematobium*			*S. mansoni*					
		Combined	Between	Within	Combined	Between			Within	
Test data country			MOZ*	NER		CIV*	KEN	TZA	KEN	TZA*
Prevalence hotspot	EnsembleClassifier	80 (70–88)	—	76 (64–85)	52 (43–61)	50 (35–65)	50 (43–57)	50 (41–59)	63 (50–74)	48 (33–64)
	LogisticRegression	73 (61–82)	—	75 (63–84)	50 (41–59)	54 (39–68)	51 (44–58)	50 (41–59)	56 (43–68)	48 (33–64)
	ElasticNetLogistic	79 (68–87)	—	75 (63–84)	50 (41–59)	50 (35–65)	50 (43–57)	50 (41–59)	61 (48–72)	48 (33–64)
	RandomForestClassifier	80 (70–88)	—	75 (63–84)	54 (44–63)	48 (33–63)	50 (43–57)	50 (41–59)	59 (46–71)	48 (33–64)
	GradientBoostingClassifier	76 (65–85)	—	75 (63–84)	50 (41–59)	56 (40–70)	61 (54–67)	64 (55–72)	72 (59–82)	50 (34–66)
	SupportVectorClassifier	78 (67–86)	—	75 (63–84)	49 (40–59)	50 (35–65)	50 (43–57)	50 (41–59)	50 (38–62)	50 (34–66)
	MultilayerPerceptronClassifier	85 (74–91)	—	78 (67–87)	67 (57–75)	54 (39–68)	50 (43–57)	50 (41–58)	53 (40–65)	47 (32–63)
	EnsembleRegressor	78 (67–86)	—	74 (62–83)	50 (41–59)	62 (46–75)	51 (44–58)	50 (41–59)	49 (37–61)	50 (34–66)
	LinearRegression	66 (55–76)	—	68 (56–78)	52 (42–61)	50 (35–65)	49 (42–56)	57 (48–65)	59 (46–71)	50 (34–66)
	ElasticNetLinear	68 (57–78)	—	65 (53–76)	59 (50–68)	67 (52–80)	50 (43–57)	58 (49–66)	57 (44–69)	50 (34–66)
	RandomForestRegressor	79 (68–87)	—	75 (63–84)	57 (47–66)	50 (35–65)	50 (43–57)	58 (49–67)	55 (42–67)	50 (34–66)
	GradientBoostingRegressor	79 (68–87)	—	72 (60–82)	50 (41–59)	50 (35–65)	50 (43–57)	50 (41–59)	60 (47–71)	50 (34–66)
	SupportVectorRegressor	73 (61–82)	—	74 (62–83)	63 (54–72)	50 (35–65)	50 (43–57)	50 (41–58)	48 (36–60)	50 (34–66)
	MultilayerPerceptronRegressor	78 (67–86)	—	68 (56–78)	50 (41–59)	52 (37–67)	58 (51–65)	49 (40–58)	53 (40–65)	50 (34–66)
Intensity hotspot	EnsembleClassifier	83 (72–90)	53 (33–73)	65 (52–75)	71 (62–79)	50 (35–65)	53 (46–60)	66 (58–74)	74 (62–84)	57 (41–71)
	LogisticRegression	80 (69–88)	62 (41–80)	76 (64–85)	54 (45–64)	53 (38–67)	53 (46–60)	74 (66–81)	55 (42–67)	57 (41–71)
	ElasticNetLogistic	83 (72–90)	69 (47–85)	64 (51–74)	50 (41–59)	50 (35–65)	50 (43–57)	58 (49–67)	64 (51–75)	57 (41–71)
	RandomForestClassifier	83 (72–90)	47 (27–67)	73 (61–82)	68 (59–76)	67 (51–80)	55 (48–62)	69 (61–77)	72 (59–81)	50 (34–66)
	GradientBoostingClassifier	89 (79–94)	69 (47–85)	71 (59–81)	74 (65–81)	50 (35–65)	50 (43–57)	68 (59–75)	77 (65–86)	57 (41–71)
	SupportVectorClassifier	84 (74–91)	50 (30–70)	73 (61–82)	50 (41–59)	50 (35–65)	53 (46–60)	58 (49–66)	64 (51–75)	57 (41–71)
	MultilayerPerceptronClassifier	83 (73–90)	50 (30–70)	75 (63–84)	70 (60–78)	44 (30–59)	51 (44–58)	50 (41–59)	63 (50–74)	57 (41–71)
Persistent hotspot	EnsembleClassifier	63 (51–73)	44 (25–65)	57 (45–69)	74 (65–81)	50 (35–65)	53 (46–60)	58 (49–66)	70 (58–80)	54 (38–69)
	LogisticRegression	59 (47–70)	48 (28–68)	62 (50–73)	67 (58–75)	50 (35–65)	50 (43–57)	61 (53–69)	61 (48–72)	60 (44–75)
	ElasticNetLogistic	60 (48–71)	50 (30–70)	59 (47–71)	67 (58–75)	50 (35–65)	50 (43–57)	48 (39–56)	62 (50–74)	50 (34–66)
	RandomForestClassifier	64 (52–74)	50 (30–70)	63 (51–74)	74 (65–82)	60 (45–74)	49 (42–56)	64 (55–72)	73 (60–82)	56 (40–71)
	GradientBoostingClassifier	68 (56–78)	44 (25–65)	63 (51–74)	71 (62–79)	49 (35–64)	57 (50–64)	69 (60–77)	71 (59–81)	52 (36–67)
	SupportVectorClassifier	51 (40–62)	50 (30–70)	61 (49–72)	73 (64–81)	50 (35–65)	53 (46–60)	55 (47–64)	65 (52–76)	58 (42–73)
	MultilayerPerceptronClassifier	62 (50–72)	58 (37–77)	62 (50–73)	72 (63–79)	53 (38–68)	52 (45–59)	51 (43–60)	68 (55–78)	65 (48–78)

See the Methods section (*Model training and validation*) for a full description of test sets: combined country, between country, and within country.
The adjustment for imperfect sensitivity has some limitations given the SCORE study includes communities with baseline high prevalence of infection, including an inability to adjust communities with very high baseline prevalence (given lack of data to inform this estimate) and a less balanced training and test set. The analysis was not possible for the prevalence hotspot classifiers in Mozambique because all communities were prevalence hotspots after correction for test sensitivity.
*The between country test set (Mozambique) for *S. haematobium*, between country test set (Côte d'Ivoire) for *S. mansoni*, and within country test set (Tanzania) for *S. mansoni* each contained under 50 communities.

Table S25: Sensitivity (with 95% CI) of each model trained on the epidemiologic and secondary data sets, by hotspot definition and test set, with prevalence variables corrected for estimated test sensitivity.

Species		*S. haematobium*			*S. mansoni*					
		Combined	Between	Within	Combined	Between			Within	
Test data country			MOZ*	NER		CIV*	KEN	TZA	KEN	TZA*
Prevalence hotspot	EnsembleClassifier	78 (64–88)	—	91 (78–97)	99 (94–100)	0 (0–13)	99 (96–100)	100 (97–100)	96 (86–99)	97 (85–99)
	LogisticRegression	67 (53–79)	—	89 (74–95)	100 (96–100)	92 (76–98)	100 (98–100)	100 (97–100)	92 (81–97)	97 (85–99)
	ElasticNetLogistic	80 (67–89)	—	89 (74–95)	100 (96–100)	0 (0–13)	100 (98–100)	100 (97–100)	92 (81–97)	97 (85–99)
	RandomForestClassifier	78 (64–88)	—	89 (74–95)	97 (90–99)	96 (81–99)	100 (98–100)	100 (97–100)	98 (89–100)	97 (85–99)
	GradientBoostingClassifier	83 (69–91)	—	89 (74–95)	100 (96–100)	81 (62–91)	90 (84–94)	94 (88–97)	94 (83–98)	100 (90–100)
	SupportVectorClassifier	78 (64–88)	—	89 (74–95)	99 (94–100)	0 (0–13)	100 (98–100)	100 (97–100)	100 (93–100)	100 (90–100)
	MultilayerPerceptronClassifier	78 (64–88)	—	89 (74–95)	86 (78–92)	15 (6–34)	100 (98–100)	99 (95–100)	86 (73–93)	94 (80–98)
	EnsembleRegressor	87 (74–94)	100 (84–100)	94 (81–98)	100 (96–100)	23 (11–42)	99 (96–100)	100 (97–100)	88 (76–94)	100 (90–100)
	LinearRegression	85 (72–92)	90 (70–97)	100 (90–100)	93 (86–97)	100 (87–100)	97 (93–99)	97 (93–99)	88 (76–94)	100 (90–100)
	ElasticNetLinear	80 (67–89)	100 (84–100)	94 (81–98)	98 (92–99)	73 (54–86)	100 (98–100)	99 (95–100)	84 (71–91)	100 (90–100)
	RandomForestRegressor	98 (89–100)	100 (84–100)	100 (90–100)	98 (92–99)	100 (87–100)	100 (98–100)	100 (97–100)	100 (93–100)	100 (90–100)
	GradientBoostingRegressor	85 (72–92)	100 (84–100)	94 (81–98)	100 (96–100)	100 (87–100)	100 (98–100)	100 (97–100)	90 (78–96)	100 (90–100)
	SupportVectorRegressor	80 (67–89)	0 (0–16)	83 (67–92)	90 (82–95)	0 (0–13)	100 (98–100)	99 (95–100)	96 (86–99)	100 (90–100)
	MultilayerPerceptronRegressor	83 (69–91)	95 (76–99)	83 (67–92)	100 (96–100)	4 (1–19)	65 (57–72)	98 (94–100)	86 (73–93)	100 (90–100)
Intensity hotspot	EnsembleClassifier	69 (42–87)	6 (1–28)	33 (14–61)	88 (78–93)	0 (0–18)	98 (94–100)	95 (90–98)	80 (65–90)	97 (83–99)
	LogisticRegression	62 (36–82)	25 (10–49)	58 (32–81)	92 (83–96)	6 (1–26)	98 (93–99)	79 (71–86)	78 (62–88)	97 (83–99)
	ElasticNetLogistic	69 (42–87)	88 (64–97)	33 (14–61)	100 (95–100)	0 (0–18)	100 (97–100)	92 (85–96)	85 (71–93)	97 (83–99)
	RandomForestClassifier	69 (42–87)	19 (7–43)	50 (25–75)	85 (75–91)	39 (20–61)	98 (93–99)	89 (81–93)	80 (65–90)	100 (89–100)
	GradientBoostingClassifier	92 (67–99)	88 (64–97)	50 (25–75)	88 (78–93)	0 (0–18)	100 (97–100)	92 (85–96)	75 (60–86)	97 (83–99)
	SupportVectorClassifier	77 (50–92)	0 (0–19)	50 (25–75)	100 (95–100)	0 (0–18)	95 (90–98)	96 (91–99)	75 (60–86)	97 (83–99)
	MultilayerPerceptronClassifier	77 (50–92)	0 (0–19)	58 (32–81)	76 (65–85)	17 (6–39)	98 (94–100)	100 (97–100)	72 (57–84)	97 (83–99)
Persistent hotspot	EnsembleClassifier	37 (22–56)	0 (0–24)	42 (23–64)	61 (47–73)	0 (0–19)	97 (88–99)	78 (68–85)	58 (36–77)	83 (64–93)
	LogisticRegression	30 (16–48)	8 (1–35)	63 (41–81)	65 (51–76)	0 (0–19)	100 (94–100)	35 (26–45)	42 (23–64)	96 (80–99)
	ElasticNetLogistic	37 (22–56)	100 (76–100)	53 (32–73)	59 (45–71)	0 (0–19)	100 (94–100)	89 (81–94)	47 (27–68)	100 (86–100)
	RandomForestClassifier	37 (22–56)	0 (0–24)	58 (36–77)	69 (55–80)	25 (10–49)	84 (73–92)	66 (56–75)	58 (36–77)	96 (80–99)
	GradientBoostingClassifier	59 (41–75)	0 (0–24)	63 (41–81)	78 (65–88)	25 (10–49)	71 (58–81)	54 (44–64)	58 (36–77)	79 (60–91)
	SupportVectorClassifier	26 (13–45)	0 (0–24)	47 (27–68)	57 (43–69)	0 (0–19)	98 (91–100)	70 (60–79)	53 (32–73)	75 (55–88)
	MultilayerPerceptronClassifier	44 (28–63)	67 (39–86)	47 (27–68)	69 (55–80)	6 (1–28)	91 (81–96)	97 (91–99)	63 (41–81)	88 (69–96)

See the Methods section (*Model training and validation*) for a full description of test sets: combined country, between country, and within country. The adjustment for imperfect sensitivity has some limitations given the SCORE study includes communities with baseline high prevalence of infection, including an inability to adjust communities with very high baseline prevalence (given lack of data to inform this estimate) and a less balanced training and test set. The analysis was not possible for the prevalence hotspot classifiers in Mozambique because all communities were prevalence hotspots after correction for test sensitivity.

*The between country test set (Mozambique) for *S. haematobium*, between country test set (Côte d'Ivoire) for *S. mansoni*, and within country test set (Tanzania) for *S. mansoni* each contained under 50 communities.

Table S26: Specificity (with 95% CI) of each model trained on epidemiologic and secondary data sets, by hotspot definition and test set, with prevalence variables corrected for estimated test sensitivity.

Species		*S. haematobium*			*S. mansoni*					
		Combined	Between	Within	Combined	Between			Within	
Test data country			MOZ*	NER		CIV*	KEN	TZA	KEN	TZA*
Prevalence hotspot	EnsembleClassifier	83 (63–93)	—	61 (42–76)	5 (1–25)	100 (77–100)	0 (-0–8)	0 (-0–39)	30 (11–60)	0 (0–56)
	LogisticRegression	78 (58–90)	—	61 (42–76)	0 (0–17)	15 (4–42)	2 (0–12)	0 (-0–39)	20 (6–51)	0 (0–56)
	ElasticNetLogistic	78 (58–90)	—	61 (42–76)	0 (0–17)	100 (77–100)	0 (-0–8)	0 (-0–39)	30 (11–60)	0 (0–56)
	RandomForestClassifier	83 (63–93)	—	61 (42–76)	11 (3–31)	0 (-0–23)	0 (-0–8)	0 (-0–39)	20 (6–51)	0 (0–56)
	GradientBoostingClassifier	70 (49–84)	—	61 (42–76)	0 (0–17)	31 (13–58)	31 (20–46)	33 (10–70)	50 (24–76)	0 (0–56)
	SupportVectorClassifier	78 (58–90)	—	61 (42–76)	0 (0–17)	100 (77–100)	0 (-0–8)	0 (-0–39)	0 (0–28)	0 (0–56)
	MultilayerPerceptronClassifier	91 (73–98)	—	68 (49–82)	47 (27–68)	92 (67–99)	0 (-0–8)	0 (-0–39)	20 (6–51)	0 (0–56)
	EnsembleRegressor	70 (49–84)	—	54 (36–70)	0 (0–17)	100 (77–100)	2 (0–12)	0 (-0–39)	10 (2–40)	0 (0–56)
	LinearRegression	48 (29–67)	—	36 (21–54)	11 (3–31)	0 (-0–23)	0 (-0–8)	17 (3–56)	30 (11–60)	0 (0–56)
	ElasticNetLinear	57 (37–74)	—	36 (21–54)	21 (9–43)	62 (36–82)	0 (-0–8)	17 (3–56)	30 (11–60)	0 (0–56)
	RandomForestRegressor	61 (41–78)	—	50 (33–67)	16 (6–38)	0 (-0–23)	0 (-0–8)	17 (3–56)	10 (2–40)	0 (0–56)
	GradientBoostingRegressor	74 (54–87)	—	50 (33–67)	0 (0–17)	0 (-0–23)	0 (-0–8)	0 (-0–39)	30 (11–60)	0 (0–56)
	SupportVectorRegressor	65 (45–81)	—	64 (46–79)	37 (19–59)	100 (77–100)	0 (-0–8)	0 (-0–39)	0 (0–28)	0 (0–56)
	MultilayerPerceptronRegressor	74 (54–87)	—	54 (36–70)	0 (0–17)	100 (77–100)	51 (37–65)	0 (-0–39)	20 (6–51)	0 (0–56)
Intensity hotspot	EnsembleClassifier	96 (88–99)	100 (51–100)	96 (87–99)	54 (38–70)	100 (85–100)	8 (4–16)	38 (18–61)	68 (46–85)	17 (3–56)
	LogisticRegression	98 (91–100)	100 (51–100)	94 (84–98)	17 (8–33)	100 (85–100)	8 (4–16)	69 (44–86)	32 (15–54)	17 (3–56)
	ElasticNetLogistic	96 (88–99)	50 (15–85)	94 (84–98)	0 (0–10)	100 (85–100)	0 (0–5)	25 (10–49)	42 (23–64)	17 (3–56)
	RandomForestClassifier	96 (88–99)	75 (30–95)	96 (87–99)	51 (36–67)	95 (77–99)	13 (7–23)	50 (28–72)	63 (41–81)	0 (-0–39)
	GradientBoostingClassifier	86 (74–93)	50 (15–85)	92 (82–97)	60 (44–74)	100 (85–100)	0 (0–5)	44 (23–67)	79 (57–91)	17 (3–56)
	SupportVectorClassifier	91 (81–96)	100 (51–100)	96 (87–99)	0 (0–10)	100 (85–100)	12 (6–21)	19 (7–43)	53 (32–73)	17 (3–56)
	MultilayerPerceptronClassifier	89 (79–95)	100 (51–100)	92 (82–97)	63 (46–77)	71 (50–86)	4 (1–11)	0 (0–19)	53 (32–73)	17 (3–56)
Persistent hotspot	EnsembleClassifier	88 (75–95)	88 (53–98)	73 (58–84)	88 (76–94)	100 (86–100)	9 (6–15)	38 (23–55)	82 (68–91)	25 (9–53)
	LogisticRegression	88 (75–95)	88 (53–98)	61 (47–74)	70 (57–80)	100 (86–100)	0 (0–3)	88 (72–95)	80 (65–90)	25 (9–53)
	ElasticNetLogistic	83 (69–92)	0 (0–32)	66 (51–78)	75 (62–84)	100 (86–100)	0 (0–3)	6 (2–20)	78 (62–88)	0 (0–24)
	RandomForestClassifier	90 (78–96)	100 (68–100)	68 (53–80)	80 (68–89)	96 (79–99)	14 (9–20)	62 (45–77)	88 (74–95)	17 (5–45)
	GradientBoostingClassifier	76 (61–87)	88 (53–98)	64 (49–76)	64 (51–76)	74 (54–87)	44 (36–52)	84 (68–93)	85 (71–93)	25 (9–53)
	SupportVectorClassifier	76 (61–87)	100 (68–100)	75 (61–85)	89 (79–95)	100 (86–100)	9 (5–14)	41 (26–58)	78 (62–88)	42 (19–68)
	MultilayerPerceptronClassifier	79 (64–88)	50 (22–78)	77 (63–87)	75 (62–84)	100 (86–100)	13 (8–20)	6 (2–20)	72 (57–84)	42 (19–68)

See the Methods section (*Model training and validation*) for a full description of test sets: combined country, between country, and within country.
The adjustment for imperfect sensitivity has some limitations given the SCORE study includes communities with baseline high prevalence of infection, including an inability to adjust communities with very high baseline prevalence (given lack of data to inform this estimate) and a less balanced training and test set. The analysis was not possible for the prevalence hotspot classifiers in Mozambique because all communities were prevalence hotspots after correction for test sensitivity.
*The between country test set (Mozambique) for *S. haematobium*, between country test set (Côte d'Ivoire) for *S. mansoni*, and within country test set (Tanzania) for *S. mansoni* each contained under 50 communities.

SI References

1. D. G. Colley, *et al.*, SCORE *S. mansoni* Cluster Randomized Trial. *ClinEpiDB* (2021) (July 20, 2022).

2. D. G. Colley, N. Kittur, J. D. Castleman, SCORE Niger *S. haematobium* Cluster Randomized Trial. *ClinEpiDB* (2021) (July 20, 2022).

3. D. G. Colley, N. Kittur, J. D. Castleman, A. E. Phillips, P. H. Gazzinelli-Guimaraes, SCORE Mozambique *S. haematobium* Cluster Randomized Trial. *ClinEpiDB* (2021) (July 20, 2022).

4. C. H. King, *et al.*, Impact of Different Mass Drug Administration Strategies for Gaining and Sustaining Control of *Schistosoma mansoni* and *Schistosoma haematobium* Infection in Africa. *Am J Trop Med Hyg* **103**, 14–23 (2020).

5. D. J. Corsi, M. Neuman, J. E. Finlay, S. V. Subramanian, Demographic and health surveys: a profile. *Int J Epidemiol* **41**, 1602–1613 (2012).

6. A. Osgood-Zimmerman, *et al.*, Mapping child growth failure in Africa between 2000 and 2015. *Nature* **555**, 41–47 (2018).

7. J. F. Mosser, *et al.*, Mapping diphtheria-pertussis-tetanus vaccine coverage in Africa, 2000–2016: a spatial and temporal modelling study. *The Lancet* **393**, 1843–1855 (2019).

8. A. Deshpande, *et al.*, Mapping geographical inequalities in access to drinking water and sanitation facilities in low-income and middle-income countries, 2000–17. *The Lancet Global Health* **8**, e1162–e1185 (2020).

9. N. Graetz, *et al.*, Mapping disparities in education across low- and middle-income countries. *Nature* **577**, 235–238 (2020).

10. R. Burstein, *et al.*, Mapping 123 million neonatal, infant and child deaths between 2000 and 2017. *Nature* **574**, 353–358 (2019).

11. Didan, Kamel, MOD13Q1 MODIS/Terra Vegetation Indices 16-Day L3 Global 250m SIN Grid V006 (2015) https:/doi.org/10.5067/MODIS/MOD13Q1.006 (July 20, 2022).

12. M. Buchhorn, *et al.*, Copernicus Global Land Cover Layers—Collection 2. *Remote Sensing* **12**, 1044 (2020).

13. S. E. Fick, R. J. Hijmans, WorldClim 2: new 1-km spatial resolution climate surfaces for global land areas. *International Journal of Climatology* **37**, 4302–4315 (2017).

14. Center For International Earth Science Information Network-CIESIN-Columbia University, Gridded Population of the World, Version 4 (GPWv4): Population Density Adjusted to Match 2015 Revision UN WPP Country Totals, Revision 11 (2018) https:/doi.org/10.7927/H4F47M65 (July 20, 2022).

15. Institute for Health Metrics and Evaluation (IHME), Low- and Middle-Income Drinking Country Water and Sanitation Facilities Access Geospatial Estimates 2000-2017 (2020) https:/doi.org/10.6069/19PR-QE36 (August 16, 2023).

16. Institute for Health Metrics and Evaluation (IHME), Africa Diphtheria-Pertussis-Tetanus Vaccine Coverage Geospatial Estimates 2000-2016 https:/doi.org/10.6069/WGD8-W576 (August 16, 2023).

17. Institute for Health Metrics and Evaluation (IHME), Low- and Middle-Income Country Educational Attainment Geospatial Estimates 2000-2017 https:/doi.org/10.6069/1S4Y-FP03 (August 16, 2023).

18. Institute for Health Metrics and Evaluation (IHME), Low- and Middle-Income Country Neonatal, Infant, and Under-5 Mortality Geospatial Estimates 2000-2017 https:/doi.org/10.6069/9ABZ-XG84 (August 16, 2023).

19. G. Chi, H. Fang, S. Chatterjee, J. E. Blumenstock, Microestimates of wealth for all low- and middle-income countries. *Proceedings of the National Academy of Sciences* **119**, e2113658119 (2022).

20. F. Pedregosa, *et al.*, Scikit-learn: Machine Learning in Python. *Journal of Machine Learning Research* **12**, 2825–2830 (2011).

21. T. Chen, C. Guestrin, XGBoost: A Scalable Tree Boosting System in *Proceedings of the 22nd ACM SIGKDD International Conference on Knowledge Discovery and Data Mining*, KDD ’16., (Association for Computing Machinery, 2016), pp. 785–794.

22. O. Bärenbold, *et al.*, Estimating true prevalence of *Schistosoma mansoni* from population summary measures based on the Kato-Katz diagnostic technique. *PLOS Neglected Tropical Diseases* **15**, e0009310 (2021).

23. N. Midzi, *et al.*, Accuracy of different diagnostic techniques for *Schistosoma haematobium* to estimate treatment needs in Zimbabwe: Application of a hierarchical Bayesian egg count model. *PLOS Neglected Tropical Diseases* **14**, e0008451 (2020).

TRIPOD Checklist

Section/Topic			Checklist Item	Page
**Title and abstract**				
Title	1	D;V	Identify the study as developing and/or validating a multivariable prediction model, the target population, and the outcome to be predicted.	1
Abstract	2	D;V	Provide a summary of objectives, study design, setting, participants, sample size, predictors, outcome, statistical analysis, results, and conclusions.	1
**Introduction**				
Background and objectives	3a	D;V	Explain the medical context (including whether diagnostic or prognostic) and rationale for developing or validating the multivariable prediction model, including references to existing models.	1–2
	3b	D;V	Specify the objectives, including whether the study describes the development or validation of the model or both.	1–2
**Methods**				
Source of data	4a	D;V	Describe the study design or source of data (e.g., randomized trial, cohort, or registry data), separately for the development and validation data sets, if applicable.	2, 7, Appendix
	4b	D;V	Specify the key study dates, including start of accrual; end of accrual; and, if applicable, end of follow-up.	7
Participants	5a	D;V	Specify key elements of the study setting (e.g., primary care, secondary care, general population) including number and location of centres.	2, 7, Table 1
	5b	D;V	Describe eligibility criteria for participants.	2, 7
	5c	D;V	Give details of treatments received, if relevant.	Table 1, Appendix p3
Outcome	6a	D;V	Clearly define the outcome that is predicted by the prediction model, including how and when assessed.	2, 7
	6b	D;V	Report any actions to blind assessment of the outcome to be predicted.	N/A, Appendix
Predictors	7a	D;V	Clearly define all predictors used in developing or validating the multivariable prediction model, including how and when they were measured.	8, Appendix p3-5
	7b	D;V	Report any actions to blind assessment of predictors for the outcome and other predictors.	N/A, Appendix
Sample size	8	D;V	Explain how the study size was arrived at.	2, Figure S1
Missing data	9	D;V	Describe how missing data were handled (e.g., complete-case analysis, single imputation, multiple imputation) with details of any imputation method.	Appendix p5
Statistical analysis methods	10a	D	Describe how predictors were handled in the analyses.	8, Appendix p3-5
	10b	D	Specify type of model, all model-building procedures (including any predictor selection), and method for internal validation.	7–8, Appendix p5
	10c	V	For validation, describe how the predictions were calculated.	7-8
	10d	D;V	Specify all measures used to assess model performance and, if relevant, to compare multiple models.	8
	10e	V	Describe any model updating (e.g., recalibration) arising from the validation, if done.	N/A

Risk groups	11	D;V	Provide details on how risk groups were created, if done.	N/A
Development vs. validation	12	V	For validation, identify any differences from the development data in setting, eligibility criteria, outcome, and predictors.	7, Figure S11-S15
**Results**				
Participants	.3a	D;V	Describe the flow of participants through the study, including the number of participants with and without the outcome and, if applicable, a summary of the follow-up time. A diagram may be helpful.	2, Table 1, Figure S1
	.3b	D;V	Describe the characteristics of the participants (basic demographics, clinical features, available predictors), including the number of participants with missing data for predictors and outcome.	2, Table 1
	.3c	V	For validation, show a comparison with the development data of the distribution of important variables (demographics, predictors and outcome).	Figure S11-S15
Model development	.4a	D	Specify the number of participants and outcome events in each analysis.	2, Table 1
	.4b	D	If done, report the unadjusted association between each candidate predictor and outcome.	Table S12
Model specification	.5a	D	Present the full prediction model to allow predictions for individuals (i.e., all regression coefficients, and model intercept or baseline survival at a given time point).	Table S10-S11
	.5b	D	Explain how to the use the prediction model.	3
Model performance	16	D;V	Report performance measures (with CIs) for the prediction model.	2–3, Table S3-S9
Model-updating	17	V	If done, report the results from any model updating (i.e., model specification, model performance).	N/A
**Discussion**				
Limitations	18	D;V	Discuss any limitations of the study (such as nonrepresentative sample, few events per predictor, missing data).	7
Interpretation	.9a	V	For validation, discuss the results with reference to performance in the development data, and any other validation data.	6
	.9b	D;V	Give an overall interpretation of the results, considering objectives, limitations, results from similar studies, and other relevant evidence.	4–7
Implications	20	D;V	Discuss the potential clinical use of the model and implications for future research.	6–7
**Other information**				
Supplementary information	21	D;V	Provide information about the availability of supplementary resources, such as study protocol, Web calculator, and data sets.	8
Funding	22	D;V	Give the source of funding and the role of the funders for the present study.	8

*Items relevant only to the development of a prediction model are denoted by D, items relating solely to a validation of a prediction model are denoted by V, and items relating to both are denoted D;V. We recommend using the TRIPOD Checklist in conjunction with the TRIPOD Explanation and Elaboration document.
